# A novel approach to modelling transcriptional heterogeneity identifies the oncogene candidate *CBX2* in invasive breast carcinoma

**DOI:** 10.1038/s41416-019-0387-8

**Published:** 2019-03-01

**Authors:** Daniel G. Piqué, Cristina Montagna, John M. Greally, Jessica C. Mar

**Affiliations:** 1 Department of Systems and Computational Biology, Albert Einstein College of Medicine, 1300 Morris Park Avenue, Bronx, NY 10461 USA; 2 Department of Genetics, Albert Einstein College of Medicine, 1300 Morris Park Avenue, Bronx, NY 10461 USA; 3 Department of Epidemiology and Population Health, Albert Einstein College of Medicine, 1300 Morris Park Avenue, Bronx, NY 10461 USA; 4 Australian Institute for Bioengineering and Nanotechnology, The University of Queensland, Queensland, QLD 4072 Australia

**Keywords:** Gene expression profiling, Software

## Abstract

**Background:**

Oncogenes promote the development of therapeutic targets against subsets of cancers. Only several hundred oncogenes have been identified, primarily via mutation-based approaches, in the human genome. Transcriptional overexpression is a less-explored mechanism through which oncogenes can arise.

**Methods:**

Here, a new statistical approach, termed oncomix, which captures transcriptional heterogeneity in tumour and adjacent normal (i.e., tumour-free) mRNA expression profiles, was developed to identify oncogene candidates that were overexpressed in a subset of breast tumours.

**Results:**

Intronic DNA methylation was strongly associated with the overexpression of chromobox 2 (*CBX2*), an oncogene candidate that was identified using our method but not through prior analytical approaches. *CBX2* overexpression in breast tumours was associated with the upregulation of genes involved in cell cycle progression and with poorer 5-year survival. The predicted function of *CBX2* was confirmed in vitro, providing the first experimental evidence that *CBX2* promotes breast cancer cell growth.

**Conclusions:**

Oncomix is a novel approach that captures transcriptional heterogeneity between tumour and adjacent normal tissue, and that has the potential to uncover therapeutic targets that benefit subsets of cancer patients. *CBX2* is an oncogene candidate that should be further explored as a potential drug target for aggressive types of breast cancer.

## Background

Oncogenesis is driven by a complex and intricately controlled programme of gene expression where oncogenes are the expressed genes that promote tumour development. The first set of oncogenes were discovered in retroviruses that incorporated human growth factors, such as *src*, into their viral genome.^1–3^ The identification of amplified or mutated oncogenes in the tumours of certain cancer patients has led to the development of effective molecular therapeutic strategies that extend the life of these patients. For example, trastuzumab, an anti-HER2 antibody, extends the overall lifespan for approximately 20% of breast cancer patients whose often-aggressive tumours overexpress *ERBB2*, the gene that encodes the HER2 protein.^4^ However, HER2-targeted therapies often result in treatment resistance and thus additional therapeutic targets are required to adequately treat HER2^+^ breast cancer, among other subtypes.

Variability in the response of patients to current therapeutic strategies represents a major bottleneck to reducing cancer mortality rates globally. Understanding how tumour heterogeneity impacts the transcriptional regulatory programmes that control oncogenesis is the key to addressing this issue and is currently what drives most programmes in personalised medicine. The availability of genome-wide gene expression data from matched tumour and adjacent normal tissue of large patient populations provides a valuable resource for developing new approaches for identifying oncogenes that are likely to have pivotal roles in important clinical outcomes such as chemoresistance. For example, previous studies have identified survival-related biomarkers in ovarian cancer based on bimodal gene expression profiles detected in large datasets of tumours.^5^ These studies recognise the limitations of the unimodal assumption made by many statistical tests and have taken advantage of the inherent heterogeneity in gene expression profiles to discover new subtypes.

Examples of methods that exploit heterogeneity between tumour and adjacent normal tissue include Cancer Outlier Profile Analysis (COPA)^6^ and mCOPA^7^ which are both used to detect gene fusions and tumour outliers. However, these approaches have two major limitations. First, most applications of mixture modelling for gene expression, with one exception^8^ have been developed using data derived from microarrays, which have a limited range of expression values, particularly for highly expressed genes, and unlike RNA-sequencing (RNA-seq), are limited for quantifying transcript levels at high resolution.^9^ Second, tools developed for outlier detection from paired tumour-normal mRNA samples, such as COPA^6,10^ and Profile Analysis using Clustering and Kurtosis (PACK),^11^ are sensitive to the proportion of samples that are distinguished as ‘outliers’^8^ and, in the case of COPA, require setting a tuning parameter. In addition, existing methods for outlier detection are designed to screen out individual tumour samples rather than identify genes that reflect new patient subgroupings.

In this study, we developed a statistical approach termed oncomix to identify oncogene candidates (OCs) in RNA-seq data. This approach detects OCs based on the presence of low expression in normal tissue and overexpression in a subset of patient tumours. Our approach capitalises on the heterogeneity present in matched tumour and normal gene expression data to identify OCs and then segregate patients into interpretable subgroups based on their expression of the OC. Oncomix is an unsupervised method where the size of the patient subgrouping is learned entirely from the data.

To demonstrate the utility of oncomix, we applied this approach to RNA-seq data from the breast cancer cohort of The Cancer Genome Atlas (TCGA) and identified a set of five OCs (*CBX2*, *NELL2*, *EPYC*, *SLC24A2*, and *LAG3*). To understand why these OCs were overexpressed in certain tumours, we developed predictive models using multiple molecular, genetic, and clinical variables from TCGA that highlighted potential regulators of OC overexpression. Novel computational and experimental evidence suggest that chromobox 2 (*CBX2*), one of the OCs that we identified, is associated with poorer clinical outcomes and functions as a regulator of breast tumour cell growth. In this study, we demonstrate the value of modelling transcriptional heterogeneity using matched tumour and normal tissue to identify new OCs. Our results indicate that *CBX2* may serve as a driver of breast cancer and represent a novel therapeutic target in aggressive subtypes of breast cancer, such as HER2^+^ and basal-like.

## Methods

### RNA data sources and sample selection

Fragments per kilobase of transcript per million mapped reads (FPKM) level 3 mRNA-sequencing data from invasive breast carcinoma and adjacent normal controls was downloaded from the Genomic Data Commons web server in November 2018 (version 0.13) using the GenomicDataCommons and TCGAbiolinks R packages using standard GDC pipelines (https://docs.gdc.cancer.gov/Data/Bioinformatics_Pipelines/Expression_mRNA_Pipeline/). The level 3 mRNA-sequencing data contains the calculated expression level of a gene for each sample. The FPKM output mapped to 56,716 ensembl gene ids and was converted to transcripts per million (TPM) and subsequently log_2_(TPM + 1) transformed to shrink the numeric range of the data. Genes that contain > 20% zero values were excluded, as genes with many zero values can result in the failure of mixture model algorithms to converge on a set of parameters. A total of 110 female patients from TCGA with RNA-seq data from both tumour and adjacent normal tissue were selected for further study. mRNA-sequencing data from endometrial, lung and prostate adenocarcinoma (Supplementary Figures 10-13) were downloaded and processed using the same tools and criteria.

### Benchmarking oncomix against limma and mCOPA

Differential expression between tumour and adjacent normal samples was performed using limma, an established method for performing a two-sample *t*-test in conjunction with empirical Bayes estimation.^12^ A total of 16,156 genes that had > 20% non-zero values for both tumour and adjacent normal samples were used and ranked using the t-statistic and resulting *p*-value. A ranking of 1 indicates the gene with the smallest *p*-value. Expression data for 16,156 genes from 220 paired tumour-adjacent normal samples was used as input into mCOPA. mCOPA requires the manual specification of percentiles and was run three times using the 70th, 80th, and 90th percentile. The 80th percentile results were displayed in Supplementary Figure 3, with the rationale that these would be most consistent with our requirement that at least 20% of samples appear in either the high or the low expression group.

### Differential expression and pathway overrepresentation analysis

Differentially expressed genes between two groups (e.g., tumours that do vs. do not overexpress *CBX2*) were identified using limma.^12^ The threshold used for differential expression was a Benjamini–Hochberg adjusted *q*-value of 0.0001 and a log_2_(fold change) > 1 or < − 1. POA was performed using 910 gene sets from three well-defined, manually curated pathway databases – Hallmark,^13^ KEGG^14^ and Reactome.^15^ Geneset databases were downloaded from MSigDB as GMT files in March 2017.^16^ For each OC, POA was performed separately for significantly upregulated and downregulated genes using Fisher’s exact test to facilitate interpretability and a stringent cut-off (Benjamini-Hochberg adjusted *q* < 1 × 10^−20^ and OR_95% CI_ > 20) was used to select highly enriched gene sets.

### Multiple logistic regression, variable selection and coefficient shrinkage using the elastic net

Multiple logistic regression was performed for each OC with binary response variables (normal or overexpressed OC mRNA levels in breast tumours) and complementary clinical, molecular and pathological datasets were used as covariates (see Supplementary Figure 4 for datasets and processing information). The output from the logistic regression model provides a weight, in the form of a *β*- coefficient, which estimates the influence for each predictor on the response variable, which in this case is the overexpression of the OC. How strong of an influence the predictor has on the response is estimated by the model, as well as the direction of this influence. To prevent model overfitting, the size of the model coefficients, whose effect was assumed to be additive, were regularised using the elastic net penalty and leave-one-out cross validation^17^ (see Supplementary Figure 5). The elastic net is a regularisation term that shrinks and selects model coefficients to prevent overfitting of data, particularly in settings when there are many predictor variables, and helps account for potential collinearities between covariates.^17^

The code for the implementation of this method is available in Supplementary File 2.

**Fig. 1:**
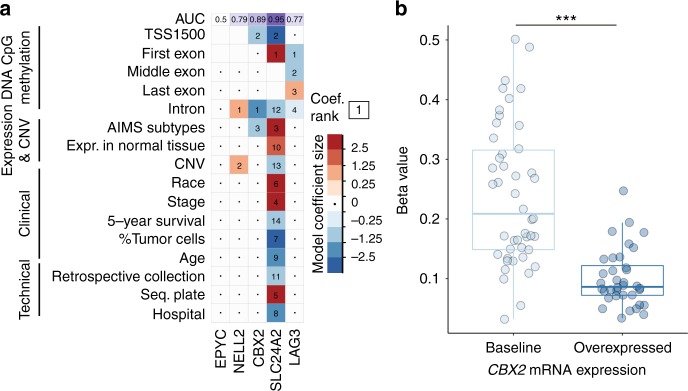
Multi-omic prediction of oncogene candidate mRNA overexpression in breast tumours. **a** Visualisation of model coefficient selection after regularised logistic regression on binarized (baseline or overexpressed) oncogene candidate mRNA expression levels in breast tumours. Deep blue squares indicate variables that contribute greatly to the prediction of the baseline expression state, whereas deep red squares indicate variables that contribute greatly to the prediction of the overexpressed state. The numbers in each cell indicate the rank of the absolute value of a coefficient relative to all other coefficients for that model, where 1 is the largest model coefficient. Variables not selected as part of the model are indicated with an interpunct (·). Blank cells indicate missing data for a given model. Each model was used to predict whether a sample overexpressed a given OC or not. These predictions were used to generate receiver operating curves, from which the area under the curve (AUC) was derived (top row, purple background). **b** Association of *CBX2* overexpression with DNA methylation beta values for the highest ranking logistic regression coefficient (an intronic CpG locus). DNA methylation values are grouped by level (either baseline or overexpressed) of *CBX2* mRNA expression in tumours. Statistical testing was performed using the Wilcoxon rank-sum test (***P-value < 1 × 10^−8^)

To validate the utility of the logistic regression models, each model was used to predict the probability of each patient overexpressing the OC in the dataset given her individual features. An area under the curve (AUC) value was generated for each of the five models that predicted overexpression of each OC (Fig. 1a, top panel). AUC values > 0.8 suggest an excellent fit, whereas values between 0.7 and 0.8 suggest a good fit.^18^ Models for two out of the five OCs, including the model for *CBX2*, had an AUC > 0.8.

### *CBX2* siRNA knockdown experiments and analysis of cellular growth rate

MCF7 cells were obtained from ATCC (#HTB-22). Cells were grown in Dulbecco’s modified Eagle’s medium supplemented with 5% fetal bovine serum and 0.01 mg/ml human recombinant insulin (Sigma) and incubated in 5% CO_2_/37 °C. For silencing of *CBX2*, the siRNA SMARTpool (L-008357 -Dharmacon, Lafayette USA) was used. On-target *CBX2* oligonucleotides were used for gene-specific downregulation and the same MCF7 cells transfected with the Non-Targeting (Scramble) siRNA Control Pools were used as a reference control for all experiments. SiRNA pools were resuspended according to the manufacturer’s protocol in RNase-free 1 × siRNA Buffer at a final concentration of 20 mM. Cells were transfected using DharmaFECT-4 Transfection Reagent according to the manufacturer’s instructions. After transfection, cells grew for 48 h before the analysis of specific endpoints.

For the growth curve analysis, MCF7 cells silenced with the siCBX2 SMARTpool and scramble controls were plated at ~17,000 cells/cm^2^ in 24-well plates, incubated at 37 °C for 48 h and the cell number counted in duplicate every 24 h for 5 days. All experiments were repeated three times in independent biological triplicates. MCF7 were routinely analysed to ensure lack of mycoplasma contamination by 4′,6-diamidino-2-phenylindole staining. A three-way between-subjects ANOVA without interaction terms was conducted to test the null hypothesis that siRNA has no effect on cellular growth rate. The independent variables, all categorical, were the siRNA, the biological replicate and the day post transfection. The MCF7 cell line was authenticated using the GenePrint 24 system (Catalogue number B1870, Promega) and analysed using the GeneMarker 1.75 software (SoftGenetics). Cell line genotypes showed 100% identity to MCF7 cell lines (results available upon request).

### RNA isolation and cDNA synthesis to evaluate *CBX2* levels

MCF7 siCBX2 and siScramble were established as described above and plated in 6-well plates at ~17,000 cells/cm^2^ for 48 h. Cells were then analysed at 72–120–168 h post transfection. The cells were then lysed directly on the plate with Qiazol lysis reagent (Qiagen, Valencia, CA) and placed at − 80 °C until all samples were ready for RNA extraction. Total RNA was isolated using the miRNeasy kit (Qiagen, Valencia, CA). cDNA was reverse-transcribed from 5 μg of total RNA using random primers and SuperScript II Reverse Transcriptase (Invitrogen). *CBX2* and *GAPDH* primers were designed with Primer3 software (sequences listed below). Real-time quantitative reverse transcriptase-PCR was performed using Applied Biosystems Fast SYBR Green Master Mix and the StepOnePlus Real-Time PCR System (Life Technologies Corp., Carlsbad, CA, USA). Data normalisation and analysis were performed as previously described (Acosta et al.).^19^

CBX2fw: 5′-GGCTGGTCCTCCAAACATAA-3′

CBX2rev: 5′-GCACCTCCTTCTCATGTTCC-3′

GAPDHfw: 5′-CCACATCGCTCAGACACCAT-3′

GAPDHrev: 5′-CCAGGCGCCCAATACG-3′

## Results

### Deriving a new transcription-driven approach to discover OCs that are specific for subgroups of breast cancer patients

**Fig. 2:**
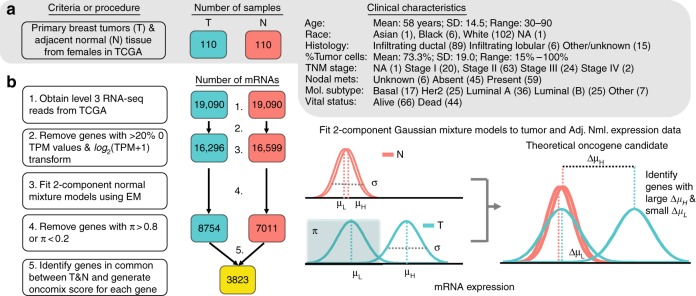
Study design to identify oncogene candidates from breast carcinoma and adjacent normal RNA-sequencing samples. **a** Clinical characteristics of the study cohort of 110 female patients with invasive breast carcinoma. Each of these patients have RNA-sequencing data available from both the primary breast tumour (T) and adjacent normal breast tissue (N). The number of patient samples is indicated within boxes coloured either teal for tumour (T) samples, or orange for adjacent normal (N) samples. The bold type highlights the most common class for each patient characteristic. **b** Workflow of RNA-seq gene filtering based on transcripts per million mapped reads (TPM). The numbered statements on the right reflect the steps used to transform and filter the data for subsequent analysis. Level 3 mRNA expression data refers to the degree of expression quantification performed by TCGA (see Methods). The number of genes at each step of the workflow is indicated within the coloured boxes. An illustration of a two-component Gaussian mixture model (GMM), shown in teal, used to separately fit each gene’s log_2_(TPM + 1) values for tumour and adjacent normal controls. GMMs yield several distinct parameters; namely, *π* is the proportion of samples under the Gaussian associated with lower expression values, *μ*_L_ and *μ*_H_ are the means of the curves that fit lower and higher expression values, respectively, and *σ* is the common SD of the two Gaussians. The additional subscript (T or N) refers to whether the sample parameters are derived from tumour or adjacent normal expression data. Note that the threshold between baseline and overexpressed is defined by the boundary set from the mixture models in the tumour samples and is the point at which the probability of a sample belonging to either the low or high expression group is equal to 0.5. EM = expectation maximisation

An OC can be defined as a gene that is highly expressed in a subset of tumour samples and has uniformly low expression in adjacent normal tissue. Our primary objective was to test whether such genes could be found in a cancer patient dataset. RNA-seq data from 110 breast cancer patients was selected from TCGA as this was the subset with both tumour and adjacent normal pairs sequenced for Caucasian females (Fig. 2a). It was important for our study to avoid differences in expression values between adjacent normal and tumour tissue that might simply result from person-to-person variation in gene expression between different individuals. To ensure that the mixture models could be stably fit to the data, lowly expressed genes were filtered (see Methods, Fig. 2b). Two-component mixture models were fit to each transcript for both tumour and adjacent normal samples independently (Fig. 2b). For each transcript, tumour and normal samples were separately classified as expressing either low or high levels of gene expression based on the mixture component with the largest probability density. This series of filtering steps yielded a set of 3823 genes that were further filtered, as described below, to identify a set of high-confidence OCs.

### Oncomix identified five genes with an oncogene-like pattern of expression

**Fig. 3:**
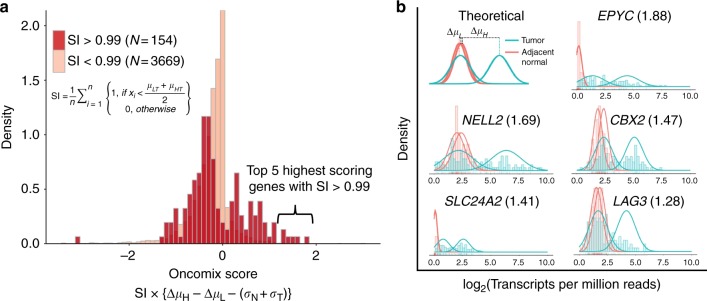
Identification of oncogene candidates using RNA-sequencing data from primary invasive breast carcinomas and adjacent normal breast tissue. **a** The distribution of the oncomix scores separated by genes with a selectivity index (SI) above and below 0.99. Larger oncomix scores correspond to genes that more closely resemble the profile of a theoretical oncogene candidate. **b** Superimposed histograms of expression values from tumour (teal) and adjacent normal (red) samples for the 5 genes with the highest oncomix score and a SI > 0.99. The best fitting mixture model is shown for each selected gene. The HUGO gene symbol for each gene is displayed for each histogram, with the oncomix score in parentheses. A theoretical model for an ideal oncogene candidate is shown in the upper left. The *y* axis represents density and the *x* axis represents log_2_(Transcripts Per Million + 1) reads. T = primary breast tumour, N = adjacent normal breast tissue, *n =* total number of adjacent normal samples

Our statistical approach, oncomix, detects a distinct bimodal pattern of gene expression across tumours. To identify OCs that matched these specific patterns from the total pool of genes, two metrics were derived from the mixture model parameters. First, a selectivity index (SI) distinguishes those genes that are overexpressed in a clearly defined group of patient tumours. A threshold of SI > 0.99 was set based on the observed distribution of the SI values. Examination of the gene expression data from known oncogenes annotated in the Cancer Gene Census (Supplementary Figure 1) with an SI > 0.99 highlighted oncogenes, such as *ERBB2*, with a known role in breast carcinogenesis. The SI was used in combination with other mixture model parameters to calculate the oncomix score, which ranks genes based on their similarity to a theoretically ideal oncogene (Fig. 3a). The distribution of expression levels for the five genes with the highest oncomix score each demonstrate a clear and distinct subgroup of tumours that overexpress each gene (Fig. 3b). Of note, oncomix did not detect certain genes that are canonically known to be activated via overexpression (e.g., *MYC*, *CCND1*, *FGFR1*, *FGFR2*),^20^ in part because of their high levels of expression in adjacent normal tissue (mean log_2_(TPM) = 6.796) relative to the expression of the top 5 OCs detected in normal tissue (mean log_2_(TPM) = 1.227). Therefore, oncomix is an approach for ranking genes that are overexpressed in subsets of tumours and forms a basis for identifying OCs.

As oncomix is based on ranking genes rather than hypothesis testing, deriving exact power calculations are challenging to apply. A simulation study was performed to estimate the power associated with the oncomix procedure that was applied to the 110 tumour-adjacent normal pairs using the thresholds adopted in this study. At a Type 1 (*ɑ*) level of 1.91 × 10^−6^ (Student’s one-sided *t*-test), the power is estimated to be 0.723 (based on 1000 simulations, see Supplementary Methods and Supplementary Figure 2)

A literature search of the 5 OCs discovered by oncomix revealed that oncogene-like features have previously been linked to three of these genes (Supplementary Table 1). *CBX2* and neural EGFL-like 2 (*NELL2*) have been shown to promote invasion, metastasis, or cell division in a variety of in vivo and in vitro models of cancer. For example, the gene *CBX2* was recently shown to be highly expressed in both androgen-independent, late-stage prostate cancers (PrCa) and distant PrCa metastases.^21^ *CBX2* is a member of the polycomb repressive complex (PRC) and expression of this gene and its protein product is negatively associated with breast cancer survival^22,23^ In addition, *NELL2* encodes a neural cell growth factor whose expression is positively regulated by oestrogen, and that promotes invasion of breast cancer cells.^24,25^ The sympathetic nervous system has also been shown to promote breast cancer metastasis from primary tumours.^26^ *LAG3* encodes a molecule expressed on the surface of lymphocytes and its expression is associated with favourable outcomes in breast cancer.^27^ Furthermore, the five OCs identified by oncomix represent a unique set of genes that are not reliably detectable by existing approaches, such as limma^12^ and mCOPA,^7^ which rank genes based on expression profiles between tumour and adjacent normal samples (Supplementary Table 1 and Supplementary Figure 3). None of the five OCs identified overlapped with genes found in AIMS, oncotypeDx or mammaprint.^28–30^ These results lend support to the premise for our method, which models population-level patterns of gene expression in subgroups of patients to identify unique OCs.

### Tumours that overexpress *CBX2* manifest transcriptome-wide changes in the expression of cancer-relevant pathways

Oncogenes are often members of molecular signalling pathways and can drive changes in cellular processes, such as cell proliferation, which promote carcinogenesis. Therefore, we sought to determine whether tumours that overexpressed an OC harboured carcinogenesis-related transcriptional changes relative to tumours that did not overexpress a given OC. For each OC, patients were classified into two groups based on whether their tumour overexpressed the OC or not. The overexpression of two of the OCs, *EPYC* and *NELL2*, were associated with minor changes in the cancer transcriptome ( ≤ 5 differentially expressed transcripts, Supplementary Table 2). Among the remaining three OCs, there were ≥ 90 differentially expressed transcripts.

To characterise the genes that were differentially expressed in tumours that overexpressed each OC, a pathway overrepresentation analysis (POA) was performed using a stringent threshold (see Methods). For all five OCs, no gene sets were enriched when examining only the genes downregulated in tumours that overexpress the OC. Significantly enriched gene sets were present for upregulated genes among the OCs *CBX2* and *SLC24A2* (Supplementary Table 3). Pathways that were overrepresented in tumours that overexpress *CBX2* include cell cycle checkpoint regulation. These results are consistent with previous results that showed differential expression of genes within cell cycle-related pathways following small interfering RNA (siRNA)-mediated *CBX2* silencing in PrCa cells.^21^ *CBX2* overexpression was associated with the upregulation of genes such as *KIF2C* (log_2_(fold change) = 1.57; *q* = 9.00 × 10^−7^), a member of the kinesin family of proteins that are important for mediating microtubule dynamics during mitosis^31^ (Supplementary Table 4). The *KIF2C* gene has been demonstrated to be regulated by EZH2, the catalytic subunit of the PRC2, in the context of melanoma, which supports our findings of a link between the *CBX2*, a member of the PRC1 complex, and *KIF2C* expression.^32^ These analyses demonstrate that two out of the five genes identified to be overexpressed in a subset of patient tumours may alter the breast cancer transcriptome in a biologically plausible manner.

### Prediction of OC overexpression reveals that molecular features are more influential than clinicopathologic features

We next sought to identify the biological and clinical features that could contribute to the overexpression of the five identified OCs in a subset of breast tumours (Fig. 1a). The predictor variables used in the regularized multiple logistic regression model represented four broad categories: DNA methylation, expression and copy number, clinicopathologic and technical variables (see Supplementary Figure 4 for datasets and processing information and Supplementary Figure 5 for a model-fitting schematic). For two out of the five OCs, including *CBX2*, intronic methylation was the most predictive covariate. Furthermore, the distribution of CpG *β*-values for the single most influential covariate in the *CBX2* model, a CpG site located within the second intron of the *CBX2* gene (Supplementary Figure 6), showed a clear reduction in DNA methylation (*P*-value < 1 × 10^−8^, Wilcoxon rank-sum test) in breast tumours that overexpress *CBX2* (Fig. 1b). This intronic CpG site overlaps with the binding site for an oncogenic transcription factor, JunD, which promotes cancer cell proliferation.^33^ In addition, the molecular subtype, as inferred using Absolute Intrinsic Molecular Subtyping (AIMS) method,^30^ was strongly associated with OC overexpression (two-way analysis of variance (ANOVA), F(1, 107), AIMS: *P*-value = 8.9 × 10^−4^, intronic CpG methyl: *P*-value = 1.4 × 10^−9^) (Supplementary Figure 7). No statistically significant associations were found between oncogene driver mutations and OC overexpression (Supplementary Figure 8). Relative to the molecular variables, clinicopathologic characteristics, such as cell subtype composition, patient age and the presence of metastases, were weakly associated with OC overexpression, indicated by the lighter colours and absent within-cell numbers in Fig. 1a. These analyses demonstrate that OC overexpression is strongly associated with molecular covariates, particularly DNA CpG methylation.

### *CBX2* is overexpressed in aggressive breast carcinomas and is associated with poor survival

**Fig. 4:**
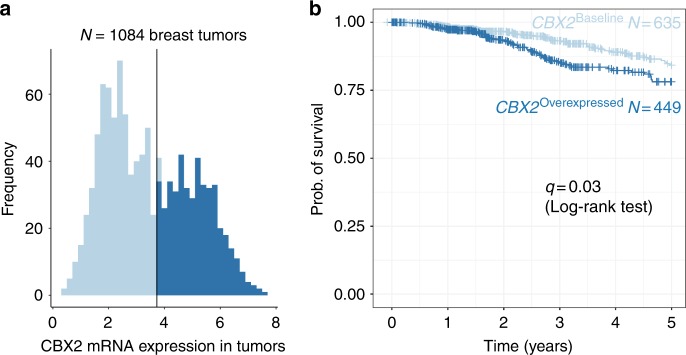
Overexpression of *CBX2* in primary breast tumours is associated with lower rates of survival. **a** Tumours that overexpress *CBX2* are shown in dark blue, and tumours that express baseline levels of *CBX2* are shown in light blue. The tumours were classified using the same boundary that was defined for the original 110 tumour samples. **b** A Kaplan–Meier survival curve for 5-year survival rates for 1084 patients with breast tumours from TCGA is shown. A log-rank test was performed to check for differences in survival between the two tumour types (*q* = 0.03)

Post hoc visualisation from the logistic regression model from Fig. 3a revealed a positive relationship between the aggressiveness of the AIMS breast tumour subtype and the proportion of patients who expressed *CBX2* within each subtype (Supplementary Figure 7).^30^ Expression of *CBX2* is not part of the mRNA expression-based AIMS classification scheme, which highlights the potential utility of *CBX2* in the identification and molecular subtyping of aggressive breast tumours. This is the first report using RNA-seq data to show that *CBX2* is enriched in basal-like and HER2^+^ tumours and our result is supported by a previous study that also found increased *CBX2* expression in basal-like breast tumours in a microarray mRNA breast cancer dataset.^22,34^ *CBX2* mRNA expression may therefore serve as a marker of aggressive breast cancer subtypes, such as basal-like carcinomas, which lack a reliable molecular marker. Furthermore, *CBX2* overexpression was associated with poorer survival in the entire TCGA breast cancer cohort of 1084 patients with available survival data (Fig. 4). The survival analysis was applied to all 1084 cases, instead of the 110 cases with tumour-adjacent normal pairs, because we wanted to evaluate the clinical impact of *CBX2* more broadly in the patient population. A significant reduction in 5-year survival in tumours that overexpressed *CBX2* vs. those that did not was observed (*q* = 0.03, log-rank test). This result is consistent with a report that found that high levels of CBX2 protein expression in breast tumours was associated with an increased risk of mortality.^23^ However, no survival differences were detected between patients with tumours that do versus do not overexpress *CBX2* within each the 5 molecular subtypes (*q* > 0.05). These results demonstrate that *CBX2* overexpression is associated with survival outcomes that exist across, but not within, the intrinsic molecular subtypes.

### *CBX2* is expressed at low levels in most adult female tissues

To maximise efficacy and minimise side effects, an ideal drug target needs to be highly expressed in and specific to cancerous tissue, while also expressed at low levels in most other tissues. To examine the expression levels of *CBX2* in normal adult tissues, data from the GTEx portal (https://www.gtexportal.org/home/) was used to examine the expression levels of *CBX2* across 53 normal adult tissues from 8555 individual samples obtained from 544 human donors. *CBX2* was highly expressed specifically in adult testes and expressed at low levels in virtually all other tissues in both men and women (Supplementary Figure 9). Targeted inhibition of *CBX2* may therefore pose a novel therapeutic strategy with minimal side effects on healthy tissue for women whose breast tumours overexpress *CBX2*.

### *CBX2* siRNA knockdown slows the growth of breast cancer cells

**Fig. 5:**
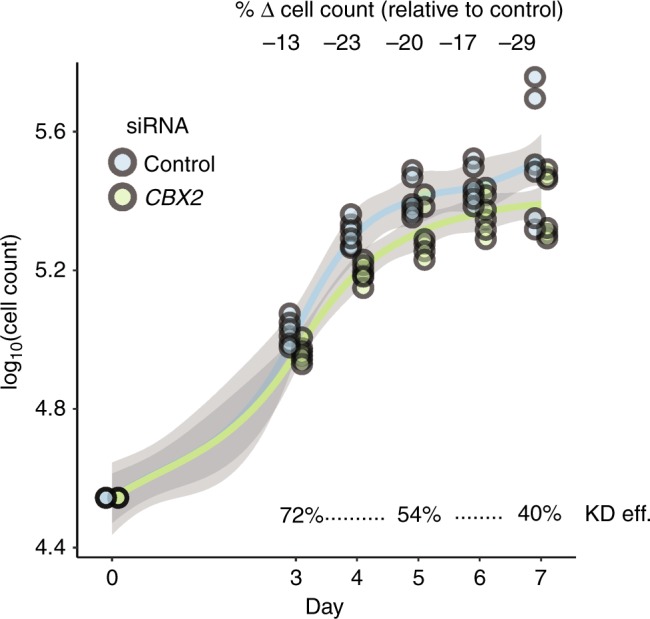
Genetic knockdown of *CBX2* impedes breast cancer cell growth. The cell growth rate for MCF7 breast cancer cells was calculated over a 7-day period following transfection of anti-CBX2 or scrambled siRNA. Adherent (alive) fractions of cells were counted. Each point represents one cell count from one of three biological replicates, each with two technical replicates. The three growth phases are depicted underneath each plot. KD eff. = *CBX2* knockdown efficiency

Although prior associative computational studies suggest that *CBX2* is linked to breast cancer,^22^ no study has experimentally demonstrated a role for *CBX2* in breast carcinogenesis. To investigate the role of *CBX2* in promoting breast cancer growth, we performed genetic knockdown of *CBX2* in MCF7 breast cancer cells. We observed that adherent MCF7 breast cancer cells grew, on average, 20% more slowly over the course of 7 days following *CBX2* siRNA knockdown relative to a scrambled siRNA control (Fig. 5, three-way ANOVA, *P*-value = 7.0 × 10^−7^). These results suggest that *CBX2* is involved in regulating the growth of breast cancer cells and that inhibition of *CBX2* function may serve as a therapeutic strategy to slow the rate of breast cancer cell growth.

## Discussion

Human breast tumours have a broad array of drivers that modulate growth and metastasis. The identification of additional oncogenic drivers will expand our repertoire of personalised therapeutic targets for breast cancer. Here we developed a method, termed oncomix, which identified OC genes with known roles in oncogenesis, and which unveiled subgroups of patients that overexpress the OC. The value of this tool is made clear by considering *CBX2*, the most promising OC identified, and its implications as a potential drug target for breast carcinoma.

*CBX2* is a gene whose protein product binds to H3K9me3 and H3K27me3 sites with high affinity in mice and forms part of the PRC1, a multi-protein complex that modifies histones and preserves stemness by silencing lineage-specifying regulator genes in intestinal and embryonic stem cells.^35–37^ Our results, which are the first to demonstrate that *CBX2* siRNA knockdown slows breast cancer cell growth, build upon previous studies that showed that *CBX2* siRNA knockdown promotes PrCa cell death.^21^ *CBX2* is consistently upregulated in castration-resistant PrCa metastases and its expression correlates with poor patient outcomes in breast and PrCa.^21–23^ Furthermore, we show that breast tumours that overexpress *CBX2* highly express genes that belong to cell cycle-related pathways. This result is consistent with a prior study, which showed that over 500 differentially expressed genes between *CBX2* knockdown and wild-type PrCa cells were enriched in proliferation-related processes.^21^ Our finding is also consistent with the established role of many oncogenes as drivers of transcriptional alterations within signalling pathways that promote cellular growth.^38,39^

Multiple lines of evidence lend support to *CBX2* as a potential drug target against aggressive subtypes of breast carcinoma. First, *CBX2* is expressed at low levels in most healthy adult female tissues and targeted CBX2 inhibition may therefore spare non-tumour tissue and result in fewer side effects. Second, tumours that overexpress *CBX2* are mostly classified as HER2^+^ or basal-like, an aggressive subtype against which there are no specific chemotherapeutic interventions and are associated with poor overall 5-year survival. Third, *CBX2* inhibition via genetic knockdown impedes the growth of breast cancer cells, which suggests that *CBX2* may have an important role regulating breast cancer growth. Fourth, *CBX2* contains a chromodomain that can be pharmacologically targeted and the crystal structure of *CBX2* was recently solved in complex with a PRC1-specific chromodomain inhibitor, Unc3866.^40^ In sum, the results from previous and the current study suggest that *CBX2* is a potential therapeutic drug target in breast cancer.

The identification of a strong association between DNA methylation—a reversible transcriptional regulatory process—and *CBX2* overexpression suggests that *CBX2* expression may be reversibly regulated to drive important tumour behaviour, such as the switch between cell division and metastasis. Prior work suggests a role for *CBX2* overexpression in driving PrCa metastasis that was reversible upon siRNA inhibition of *CBX2*.^21^ Metastatic cancer cells undergo reversible changes during the complex processes of extravasation, infiltration, seeding and proliferation within distant sites, and members of the polycomb complex, such as EZH2, have been associated with metastasis and invasion.^41,42^ This apparent plasticity is likely to be governed by epigenetic processes, as opposed to DNA sequence mutations. This is because molecular and cellular plasticity is required to navigate between the dichotomous processes of cell migration, which occurs as tumour cells metastasize to distant tissues, and cell division, which resumes as metastatic tumour cells seed a new site (as reviewed by Tam and Weinberg^43^). The previously published observation that the *CBX2* locus is rarely mutated in human cancers supports the role of *CBX2* in such processes.^22^ In addition, regulation of *CBX2* expression by DNA CpG methylation may be important for regulating cell division and metastasis, a process that occurs in aggressive breast tumour subtypes (e.g., Basal-like and HER2^+^) and one that requires dynamic reversibility between cell cycling and cell migration during the epithelial to mesenchymal transition.^43^ Future studies will investigate the cause-and-effect relationship between expression and DNA methylation at the *CBX2* locus and its role in promoting breast carcinogenesis.

When comparing the genes identified by oncomix vs. the other two methods, mCOPA and limma, it was clear that the underlying assumptions made by regarding distributions of the data drive the ranking of the genes. The top five candidates identified by mCOPA and limma highlight how these methods are built to identify genes with specific distributions that deviate from the profile detected by oncomix (Supplementary Figure 3). Specifically, limma highly ranks genes where the separation between tumour and normal sample means is maximal. mCOPA is designed for the analysis of microarray experiments, is more appropriate for identifying individual outliers and does not select for genes with visible subsets of patients that overexpress a gene. Oncomix is the only method tested that identifies genes with tumour samples that are grouped into two visible clusters (Fig. 3b) and with low expression in adjacent normal tissue. Of note, other available methods are used to detect genes with bimodal expression but do not allow for comparisons between tumour and normal samples. For example, SIBER (systematic identification of bimodally expressed genes using RNA-seq data)^8^ was developed for single populations of similar samples (e.g., tumour or adjacent normal samples only). Therefore, oncomix is unique in its ability to leverage bimodal changes occurring between tumour and adjacent normal samples to identify OCs.

In summary, we have identified an OC, *CBX2*, based on a model that captures subgroups of tumours that overexpress mRNA relative to normal tissue. Computational and experimental evidence point to the role of *CBX2* as a regulator of breast cancer cell growth. Our computational method, oncomix, is a flexible approach for modelling population-level gene expression data to identify OCs. Conceptually, oncomix may also be adapted to capture tumour suppressor candidates. Although breast cancer, a well-studied form of cancer, was used as a proof-of-concept example for our method, oncomix can detect OCs in additional types of cancer (Supplementary Figures 10–12).

*CBX2* may serve as a potential therapeutic strategy against aggressive breast cancers, due to its low expression in healthy female tissues, available pharmacologic inhibitors and association with poor survival. Future experimental studies are required to address how DNA methylation within the *CBX2* locus is associated with oncogenic processes such as cell division within both bulk tumour tissue as well as single tumour cells. The role of *CBX2* in other solid cancers should also be investigated, as a similar overexpression profile for *CBX2* is observed in endometrial and lung adenocarcinoma (Supplementary Figure 13). Our novel approach to identifying OCs through oncomix will be particularly useful for identifying regulators of previously unknown tumour subgroups within cancer datasets that include expression levels from hundreds or thousands of patient tumours and their adjacent normal tissue.

## Supplementary information

Supplementary Information

Code Associated with all Figures and Tables Generated in the Manuscript

## Data Availability

All analysis was performed in the statistical language R (version 3.4.3). An HTML document created using knitR and RMarkdown contains the code and workflow for all analysis performed in this study (Supplementary File 2). An R package “oncomix” for identifying oncogene candidates in large cohorts of RNA-sequencing data from tumour and adjacent normal samples is available through Bioconductor (https://bioconductor.org/packages/release/bioc/html/oncomix.html).^44^ The oncomix package is platform independent, requires R version 3.4.3 or higher, and is released under a GPL-3 license.
