# Supplementary material for: A novel approach to modelling transcriptional heterogeneity identifies the oncogene candidate CBX2 in invasive breast carcinoma
Source: Br J Cancer. 2019 Mar 1;120(7):746–53. doi: 10.1038/s41416-019-0387-8 (PMC6462018; doi:10.1038/s41416-019-0387-8)
Supplement: Supplementary file 1 — Supplementary Information [file 41416_2019_387_MOESM1_ESM.pdf]

Supplementary information for "A novel approach to modeling transcriptional heterogeneity identifies the oncogene candidate *CBX2* in invasive breast carcinoma"

Supplementary Methods

Estimation of mixture model parameters

To investigate whether certain genes expressed in tumors exhibited distinct, clearly separable clusters of gene expression values, a 2-component Gaussian mixture model was fit to each gene across the 110 data points. These mixture models were applied separately for gene expression values from both tumors and adjacent normal samples. For each gene within each group (either tumor or adjacent normal), 4 parameters – namely, the mean of the Gaussian with the lower (μ_L) and higher (μ_H) mean, the proportion of samples under the Gaussian with the smaller of the two means (π), and a common standard deviation (σ) – were estimated using maximum likelihood through the well-established method of expectation maximization[1] (**Figure 1B**). The variance of the mixture model was set to be equal between the two Gaussians to stabilize the expectation maximization procedure. Each parameter includes an additional letter subscript ("T" or "N") to denote whether the parameter refers to the model describing the tumor (T) or adjacent normal (N) expression data.

Selection and filtration of genes

To remove genes with extreme outliers and to allow for sufficient statistical power for downstream analysis, genes with a proportion of low-expression modal membership between 0.2 > π_T & π_N > 0.8 were selected. Additional filtering of genes was performed as described in **Figure 1B**. To identify and rank genes whose expression values defined a distinct subgroup of

tumors that overexpressed the gene relative to normal tissue, two statistics was derived from the mixture model parameters. The first, termed the selectivity index (*SI*), was used to screen candidate genes with an overexpressed subgroup of tumors and was defined as follows:

$$SI = \frac{1}{n}\sum_{i=1}^{n}\begin{cases} 1, if\ x_i < \frac{\mu_{LT} + \mu_{HT}}{2} \\ 0, otherwise \end{cases} \qquad \textbf{(Equation 1)}$$

where n is the number of paired samples with gene expression values (here, $n = 110$), x_i is the $\log_2$(TPM+1) expression value of the ith adjacent normal sample, and $\frac{\mu_{LT} + \mu_{HT}}{2}$ is the boundary, or point of equal probability, between the low and high expression modes of the Gaussians that describe the tumor data. The SI is applied separately to each gene and ranges between 0 and 1, with values closer to 1 indicative of genes that have a subpopulation of samples that are clearly distinct and separable based on the expression values from tumors for a given gene. The SI is unique in that it selects genes that define distinct clusters of tumor samples based on expression values that are separate from and greater than their adjacent normal counterparts as well as from other tumor samples. After visually inspecting the distribution of SI values for all genes (**Figure 1A**) a conservative SI cutoff of 0.99 was selected.

The second statistic that was developed was termed the oncomix score. The oncomix score is calculated as a function of the SI (see Equation 1) and the $\Delta\mu_H, \Delta\mu_L, \sigma_N, \sigma_T$ parameters, as shown below:

$$Oncomix\ Score = SI * \{(\Delta\mu_H - \Delta\mu_L) - (\sigma_N + \sigma_T)\}\ , \qquad \textbf{(Equation 2)}$$

where $\Delta\mu_H = \mu_{HT} - \mu_{HN}$ and is the difference between the means of the high expression groups of the mRNA values from tumor (μ_{HT}) and adjacent normal tissue (μ_{HN}). This term, when large, indicates greater separation between the high expression modes of the tumor and adjacent normal populations and would contribute to a larger and more favorable oncomix score. The difference

between the low expression groups of the tumor (μ_{LT}) and adjacent normal samples (μ_{LN}) was calculated as $\Delta\mu_L$ (μ_{LT} - μ_{LN}). This term, when small, indicates a minimal difference between the low expression modes of the tumor and adjacent normal populations and results in a larger oncomix score. The oncomix score is penalized by the variance of each mixture model (σ_N & σ_T), with larger variances resulting in lower scores. This is because mixture models with large variances reflect an underlying spread in the distribution and provide evidence against the existence of two distinct clusters of tumor expression data, and of a single cluster of normal tissue data.

Identification of a subset of existing oncogenes that are overexpressed in a subset of tumors

While oncomix was primarily intended to discover novel oncogenes, it was also imperative to evaluate whether our method could recover any well-established oncogenes. To do this, all Tier 1 oncogenes were used from the Cancer Gene Census (CGC) database (196 genes)[2,3], a collection of genes with mutations that are causally associated with cancer derived from all tumor types. Of the 196 Tier 1 oncogenes from the CGC, twelve genes (6.1%) had an SI > 0.99 and an oncomix score > 0 (**Supplementary Figure 1**). The gene expression distributions of these twelve genes in the matched tumor-normal samples from the TCGA breast cancer patients showed that most of these distributions contained a subset of tumors that overexpressed the given gene relative to normal tissue (**Supplementary Figure 1**). Of these twelve genes, five (*HOXA13*, *TAL2*, *SOX2*, *HOXD13,* and *SALL4*) are transcription factors that help govern embryonic mammalian development and are transcriptionally silent in most adult tissues[4-7] (**Supplementary Figure 14**). We conclude that our approach successfully identified a small subset of known oncogenes whose function may be mediated through gene overexpression.

Power analysis

Oncomix provides a way for users to rank oncogene candidates within a cancer dataset based on patterns of gene expression between tumor and adjacent normal tissue. Because oncomix is not based on hypothesis testing, deriving exact power calculations for this approach is non-standard and difficult. To work around this, we conducted a simulation study to estimate the power of the oncomix approach based on the design parameters used in our study. Here, the null hypothesis is defined as there being no significant difference in the oncomix score of the top 5 ranked oncogene candidates relative to the rest of the 134 genes that passed the initial filters ($0.2 > \pi_T$ & $\pi_N > 0.8$, selectivity index > 0.99). Power is defined as the probability of rejecting the null hypothesis when the null hypothesis is incorrect. Therefore, the alternative hypothesis is that the oncomix scores of the top 5 ranked oncogene candidates are significantly higher than those genes not ranking in the top 5.

Oncomix scores were simulated by assuming that 4 main parameters (SI, $\Delta\mu_H$, $\Delta\mu_L$, σ_N, σ_T) comprising the oncomix score from the two groups (top 5 genes versus bottom 134 genes) were drawn from two separate multivariate Gaussian distributions. A 5th parameter, the SI, was simulated using a bootstrap approach due to the narrow support and non-Gaussianity of this parameter. Parameters for these distributions were estimated from the observed data and were fit using the mvrnorm function in the MASS library in R[8]. With a sample size of 110 adjacent normal and tumor samples, and at an alpha level of 1.91×10^{-6} (student's 1-sided t-test), the power to correctly reject the null hypothesis is 0.723 (out of 1000 simulations) (**Supplementary Figure 2**).

The oncogene candidates identified by oncomix represent a unique set of genes that are not reliably detectable by existing approaches.

For an oncogene candidate to be detected by oncomix, a gene must exhibit a specific expression profile that demonstrates overexpression in a subgroup of cancer patients (**Figure 1B)**. To test whether genes identified by oncomix could be identified by existing approaches, we compared our results with those obtained by two other methods to find potential tumor regulators. Limma is a widely-used method to identify differentially-expressed (DE) genes through a regularized Student's two sample t-test and assumes the presence of a single mode of expression. None of the genes identified by oncomix fell within the top 2% of genes ranked by limma (**Supplementary Table 1** and Methods). In addition, benchmarking was performed against mCOPA, a method that ranks a subset of genes based on meeting a fold change threshold between pre-specified percentiles from expression profiles in tumor and normal samples[7]. mCOPA ranked only one out of our five identified OCs, even after pre-specifying three different percentiles (see Methods). The genes that were highly ranked by these methods are shown in **Supplementary Figure 3** (compare with **Figure 2B**). We conclude that our method detects unique genes with established roles in oncogenesis and metastasis for a subset of patients, and that these genes are not detectable using existing DE methods that compare tumor and adjacent normal samples.

Supplemental molecular and clinical datasets

All supplemental data were downloaded from GDC servers using the GenomicDataCommons and TCGAbiolinks R packages (see **Supplementary File 2**, section "Summary of Data sources" for details on downloaded files). 75% (82/110) of tumor samples in this study also had DNA methylation data processed on Illumina 450k arrays that was obtained from the same tumor. The FDb.InfiniumMethylation.hg19 R package was used to obtain 450k

CpG coordinates for hg19, which were mapped to hg38 using the rtracklayer R package[9,10]. DNA CpG methylation loci beta values were obtained from Illumina 450k arrays (see **Supplementary Figure 4**). For the logistic regression analysis, only those CpG methylation loci from the TSS1500 to the 3' UTR within each respective oncogene candidate were used. The TxDb.Hsapiens.UCSC.hg38.knownGene R package was used to obtain the genomic coordinates for each oncogene candidate[11]. Log_2 mean segment copy number values for CNV obtained from an Affymetrix 6.0 SNP array were utilized. Clinical data was numerically codified or scaled to within a range of 0-1, and the molecular subtype was inferred from the log_2(TPM+1) mRNA expression data from each tumor using the AIMS algorithm[12].

All 66 transcription factor and histone ChIP-seq data from MCF7 cells with 2 biological or technical replicates was downloaded from ENCODE servers using the 'rutils' tool in April 2017. All downloaded data was aligned to hg38, and peaks were called using standard ENCODE processing pipelines[13,14]. Of the 66 ENCODE data sets, 14 (three transcription factors and 11 histones) overlapped with at least one CpG site within the *CBX2* locus. From these 14 ChIP-seq data sets, seven ChIP-seq experiments were manually selected based on their established association with transcriptional regulation[14].

**Gene symbol**	**Function (NCBI gene summary)**	**Chromo-some**	Oncomix **score/ Rank**	**Limma Rank (out of 7,388 upregulated genes)**	**mCOPA Rank (out of 2,152 ranked genes)**
EPYC	Member of the small leucine-rich repeat proteoglycan family	12q21.33	1.84 / 1	279	NA
NELL2	Neural epidermal growth factor-like like protein 2	12q12	1.64 / 2	2264	NA
CBX2	Member of polycomb repressive complex	17q25.3	1.48 / 3	756	NA
SLC24A2	Member of calcium/cation antiporter superfamily of transport proteins	9p22.1-p21.3	1.40 / 4	149	NA
LAG3	Lymphocyte-activation protein 3	12p13.31	1.28 / 5	3077	1076

Supplementary Table 1. List of oncogene candidate function and comparison with current differential expression approaches. Each oncogene candidate is represented by a row. Columns indicate the molecular features or function of each gene. A rank-based comparison between the oncomix score, limma's p-value, and mCOPA's fold change is shown. Genes with a selectivity index > 0.99 were ranked according to the oncomix score. A limma rank of 1 is assigned to the gene that was most differentially expressed (ie has the lowest p-value) between tumors and adjacent normal samples, and a limma rank of 7,388 is the lowest possible rank and indicates the gene that was least differentially upregulated in tumors relative to normal tissue. mCOPA identified 2,152 genes that contained overexpressed outliers after selecting genes that had at least a $\log_2$(fold change) > 2 between tumor and normal samples at the 70th, 80th, or 90th percentile. Genes were ranked according to $\log_2$(fold change). NA indicates that the gene was not selected by mCOPA.

Oncogene Candidate	Upregulated genes	Downregulated genes
EPYC	4	0
NELL2	0	0
CBX2	73	17
SLC24A2	241	1
LAG3	105	2

Supplementary Table 2. Summary of differentially expressed genes in breast tumors that overexpress oncogene candidate mRNA Each oncogene candidate is represented as a row. The number of upregulated and downregulated genes are relative to tumors that overexpress the oncogene candidate. Differential expression was performed using limma with $\log_2$(Fold Change) > 1 & q-value < 0.0001 as cutoffs.

**Oncogene Candidate**	**Geneset**	**q value**	**Odds Ratio**	**Odds Ratio 95% CI**
CBX2	**hallmark g2m checkpoint**	2.20E-30	54	31-91
CBX2	hallmark e2f targets	1.30E-25	44	25-75
SLC24A2	hallmark epithelial mesenchymal transition	1.30E-59	37	26-53

Supplementary Table 3. Gene set enrichment from upregulated genes in breast tumors that overexpress a given OC. Two OCs had significant enriched pathways following gene set enrichment performed using Fisher's exact test. Pathways are shown as rows. Pathways that have an odds ratio with a lower bound 95% CI > 20 and a Benjamini-Hochberg adjusted q-value < $1x10^{-20}$ are shown and are ranked, from top to bottom, by decreasing odds ratio within each OC.

HGNC symbol	Description	log2(Fold Change)	q value	Chromosome
KIF2C	kinesin family member 2C	1.55	1.30E-06	1p34.1
RAD54L	RAD54 like	1.26	5.80E-06	1p34.1
CDC20	cell division cycle 20	1.63	9.30E-06	1p34.2
E2F2	E2F transcription factor 2	1.14	9.14E-05	1p36.12
EXO1	exonuclease 1	1.3	6.97E-05	1q43
CENPA	centromere protein A	1.59	7.00E-07	2p23.3
BUB1	BUB1 mitotic checkpoint serine/threonine kinase	1.35	6.30E-06	2q13
CENPE	centromere protein E	1.09	6.48E-05	4q24
CCNA2	cyclin A2	1.29	5.55E-05	4q27
MAD2L1	mitotic arrest deficient 2 like 1	1	8.91E-05	4q27
TTK	TTK protein kinase	1.29	1.06E-05	6q14.1
EZH2	enhancer of zeste 2 polycomb repressive complex 2 subunit	1.01	1.26E-05	7q36.1
CDK1	cyclin dependent kinase 1	1.17	7.80E-05	10q21.2
TROAP	trophinin associated protein	1.35	1.07E-05	12q13.12
ESPL1	extra spindle pole bodies like 1, separase	1.17	3.66E-05	12q13.13
PLK1	polo like kinase 1	1.42	1.37E-05	16p12.2
ORC6	origin recognition complex subunit 6	1.08	2.86E-05	16q11.2
SLC7A5	solute carrier family 7 member 5	1.63	6.07E-05	16q24.2
BIRC5	baculoviral IAP repeat containing 5	1.65	1.30E-06	17q25.3
NDC80	NDC80, kinetochore complex component	1.18	5.77E-05	18p11.32
CDC25B	cell division cycle 25B	1.14	2.86E-05	20p13
TPX2	TPX2, microtubule nucleation factor	1.44	1.14E-05	20q11.21
E2F1	E2F transcription factor 1	1.27	3.27E-05	20q11.22
MYBL2	MYB proto-oncogene like 2	2.06	1.30E-06	20q13.12
UBE2C	ubiquitin conjugating enzyme E2 C	1.64	1.58E-05	20q13.12
AURKA	aurora kinase A	1.42	3.10E-06	20q13.2
CDC45	cell division cycle 45	1.25	4.00E-05	22q11.21

Supplementary Table 4. Significantly differentially expressed and upregulated genes within the Hallmark G2/M checkpoint pathway for tumors that overexpress *CBX2*. Each gene is listed as a row, and a description is provided for each gene from the Hugo Gene Nomenclature Committee (HGNC), along with the log_2(Fold Change), Benjamini-Hochberg adjusted q value, and chromosomal location. The genes are listed from top to bottom in order of chromosomal location. All genes listed have a log_2(Fold Change) > 1 & q-value < 0.0001.

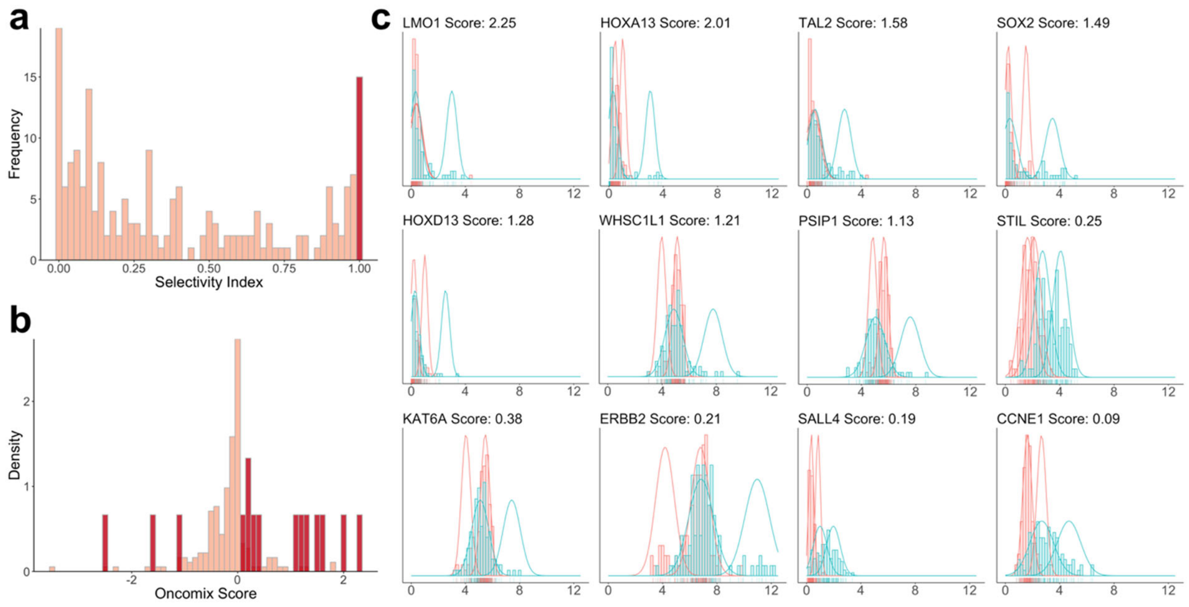

Supplementary Figure 1. Oncogenes from the Cancer Gene Census can be detected using oncomix. A) The distribution of selectivity indices across 196 oncogenes from the CGC is shown. B) Distribution of oncomix scores for the same 196 oncogenes separated by their selectivity index. Dark red bars indicate the genes that have a selectivity index greater than 0.99 (N=15). C) Superimposed histograms of expression values from tumor (teal) and adjacent normal (red) samples for the 12 oncogenes with oncomix score greater than 0 and a selectivity index greater than 0.99. The best fitting mixture model is shown for each selected gene. The HUGO gene symbol for each gene is displayed for each histogram. The y-axis represents density and the x-axis represents $\log_2$(TPM + 1) reads. Tumor samples are shown in teal, and adjacent normal breast tissue is shown in orange. Abbreviations: TPM = Transcripts Per Million reads.

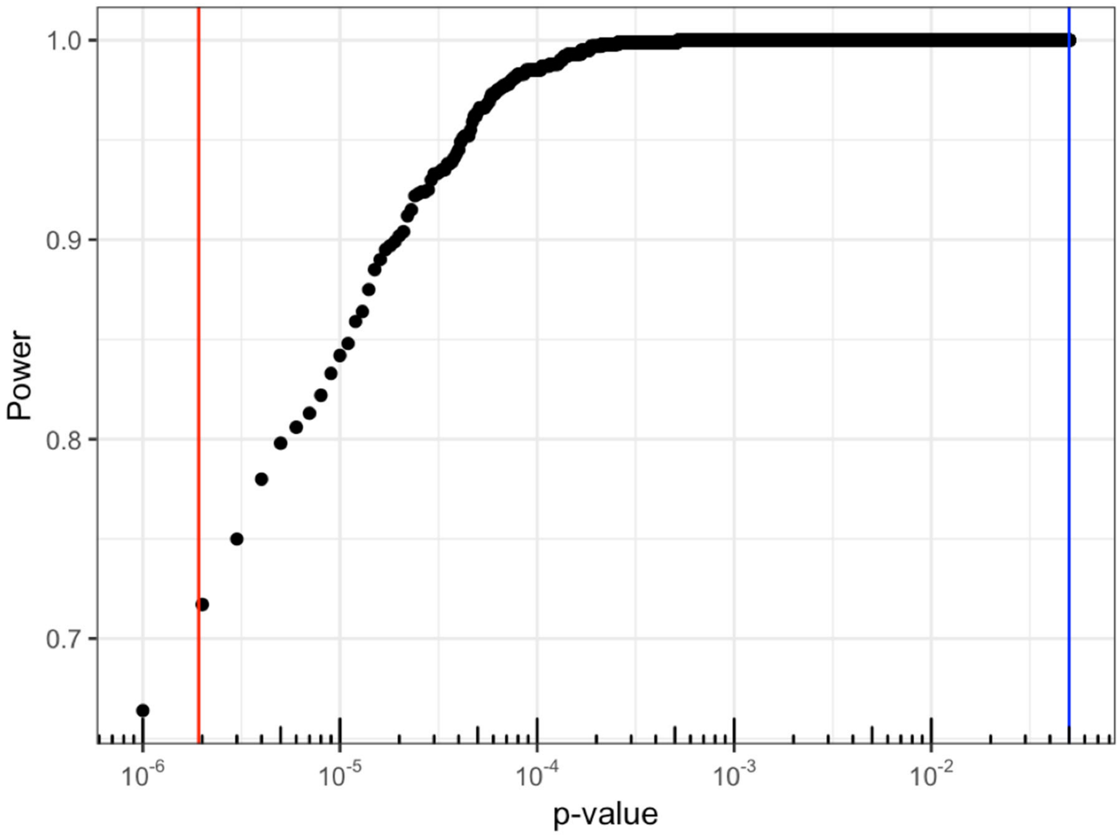

Supplementary Figure 2. Power analysis based on simulations of observed oncomix parameter values. The x-axis shows the p-value (log_{10} scale), and the y-axis represents the power. Each black point represents the power along a grid of p-values between $1x10^{-6}$ and 0.05 with each step of size $1x10^{-6}$. The vertical red line represents the observed p-value ($1.91x10^{-6}$ (Student's 1-sided t-test) in this study, and the blue line represents a p-value of 0.05.

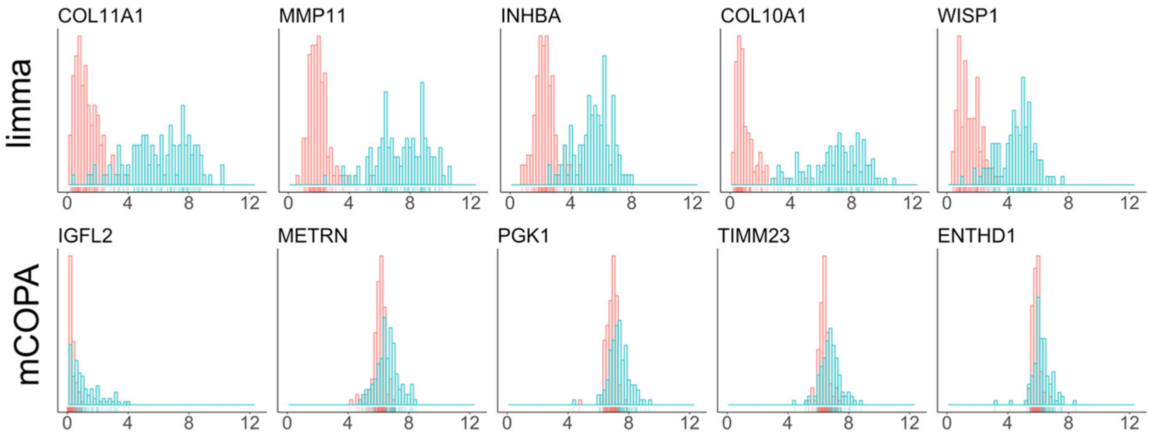

Supplementary Figure 3. Comparison of the distributions from the 5 top genes (out of 16,156) identified from 2 different types of differential expression approaches. The distributions of log_2-transformed transcripts per million reads for 110 tumor (teal) and adjacent normal (red) samples are shown along the x-axis. The y-axis represents density. (Top Row) Differential expression analysis between tumor and adjacent normal samples using limma, a technique that performs a two-sample t-test. The top 5 genes with the lowest p-value among 16,156 genes are shown, and genes are shown from left to right by progressively increasing p-value. (Bottom row) The top 5 genes derived from mCOPA analysis of tumor and adjacent normal samples with the highest log_2 fold change between the 80th percentile for tumor and adjacent normal samples are shown. Log_2 fold change was calculated based on COPA-transformed expression values, which are not shown here.

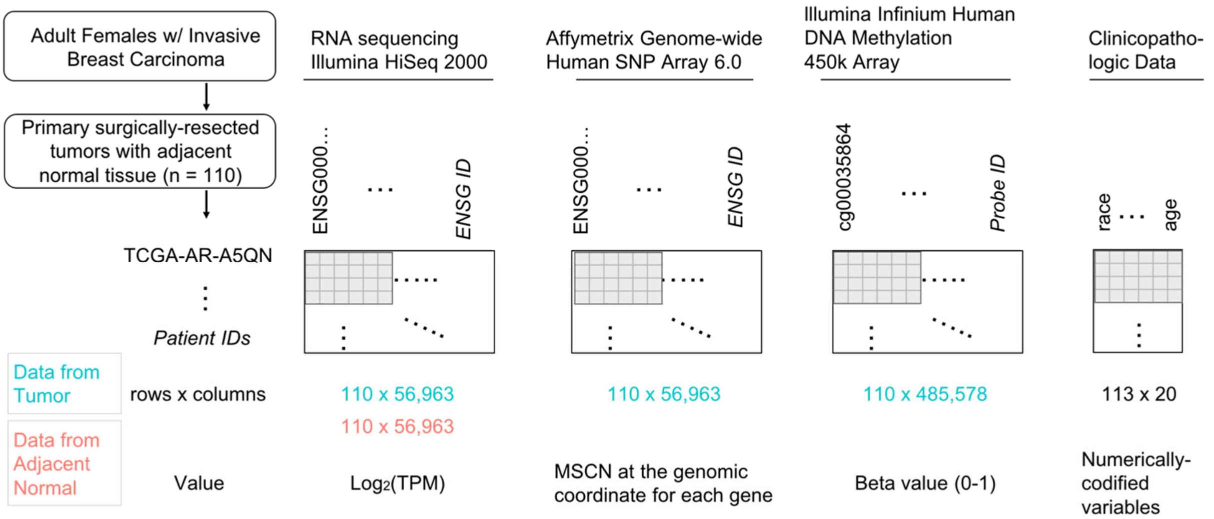

Supplementary Figure 4. Overview of study design and schematic of molecular and clinicopathologic data matrix organization. All data were downloaded from the Genomic Data Commons/Cancer Genome Atlas (TCGA) and were organized into distinct matrices based on the type of data (RNA sequencing, DNA methylation, genomic copy number, and clinicopathologic information). Representative examples of TCGA patient IDs (rows) and of the 4 distinct data types (columns) are shown. Patients were selected using the following 2 criteria: 1) no prior chemotherapeutic treatment for invasive breast carcinoma, and 2) the presence of RNA sequencing data from both tumor and adjacent normal tissue. The values of the entries for each of the 4 matrices are shown below each respective matrix, along with the dimension of each matrix. Individual probes or genes were filtered according to the criteria indicated in the 'filter' row. Code for organization of data matrices is available on Github. Where appropriate, matching annotation files (not shown) were created using UCSC genome annotations (hg38) for transcription start and end sites, DNA methylation loci, and SNP locations. Abbreviations: MSCN = Mean segment copy number; TPM = Transcripts per Million mapped reads.

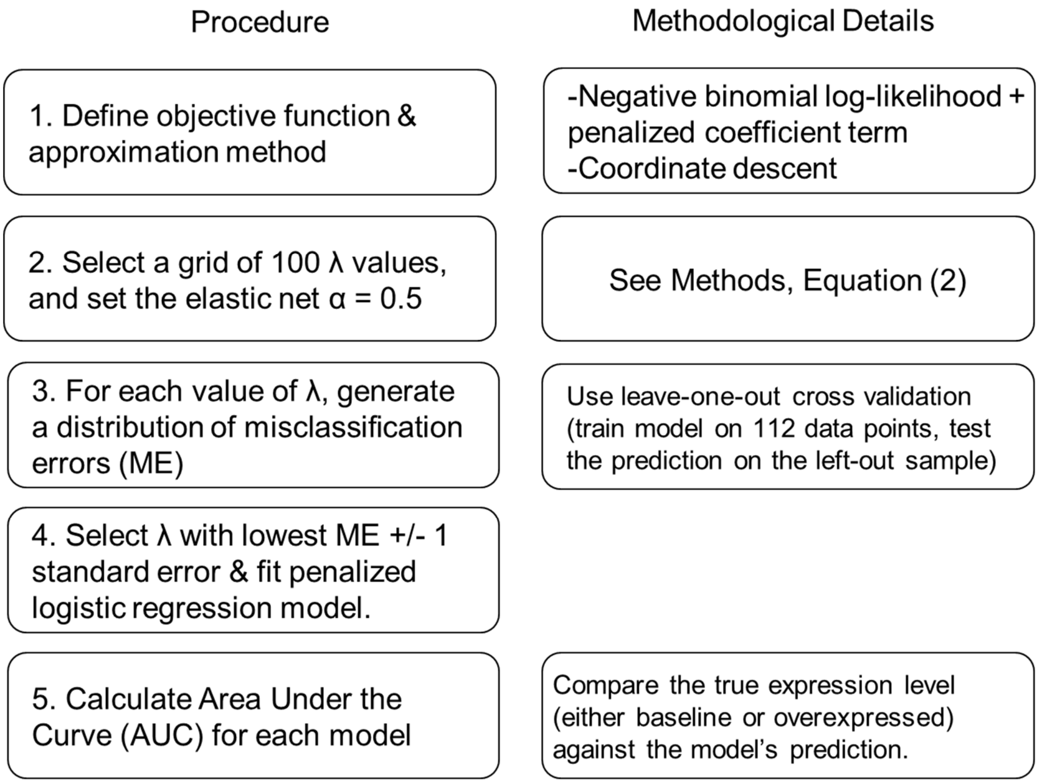

Supplementary Figure 5. Procedure for fitting a multiple logistic regression model via coefficient-penalized maximum likelihood estimation. The procedure with cross validation was implemented using the R package glmnet. Area under the curve (AUC) was implemented using the AUC package. Implementation details are available in Supplementary file 2. (1) The first step is to define the objective function – in this case, the negative binomial log-likelihood – and to define an approximation/optimization method – in this case, coordinate descent. (2-4) Next, the value of lambda, a term that penalizes model coefficients, is selected by training an array of models across a grid of lambda values and selecting the model with the fewest number of terms within 1 standard error of the model with the lowest misclassification error using leave-one-out cross validation. (5) The Area Under the Curve (AUC) is calculated for each model by testing the ability of each model to correctly predict the outcome (either baseline or overexpressed) given a set of input variables (e.g. DNA methylation β values at intragenic CpG loci).

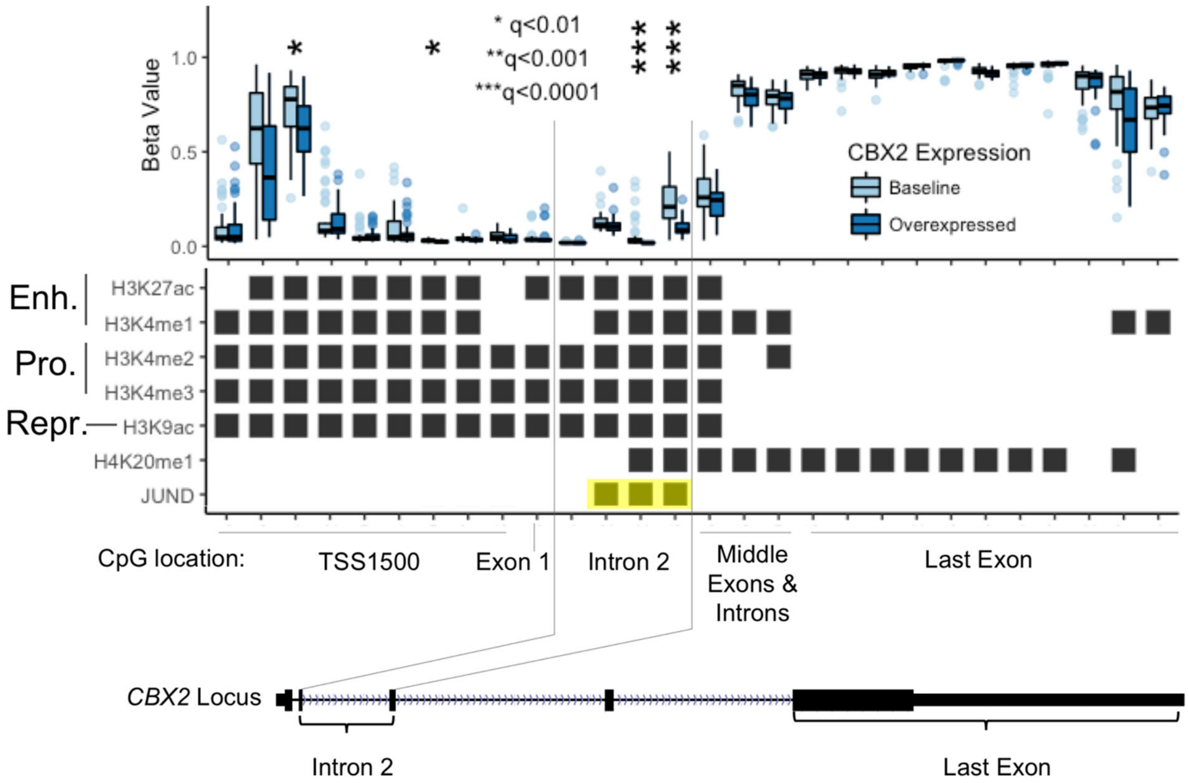

Supplementary Figure 6. Colocalization of histones and transcription factors with CpG sites that predict overexpression of *CBX2*. (Top) Paired boxplots showing the CpG methylation beta values, which range between 0-1, at each of 28 individual CpG loci for tumors that express baseline levels of or overexpress *CBX2*. (Middle) Each row of the black-and-white matrix represents 1 of 7 different ChIP-seq experiments from MCF7 cells in which a direct overlap (black squares) between a CpG site and a ChIP-seq peak was identified. These 7 ChIP-seq experiments were manually selected for purposes of interpretability from 14 ChIP-seq experiments that overlapped with the *CBX2* locus. The chromatin type or transcription factor is listed along the left-hand side of the matrix, and major chromatin features, such as enhancers (Enh.), promoters (Pro.), and repressive (Repr.) marks, are indicated in large text. Each of the 28 columns represents a different CpG locus within the gene body of the *CBX2* gene (defined as the beginning of the TSS1500 to the end of the 3’ UTR). The model coefficient with the largest absolute value is shown adjacent to the rightmost thin black line. (Bottom) The two thin black lines demarcate the position of the 4 CpG sites within intron 2 and indicate the physical position of these intronic CpG sites within the *CBX2* locus. Additional regions within the *CBX2* gene (length = 11,352 bases, including the TSS1500) are annotated in the gene model, which was obtained from the UCSC genome browser. Asterisks represent q values from a Wilcoxon rank-sum test between the beta values at each of the 28 loci. *** = q < 0.0001, ** = q < 0.001, * = q < 0.01.

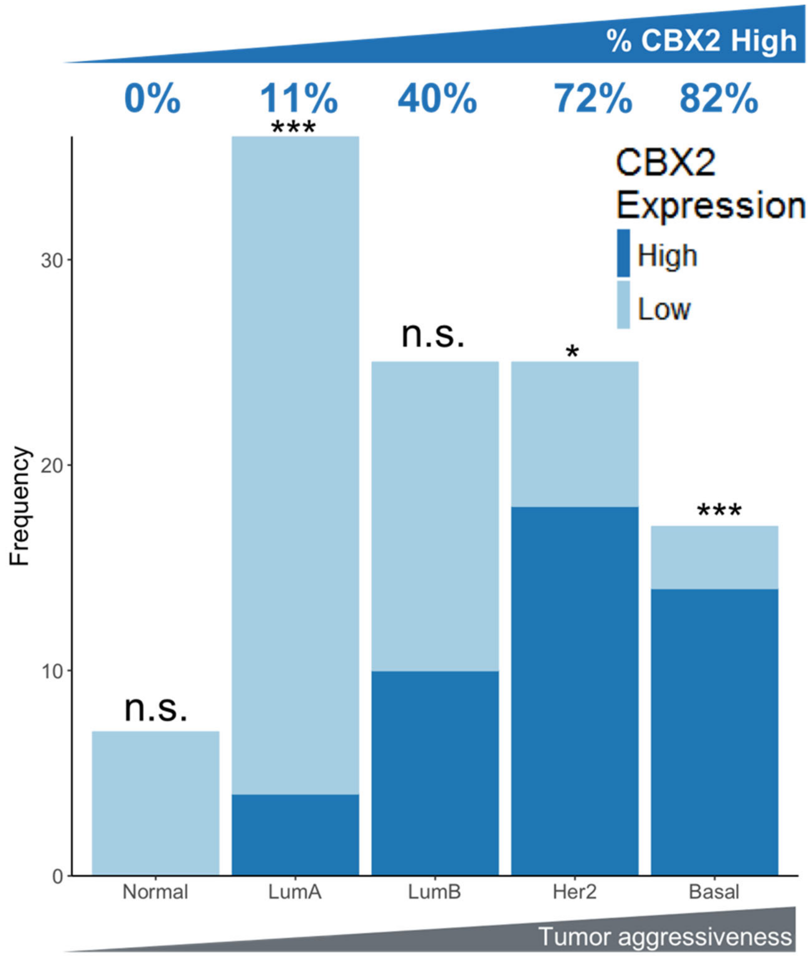

Supplementary Figure 7. Expression of *CBX2* across the 5 distinct subtypes of breast carcinoma. The 110 tumors used in this study were grouped into 5 molecular subtypes, inferred using the AIMS algorithm on the gene expression data derived from each tumor, and are shown along the x axis. The subtypes are ordered from least to most aggressive, moving from left to right. The proportion of tumors that overexpressed *CBX2* within each subtype are shown in dark blue, while tumors that express baseline levels of *CBX2* are shown in light blue. The proportion of tumors that overexpress *CBX2* correlates with the aggressiveness of the tumor subtype. A two-sided multinomial exact test was used to check for the enrichment of tumors that overexpress *CBX2* within the five breast tumor subtypes ($p = 1.149 \times 10^{-7}$). *Post hoc* statistics were calculated using Fisher's exact test (results shown as asterisks above each bar) and were adjusted for multiple comparisons using the Benjamini-Hochberg method. Abbreviations: n.s. = not significant, * = $p < 0.05$, *** = $p < 0.001$.

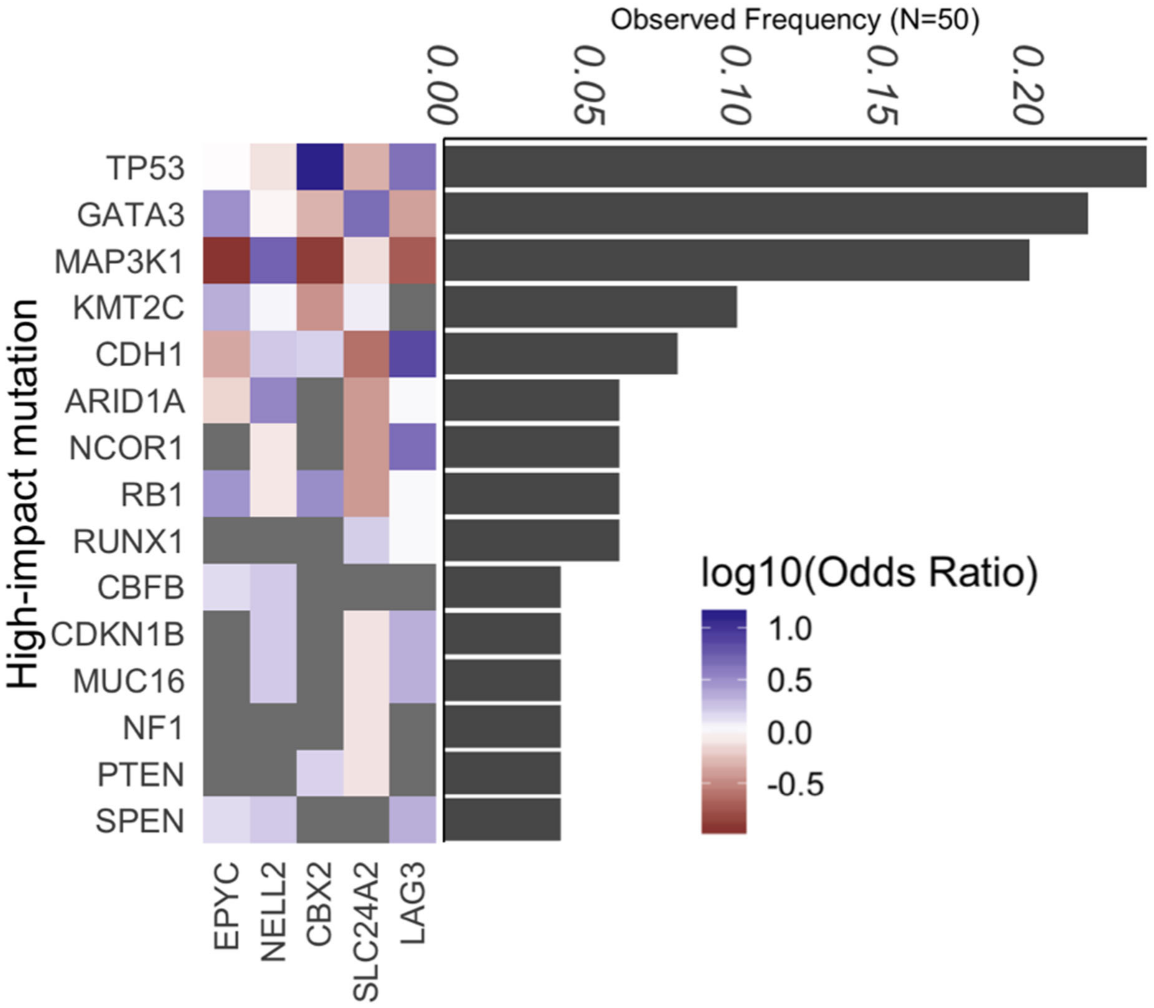

Supplementary Figure 8. Association between high-impact cancer-associated mutations and the overexpression of oncogene candidates. Each column represents one of 5 oncogene candidates, and each row represents a mutation in a known oncogene or tumor suppressor. Fisher's exact test was performed for each relationship, and an odds ratio and p-value were obtained when possible. Blue indicates that overexpression of the OC and the presence of a mutation were likely to co-occur in the same individual, while red indicates that overexpression of the OC and oncogenic mutations were mutually exclusive. The frequency of these mutations in the 50 individuals who harbored them are shown as a bar graph. Dark grey boxes indicate the inability to compute an odds ratio due to the presence of a 0 value in an element of the 2x2 contingency table.

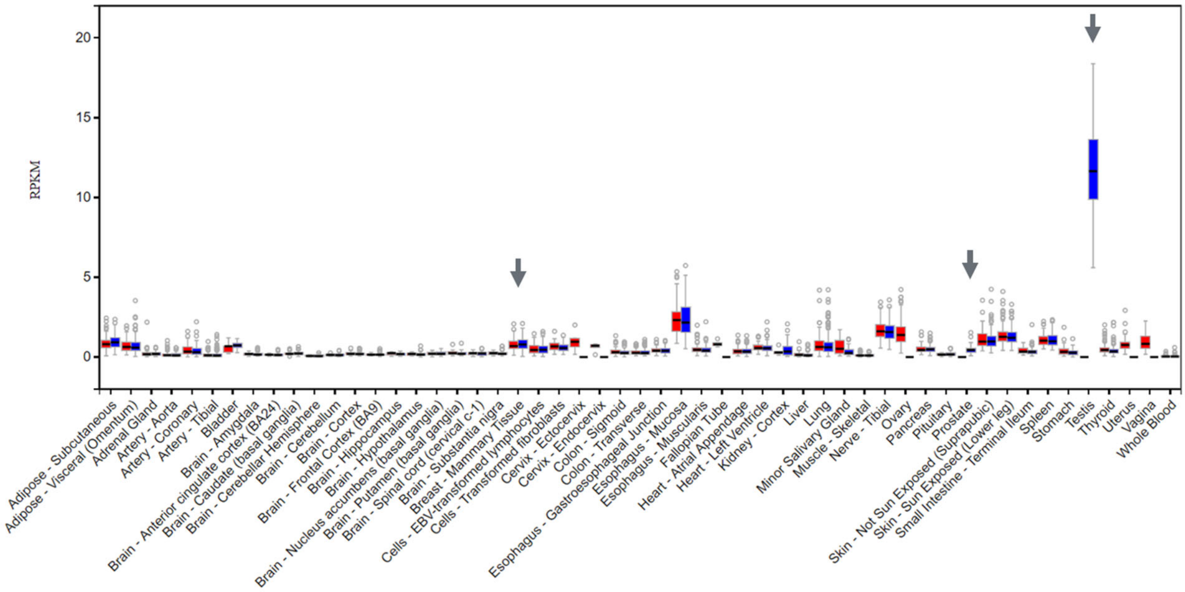

Supplementary Figure 9. Expression of *CBX2* across 53 healthy adult human tissues. Figure was generated from the GTEx website (https://www.gtexportal.org/home/) by searching for the gene *CBX2*. Grey arrows, from left to right, indicate expression in mammary tissue, prostate, and testes. Blue boxplots represent expression values from males, and red boxplots represent expression values from females. The entire GTEx dataset of 53 tissues, shown here for *CBX2*, includes expression values generated from 8,555 individual samples, which were obtained from 544 donors.

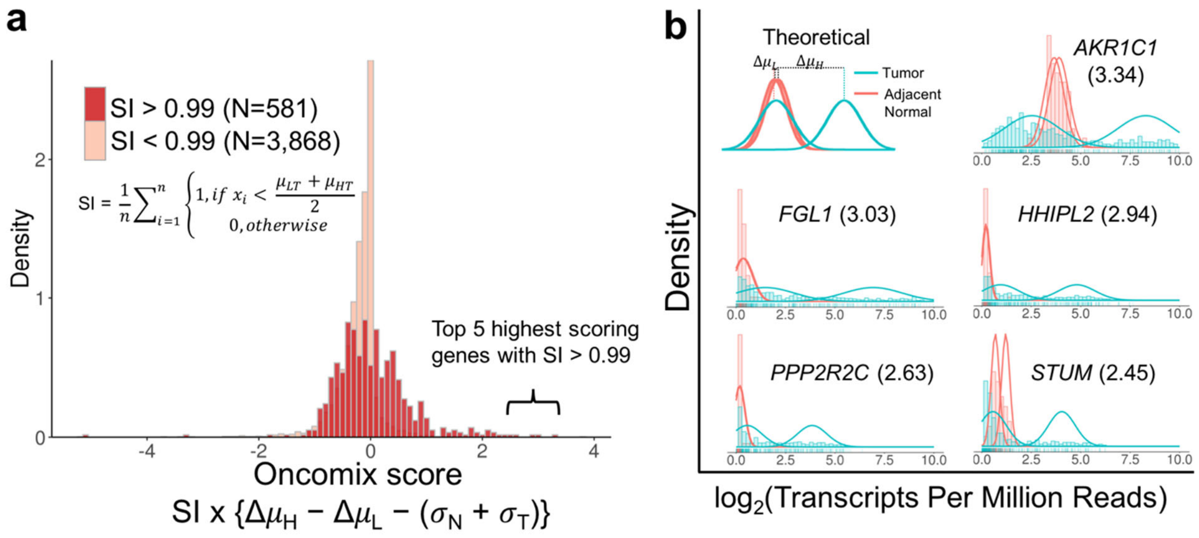

Supplementary Figure 10. The top five oncogene candidates identified by oncomix using RNA-sequencing data from lung adenocarcinoma and adjacent normal lung tissue. A) The distribution of the oncomix scores is colored by a selectivity index (SI) set at 0.99. Larger oncomix scores correspond to genes that more closely resemble the profile of a theoretical oncogene candidate. B) Superimposed histograms of expression values from tumor (teal) and adjacent normal (red) samples for the 5 genes with the highest oncomix score and a selectivity index greater than 0.99. The best fitting mixture model is shown for each selected gene. The HUGO gene symbol for each gene is displayed for each histogram. A theoretical model for an ideal oncogene candidate is shown in the upper left and includes some of the summary statistics that were used to compute the oncomix score. The y-axis represents density and the x-axis represents log_2(TPM + 1) reads. Abbreviations: T = primary breast tumor, N = adjacent normal breast tissue, TPM = Transcripts Per Million reads.

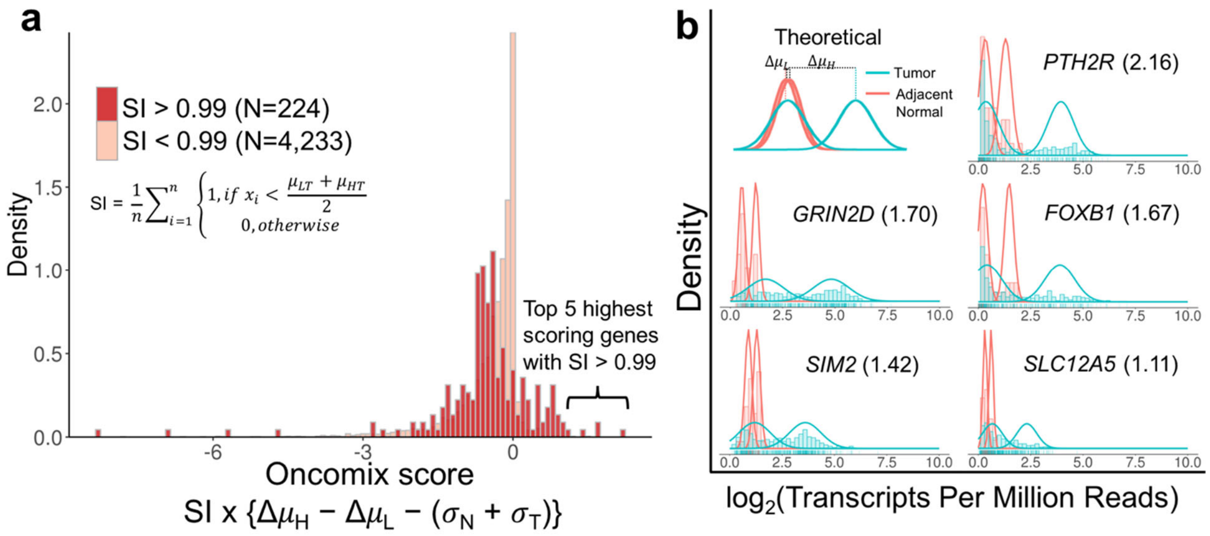

Supplementary Figure 11. The top five oncogene candidates identified by oncomix using RNA-sequencing data from endometrial carcinoma and adjacent normal endometrial tissue. A) The distribution of the oncomix scores is colored by a selectivity index (SI) set at 0.99. Larger oncomix scores correspond to genes that more closely resemble the profile of a theoretical oncogene candidate. B) Superimposed histograms of expression values from tumor (teal) and adjacent normal (red) samples for the 5 genes with the highest oncomix score and a selectivity index greater than 0.99. The best fitting mixture model is shown for each selected gene. The HUGO gene symbol for each gene is displayed for each histogram. A theoretical model for an ideal oncogene candidate is shown in the upper left and includes some of the summary statistics that were used to compute the oncomix score. The y-axis represents density and the x-axis represents log_2(TPM + 1) reads. Abbreviations: T = primary breast tumor, N = adjacent normal breast tissue, TPM = Transcripts Per Million reads.

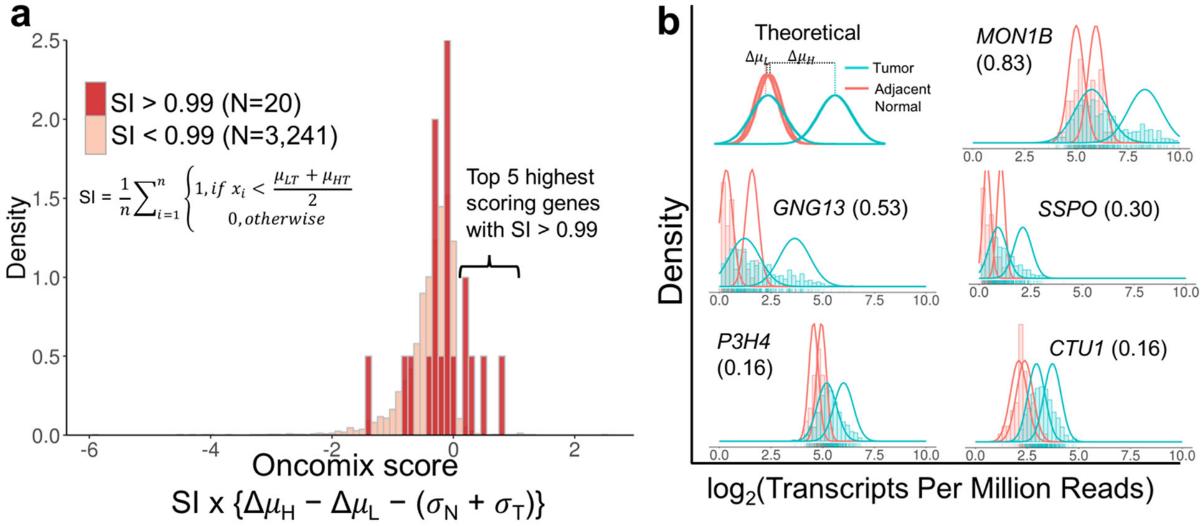

Supplementary Figure 12. The top five oncogene candidates identified by oncomix using RNA-sequencing data from prostate adenocarcinoma and adjacent normal prostate tissue. A) The distribution of the oncomix scores is colored by a selectivity index (SI) set at 0.99. Larger oncomix scores correspond to genes that more closely resemble the profile of a theoretical oncogene candidate. B) Superimposed histograms of expression values from tumor (teal) and adjacent normal (red) samples for the 5 genes with the highest oncomix score and a selectivity index greater than 0.99. The best fitting mixture model is shown for each selected gene. The HUGO gene symbol for each gene is displayed for each histogram. A theoretical model for an ideal oncogene candidate is shown in the upper left and includes some of the summary statistics that were used to compute the oncomix score. The y-axis represents density and the x-axis represents $\log_2$(TPM + 1) reads. Abbreviations: T = primary breast tumor, N = adjacent normal breast tissue, TPM = Transcripts Per Million reads.

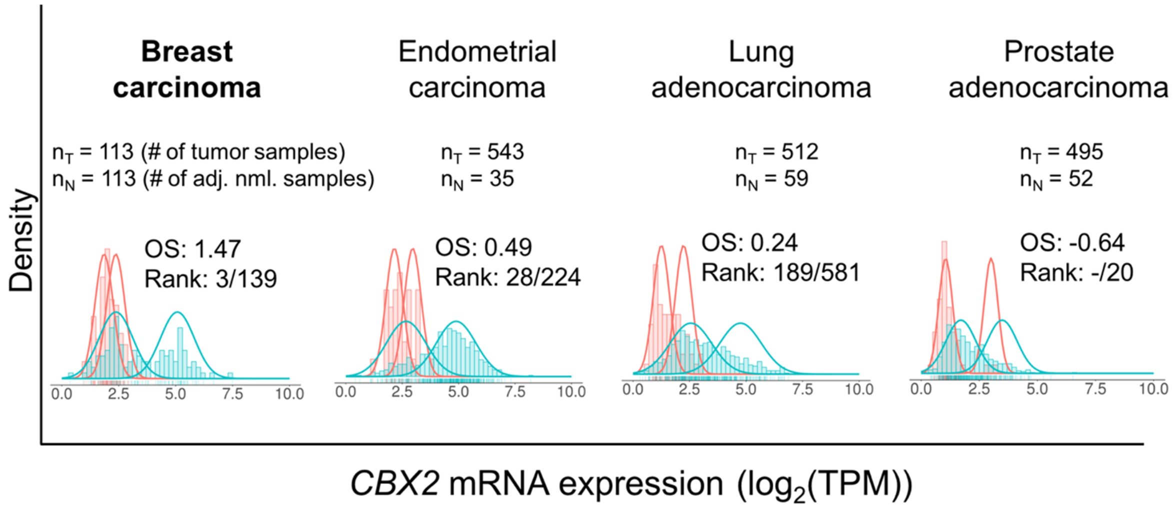

Supplementary Figure 13. Expression profiles of *CBX2* in four distinct tumor types and in adjacent normal tissue. The number of samples from each tumor type are shown. Rankings for each dataset are among the genes that passed filters applied to the original breast cancer dataset ($0.2 > \pi_T$ & $\pi_N > 0.8$, selectivity index > 0.99). OS = oncomix score.

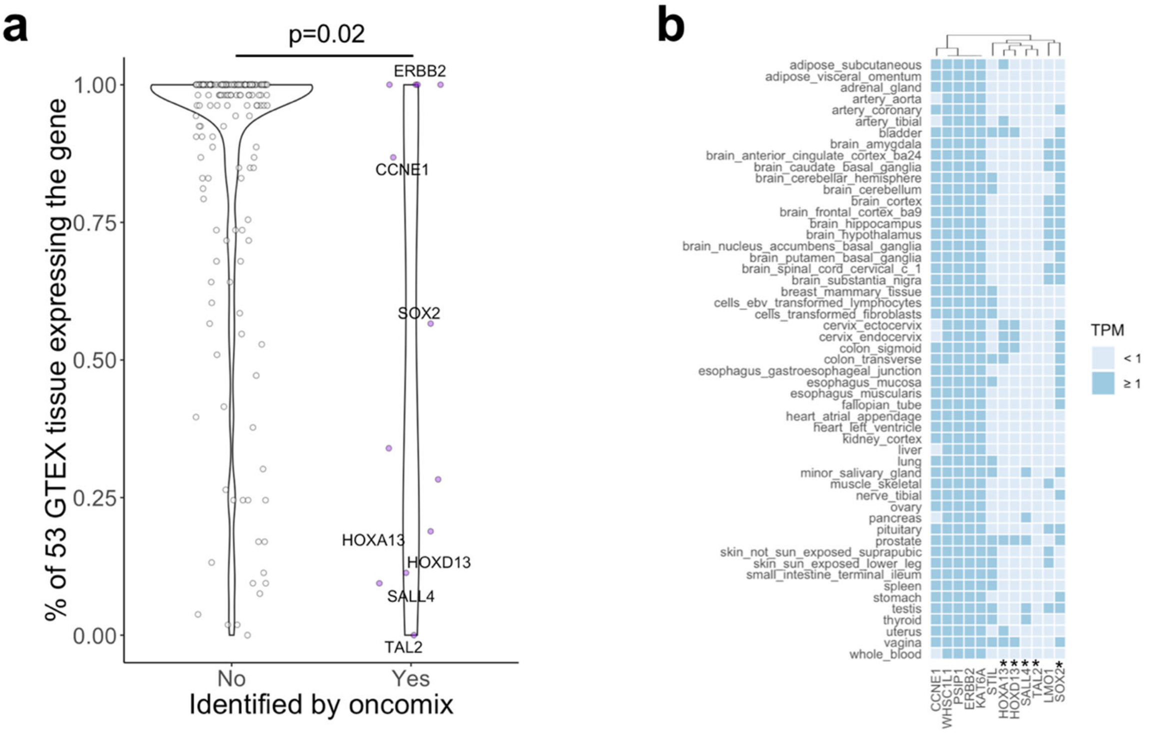

Supplementary Figure 14. Expression of oncogenes from the Cancer Gene Census within normal adult tissue. A) Each point in this violin plot represents a gene, and each gene was grouped on the x-axis according to whether it was identified by oncomix. The y-axis represents the percentage of tissues in GTEx that have a TPM > 1 for the gene. Prior studies have used a threshold of 1 TPM to classify a gene as expressed or not[15]. P-value was calculated using student's t-test (two-sided). B) Each column in the heatmap represents one of the 12 known oncogenes identified by oncomix, and each row represents a tissue collected in GTEx. Each cell in the heatmap represents a binary version of the median transcripts per million (TPM) value for a gene across all tissues obtained from the GTEx database. The asterisks indicate genes that are associated with mammalian embryogenesis in the literature as of March 2018 (see main text for references).

References

1. Moon TK. The expectation-maximization algorithm. *IEEE Signal Process. Mag.* **96**, 47–60 (1996).

2. Bamford S, Dawson E, Forbes S, Clements J, Pettett R, Dogan A, et al. The COSMIC (Catalogue of Somatic Mutations in Cancer) database and website. *Br. J. Cancer*. **2**, 355–358 (2004).

3. Futreal PA, Coin L, Marshall M, Down T, Hubbard T, Wooster R, et al. A census of human cancer genes. *Nat. Rev. Cancer*. **4**, 177–183 (2004).

4. Salsi V, Zappavigna V. Hoxd13 and Hoxa13 directly control the expression of the EphA7 ephrin tyrosine kinase receptor in developing limbs. *J. Biol. Chem.* **281**, 1992–1999 (2006).

5. Ellis P, Fagan BM, Magness ST, Hutton S, Taranova O, Hayashi S, et al. SOX2, a persistent marker for multipotential neural stem cells derived from embryonic stem cells, the embryo or the adult. *Dev. Neurosci.* **26**, 148–165 (2004).

6. Bucher K, Sofroniew M V., Pannell R, Impey H, Smith AJH, Torres EM, et al. The T cell oncogene Tal2 is necessary for normal development of the mouse brain. *Dev. Biol.* **227**, 533–544 (2000).

7. Zhang J, Tam W-L, Tong GQ, Wu Q, Chan H-Y, Soh B-S, et al. Sall4 modulates embryonic stem cell pluripotency and early embryonic development by the transcriptional regulation of Pou5f1. *Nat. Cell Biol.* **8**, 1114–1123 (2006).

8. Venables WN, Ripley BD. Modern Applied Statistics with S. 4th ed. 2002.

9. Triche T. FDb.InfiniumMethylation.hg19: Annotation package for llumina Infinium DNA methylation probes. **R package**, (2014).

10. Lawrence M, Gentleman R, Carey V. rtracklayer: An R package for interfacing with genome browsers. *Bioinformatics*. **25**, 1841–1842 (2009).

11. Bioconductor. TxDb.Hsapiens.UCSC.hg38.knownGene: Annotation package for TxDb. **R package**, (2016).

12. Paquet ER, Hallett MT. Absolute assignment of breast cancer intrinsic molecular subtype. *J. Natl. Cancer Inst.* **107**, 1–9 (2015).

13. Landt SG, Marinov GK, Kundaje A, Kheradpour P, Pauli F, Batzoglou S, et al. ChIP-seq guidelines and practices of the ENCODE and modENCODE consortia. *Genome Res.* **22**, 1813–1831 (2012).

14. The ENCODE Project Consortium. An integrated encyclopedia of DNA elements in the human genome. *Nature*. **489**, 57–74 (2012).

15. Liu P, Sanalkumar R, Bresnick EH, Keleş S, Dewey CN. Integrative analysis with ChIP-seq advances the limits of transcript quantification from RNA-seq. *Genome Res.* **26**, 1124–1133 (2016).
