# Supplementary material for: A novel approach to modelling transcriptional heterogeneity identifies the oncogene candidate CBX2 in invasive breast carcinoma
Source: Br J Cancer. 2019 Mar 1;120(7):746–53. doi: 10.1038/s41416-019-0387-8 (PMC6462018; doi:10.1038/s41416-019-0387-8)
Supplement: Supplementary file 2 — Code Associated with all Figures and Tables Generated in the Manuscript [file 41416_2019_387_MOESM2_ESM.html]

A novel approach to modeling transcriptional heterogeneity identifies the oncogene candidate CBX2 in invasive breast carcinoma


# A novel approach to modeling transcriptional heterogeneity identifies the oncogene candidate CBX2 in invasive breast carcinoma

#### *Daniel G. Pique, Cristina Montagna, John M. Greally, and Jessica C. Mar*

#### *2018-11-20*

1. Load the necessary libraries.

```
#statistical modeling
library(mclust)
library(oncomix)
library(glmnet)
library(limma)
library(survival)
library(AUC)

#bioinformatics packages
library(TCGAbiolinks)
library(AIMS)
library(GSA)
library(GenomicRanges)
library(TxDb.Hsapiens.UCSC.hg38.knownGene)
library(FDb.InfiniumMethylation.hg19)
library(rtracklayer)
library(GenomicDataCommons)
library(biomaRt)

#data wrangling, visualization and file organlization
library(here)
library(XML)
library(SummarizedExperiment)
library(ggrepel)
library(RColorBrewer)
library(ggdendro)
library(readxl)
library(tidyverse)
library(janitor)

#set seed for analysis
set.seed(100)
```

## Figure 1

1. Download the TCGA mRNA expression data using `GDCtools` from the TCGAbiolinks package.

```
if(file.exists(here("processed_data", "tcga-exp-brca.rds"))){
  e <- readRDS(file = here("processed_data", "tcga-exp-brca.rds"))
} else {
  eq <- GDCquery(project = "TCGA-BRCA",
              data.category = c("Transcriptome Profiling"),
              data.type = c("Gene Expression Quantification"),
              workflow.type = "HTSeq - FPKM")
  GDCdownload(eq, directory = here("raw_data"))
  e <- GDCprepare(eq,directory = here("raw_data"), save=F)
  #saveRDS(object = e, file = here("processed_data", "tcga-exp-brca.rds"))
}
```

2. Clean the dataset. Remove FFPE tissues, transform using the mRNA expression data using log2(TPM), and select only breast tumor samples with adjacent normal tissue.

```
#remove FFPE samples
dim(e)
```

```
## [1] 56716  1222
```

```
e <- e[,!e$is_ffpe]
dim(e)
```

```
## [1] 56716  1206
```

```
#add technical variables
colData(e)$hosp <- substr(rownames(colData(e)), 6,7)
colData(e)$seqPlate <- substr(rownames(colData(e)), 22,25)

###remove all metaData columns that are NA
colData(e) <- colData(e)[,colSums(is.na(colData(e)))<nrow(colData(e))]

##TPM normalization, then log2(tpm+1) transform
eu <- assay(e)
eu <- scale(eu, center=FALSE, scale=colSums(eu)) * 1000000
eu <- log(eu+1, 2)
assay(e) <- eu
rm(eu)

#only keep the matched normals (11's)...
en <- e[,substr(colnames(e),14,15) == "11"]

#...and their associated cancer samples (01's).
et <- e[,substr(colnames(e),14,15) == "01"]
et <- et[,!duplicated(substr(colnames(et),1,12))] #remove duplicates

sharedId <- intersect(substr(colnames(en), 1,12), substr(colnames(et), 1,12))

en <- en[,en$patient %in% sharedId]
et <- et[,et$patient %in% sharedId]

print(dim(et)); print(dim(en))
```

```
## [1] 56716   111
```

```
## [1] 56716   111
```

```
#remove the male patient
et <- et[,colData(et)$gender == "female"]
en <- en[,colData(en)$gender == "female"]

print(dim(et)); print(dim(en))
```

```
## [1] 56716   110
```

```
## [1] 56716   110
```

```
#order the et and en matrix columns so that they are the same
et <-  et[,order(et$patient)]
en <-  en[,order(en$patient)]
```

3. Map ENSG to HGNC symbols. Retain only protein coding genes, and merge duplicated gene symbols. Filter out genes where <20% of patients have a non zero expression value, and get the isoforms in common between the two.

```
#select only protein-coding genes
ensg_to_prot <- read.table(file=here("raw_data","ensembl", 
                                     "ensg_protein_class3.txt"), 
                           sep= "\t", header=TRUE, stringsAsFactors = FALSE)
ensg_to_prot <- ensg_to_prot[ensg_to_prot$Gene.type == "protein_coding",]
#must have an hgnc_symbol
ensg_to_prot <- ensg_to_prot[ensg_to_prot$NCBI.gene.ID != "",]

#drop transcripts that don't map to protein-coding ensembl gene Ids
et <- et[rownames(et) %in% ensg_to_prot$Gene.stable.ID ,]
en <- en[rownames(en) %in% ensg_to_prot$Gene.stable.ID ,]
print(dim(et)); print(dim(en))
```

```
## [1] 19090   110
```

```
## [1] 19090   110
```

```
#Filter out genes where <20% of patients have a non zero expression value
et <- et[rowSums(assay(et)==0)<=ncol(et)*.20,] 
en <- en[rowSums(assay(en)==0)<=ncol(en)*.20,] 
print(dim(et)); print(dim(en))
```

```
## [1] 16296   110
```

```
## [1] 16599   110
```

```
#get the isoforms in common between the two
genes_in_common <- intersect(rownames(et), rownames(en))
et <- et[genes_in_common,]
en <- en[genes_in_common,]
print(dim(et)); print(dim(en))
```

```
## [1] 16156   110
```

```
## [1] 16156   110
```

4. Check for confounding variables (namely, sequencing plate and tissue source site (aka hospital of procurement)). Use PCA to look for batch effect, similar to supplemental figures S10.2-S10.3 in the flagship endometrial TCGA cancer paper.

```
pca <- prcomp(x = t(cbind(assay(et), assay(en))), scale. = T, center = T)

#proportion of variance explained by first 2 pc
propVarExpl <- pca$sdev^2 / sum(pca$sdev^2)
barplot(propVarExpl[1:20], names.arg=1:20, xlab="Principal Comp",
        ylab="Prop. Variance Explained")
```

```
## Color by sequencing plate. Expect to see a rainbow 
## (no clustering by sequencing plate) if there is no major batch effect
plot(pca$x[,1:2], col = as.factor(c(colData(et)$seqPlate, colData(en)$seqPlate)))
```

```
#color by hospital of procurement
plot(pca$x[,1:2], col = as.factor(c(colData(et)$hosp, colData(en)$hosp)))
```

```
## color by whether the sample is tumor or normal. 
## This is where most of the variation is.
plot(pca$x[,1:2], col = as.factor(c(rep("tum", ncol(et)), 
                                    rep("nml", ncol(en)))))
```

Note that the main separation is between tumor and adjacent normal samples, mainly along PC1. We have no visually apparent clusters of samples by tissue source site or sequencing plate. This is a good thing!

## Figure 2

5. Next, we run oncomix on this gene expression dataset. The gene expression data are mapped to ensembl gene symbols.

```
if(file.exists(here("processed_data", "mmp.rds"))){
    mmp <- readRDS(file = here("processed_data", "mmp.rds"))
} else {
    mmp <- mixModelParams(exprNml = assay(en), exprTum = assay(et))
    saveRDS(mmp, file = here("processed_data", "mmp.rds"))
}

#select genes that have at least 20% of patients in each group and with an SI> 0.99
gene_filt_pi <- rownames(mmp)[mmp$tPi1 < 0.8 & mmp$tPi1 > 0.2 & 
                              mmp$nPi1 < 0.8 & mmp$nPi1 > 0.2]
gene_filt_pi_tum <- rownames(mmp)[mmp$tPi1 < 0.8 & mmp$tPi1 > 0.2]
gene_filt_pi_nml <- rownames(mmp)[mmp$nPi1 < 0.8 & mmp$nPi1 > 0.2]
length(gene_filt_pi_tum); length(gene_filt_pi_nml)
```

```
## [1] 8754
```

```
## [1] 7011
```

```
gene_filt_pi_si <- rownames(mmp)[mmp$tPi1 < 0.8 & mmp$tPi1 > 0.2 & 
                   mmp$nPi1 < 0.8 & mmp$nPi1 > 0.2 & mmp$SI > 0.99]
```

6. Next, we plot all the genes

```
source(here("scripts", "2017_12_suppl_scripts.R"))

top5genes <- head(gene_filt_pi_si, 5)
mmp2 <- mmp[gene_filt_pi,]
idx2replace <- sapply(top5genes, grep, rownames(mmp2))
geneIdsTop5 <- sapply(top5genes, function(x) ensg_to_prot$HGNC.symbol[grep(x, ensg_to_prot$Gene.stable.ID)])
rownames(mmp2)[idx2replace] <- geneIdsTop5

#show dimensions of the filtered dataset
dim(mmp2)
```

```
## [1] 3823   12
```

```
dim(mmp2 %>% filter(SI>0.99))
```

```
## Warning: package 'bindrcpp' was built under R version 3.4.4
```

```
## [1] 154  12
```

```
dim(mmp2 %>% filter(SI<0.99))
```

```
## [1] 3669   12
```

```
#show the statistics for the top5 genes
mmp2$geneNames <- rownames(mmp2)
mmp2 %>% dplyr::filter(geneNames %in% geneIdsTop5)
```

```
##         nMu1      nMu2       nVar      nPi1      tMu1     tMu2      tVar
## 1 0.16984400 0.2011481 0.07545295 0.5457794 1.3920623 4.433789 1.0926988
## 2 2.01932909 2.4169221 0.45999871 0.6140423 2.1950228 6.348791 1.6388138
## 3 1.88282562 2.3464588 0.16567905 0.6763193 2.3873075 5.054060 0.5561141
## 4 0.09438852 0.1249559 0.02950956 0.5685474 0.8106417 2.589987 0.3146127
## 5 1.52736530 1.8303487 0.25922094 0.6286604 1.6849682 4.181181 0.6391453
##        tPi1 deltaMu2  deltaMu1        SI    score geneNames
## 1 0.6467758 4.232641 1.2222183 1.0000000 1.842271      EPYC
## 2 0.6352695 3.931869 0.1756937 0.9909091 1.642296     NELL2
## 3 0.5785395 2.707601 0.5044819 1.0000000 1.481326      CBX2
## 4 0.4966407 2.465031 0.7162532 1.0000000 1.404656   SLC24A2
## 5 0.7757700 2.350832 0.1576029 0.9909091 1.283091      LAG3
```

```
#plot a histogram of the known scores

reds = brewer.pal(n = 9, name = "Reds")
alph = 0.8
#show the distribution of selectivity indices across genes
ggplot(mmp2, aes(x=SI)) +
  geom_histogram(binwidth = 0.02, color="grey", alpha=alph, aes(fill=SI>0.99)) + 
  theme_classic() + scale_fill_manual(values=reds[c(3,7)]) + scale_y_continuous(expand = c(0, 0))  + xlab("Selectivity Index (SI)") + ylab("Frequency") + theme(text=element_text(size=20, color="black"), legend.position="none")
```

```
#show the distribution of oncomix scores across genes
ggplot(mmp2, aes(x=score, y=..density.., fill=SI > 0.99)) +
  geom_histogram(data=subset(mmp2, SI<0.99), 
                 aes(x=score, y=..density.., fill=SI > 0.99), 
                 binwidth = 0.1, color="grey", alpha=alph) + 
  geom_histogram(data=subset(mmp2, SI>0.99), 
                 aes(x=score, y=..density.., fill=SI > 0.99), 
                 binwidth = 0.1, color="grey", alpha=alph) +
  theme_classic() + 
  scale_fill_manual(values=reds[c(3,7)]) + 
  scale_y_continuous(expand = c(0, 0)) + 
  theme(text=element_text(size=20, color="black"), legend.position="none") + 
  ylab("Density") + xlab("Oncomix Score")
```

7. Next, we’re going to plot the distributions of the top 5 selected isoforms with an SI > `selIdx_thresh`. We can also show the genes both before and after the selectivity index (ie low SI vs high SI).

```
gglayers = list(
    theme(axis.text.x = element_text(size=30),
          plot.title=element_text(size=30)),
  xlim(-0.2,10), xlab(""), ylab("")
)


for(i in 1:length(top5genes)){
  hgnc_gene_symb <- ensg_to_prot %>% 
      dplyr::filter(Gene.stable.ID == top5genes[i]) %>% 
      dplyr::select(HGNC.symbol) %>% 
      as.character()
  gglayers[["title"]] <- ggtitle(hgnc_gene_symb)
  plotGeneHistUD(mmp, assay(en), assay(et), top5genes[i], linesz=1.5, gglayers)
  ggsave(filename = here("output", paste0(Sys.Date(),"_",top5genes[i], "_histog.png")),
         width = 7, height=5, units = "in")
}
```

8. Add in the AIMS molecular subtypes. Also, show the clinical variables. Note that AIMS requires entrez Ids (aka NCBI gene IDs), and that there is a many-to-many mapping between ENSG ids and entrez ids.

```
ensg_to_prot_filt <- ensg_to_prot[ensg_to_prot$Gene.stable.ID %in% rowData(et)$ensembl_gene_id,] 

while(length(ensg_to_prot_filt$Gene.stable.ID) != length(unique(ensg_to_prot_filt$Gene.stable.ID))){
  ensg_to_prot_filt <-  ensg_to_prot_filt[!duplicated(ensg_to_prot_filt$Gene.stable.ID),]
}

rowData(et) = dplyr::left_join(data.frame(rowData(et)), ensg_to_prot_filt, by = c("ensembl_gene_id" = "Gene.stable.ID"))
aims_subtypes <- applyAIMS(assay(et),rowData(et)$NCBI.gene.ID)
```

```
## Current k = 20
```

```
colData(et)$aims_subtypes <- aims_subtypes$cl[,1]

table(colData(et)$aims_subtypes)
```

```
## 
##  Basal   Her2   LumA   LumB Normal 
##     17     25     36     25      7
```

```
table(colData(et)$tumor_stage)
```

```
## 
## not reported      stage i     stage ia    stage iia    stage iib 
##            1           16            4           34           29 
##   stage iiia   stage iiib   stage iiic     stage iv 
##           16            4            4            2
```

```
table(colData(et)$vital_status)
```

```
## 
## alive  dead 
##    66    44
```

```
table(colData(et)$race)
```

```
## 
##                     asian black or african american 
##                         1                         6 
##              not reported                     white 
##                         1                       102
```

```
summary(colData(et)$days_to_birth/-365.25)
```

```
##    Min. 1st Qu.  Median    Mean 3rd Qu.    Max.    NA's 
##   30.67   45.77   56.93   57.80   66.43   90.00       1
```

```
sd(colData(et)$days_to_birth/-365.25, na.rm = TRUE)
```

```
## [1] 14.53671
```

9. Next, we will conduct a differential expression analysis between tumor subgroups using limma, which performs a regularized 2 sample t-test. Groups will be defined for each oncogene candidate by differences in gene expression (either baseline or overexpressed) in the tumor samples. We begin by classifying tumors based on level of expression of these top 5 genes.

```
colData(et) <- cbind(colData(et), as.data.frame(classifyTumors(mmp, top5genes, et)))

hallmarkKeggReactome <- readHmKgRctmepathways(gsea_path = here("raw_data", "genesets"))

B <- nrow(et) - 1
qval_diffexp <- 1e-04
log2FC_diffexp <- 1
OR_pathway <- 20  #this is the Odds Ratio for pathway analysis
qval_pathway <- 1e-20
# save output of differentially expressed genes
allDeGenes.l <- vector("list", length(top5genes))
upregDeGenes.l <- vector("list", length(top5genes))
downregDeGenes.l <- vector("list", length(top5genes))
upregDePaths.l <- vector("list", length(top5genes))
downregDePaths.l <- vector("list", length(top5genes))
for (i in 1:length(top5genes)) {
    classif <- paste0("cl_", top5genes[i])
    vars <- c(classif, "seqPlate", "hosp")
    colData_e_stdze <- colData(et)[, vars]
    dsgn <- model.matrix(~colData(et)[, classif] + colData(et)[, "seqPlate"] + colData(et)[, 
        "hosp"])
    allDeGenes <- returnDeGenesLimma(assay(et)[rownames(assay(et)) != top5genes[i], 
        ], dsgn, colOfInt = 2, retAll = T)
    allDeGenes$ensgId <- rownames(allDeGenes)
    allDeGenes.l[[i]] <- allDeGenes
    
    upregDeGenes <- allDeGenes[allDeGenes$logFC > log2FC_diffexp & allDeGenes$adj.P.Val < 
        qval_diffexp, ]  #473 downregulated
    upregDeGenes.l[[i]] <- upregDeGenes
    downregDeGenes <- allDeGenes[allDeGenes$logFC < -log2FC_diffexp & allDeGenes$adj.P.Val < 
        qval_diffexp, ]  #118 upregulated
    downregDeGenes.l[[i]] <- downregDeGenes
    # overrepresentation analysis
    if (nrow(upregDeGenes) > 1) {
        upregDeGenesEntrez <- unique(rowData(et)[rowData(et)$ensembl_gene_id %in% 
            rownames(upregDeGenes), "NCBI.gene.ID"])
        upregDeGenesEntrez_gse <- gseWrapper(B, upregDeGenesEntrez, hallmarkKeggReactome$genesets, 
            hallmarkKeggReactome$geneset.names, qval = qval_pathway, OR = OR_pathway)
        upregDePaths.l[[i]] <- upregDeGenesEntrez_gse
        createForestDF(upregDeGenesEntrez_gse, paste0("Overrepresentation Analysis for Upregulated genes in \nTriple Positive tumors (OR > ", 
            OR_pathway, " and q < ", qval_pathway, ")\n", top5genes[i]))
        ggsave(filename = here("output", paste0(top5genes[i], "_up.png")), width = 12, 
            height = 7, units = "in")
    }
    if (nrow(downregDeGenes) > 1) {
        downregDeGenesEntrez <- unique(rowData(et)[rowData(et)$ensembl_gene_id %in% 
            rownames(downregDeGenes), "NCBI.gene.ID"])
        downregDeGenesEntrez_gse <- gseWrapper(B, downregDeGenesEntrez, hallmarkKeggReactome$genesets, 
            hallmarkKeggReactome$geneset.names, qval = qval_pathway, OR = OR_pathway)
        downregDePaths.l[[i]] <- downregDeGenesEntrez_gse
        createForestDF(downregDeGenesEntrez_gse, paste0("Overrepresentation Analysis for Downregulated genes in \nTriple Positive tumors (OR > ", 
            OR_pathway, " and q < ", qval_pathway, ")\n", top5genes[i]))
        ggsave(filename = here("output", paste0(top5genes[i], "_down.png")), width = 12, 
            height = 7, units = "in")
    }
}
```

```
## Coefficients not estimable: colData(et)[, "hosp"]GI
```

```
## Coefficients not estimable: colData(et)[, "hosp"]GI 
## Coefficients not estimable: colData(et)[, "hosp"]GI
```

```
## Coefficients not estimable: colData(et)[, "hosp"]GI
```

```
## Coefficients not estimable: colData(et)[, "hosp"]GI
```

```
# check to see which genes had differentialy expressed pathways
names(allDeGenes.l) <- top5genes
names(upregDePaths.l) <- top5genes
names(downregDePaths.l) <- top5genes

dim_upreg <- lapply(upregDePaths.l, nrow)
dim_downreg <- lapply(downregDePaths.l, nrow)
dim_upreg <- unlist(lapply(dim_upreg, function(x) ifelse(is.null(x), 0, x)))
dim_downreg <- unlist(lapply(dim_downreg, function(x) ifelse(is.null(x), 0, x)))

# which are non-zero?
genesWithDePathsUpreg <- names(dim_upreg)[dim_upreg != 0]
genesWithDePathsDownreg <- names(dim_downreg)[dim_downreg != 0]
genesWithDePathsUpreg
```

```
## [1] "ENSG00000173894" "ENSG00000155886"
```

```
genesWithDePathsDownreg
```

```
## character(0)
```

## Supplementary Table 2

9b. Make a summary table of the pathway analysis from the 5 genes.

```
numUpGenes <- unlist(lapply(upregDeGenes.l, nrow))
numDownGenes <- unlist(lapply(downregDeGenes.l, nrow))
de_pathway_summ <- cbind(upregulatedGenes = numUpGenes, downregulatedGenes = numDownGenes, 
    numbPathwaysUp = dim_upreg, numbPathwaysDown = dim_downreg, pathwaySummary = c("-", 
        "-", "cell cycle", "matrix", "immune")) %>% 
  as.data.frame() %>% 
  rownames_to_column("ensg_id") %>% 
  left_join(ensg_to_prot, by = c(ensg_id = "Gene.stable.ID")) %>% dplyr::select(HGNC.symbol, 
  upregulatedGenes, downregulatedGenes)

knitr::kable(de_pathway_summ)
```

| HGNC.symbol | upregulatedGenes | downregulatedGenes |
| --- | --- | --- |
| EPYC | 4 | 0 |
| NELL2 | 0 | 0 |
| CBX2 | 73 | 17 |
| SLC24A2 | 241 | 1 |
| LAG3 | 105 | 2 |

## Supplementary Table 3

Print the pathways that are enriched

```
orVars = c("estimate.odds ratio", "conf.int1", "conf.int2")
upregDePaths.l.df <- do.call(rbind, upregDePaths.l) %>%
  rownames_to_column("ensg") %>% 
  separate(ensg, into = c("gene", "toRm"),fill="right") %>% 
  left_join(ensg_to_prot_filt, by = c("gene" = "Gene.stable.ID")) %>%
  dplyr::select(HGNC.symbol, geneset.names, bhPval, `estimate.odds ratio`, conf.int1, conf.int2) %>% 
  as_tibble() %>%
  mutate_at(vars(orVars), as.numeric) %>%
  mutate_at(vars(orVars), round) %>%
  mutate(bhPval = sprintf("%3.1e", bhPval)) %>%
  mutate("95% CI" =  paste0(conf.int1, "-", conf.int2)) %>%
  mutate(geneset.names =gsub("_"," ",str_to_lower(geneset.names))) %>%
  dplyr::select(-conf.int1, -conf.int2) %>%
  dplyr::rename("Oncogene Candidate"= HGNC.symbol, "Geneset"=geneset.names,"q value" = bhPval, "Odds Ratio" = `estimate.odds ratio`)

knitr::kable(upregDePaths.l.df)
```

| Oncogene Candidate | Geneset | q value | Odds Ratio | 95% CI |
| --- | --- | --- | --- | --- |
| CBX2 | hallmark g2m checkpoint | 2.2e-30 | 54 | 31-91 |
| CBX2 | hallmark e2f targets | 1.3e-25 | 44 | 25-75 |
| SLC24A2 | hallmark epithelial mesenchymal transition | 1.3e-59 | 37 | 26-53 |

10. Volcano plot for CBX2 – had many de genes involved in cell cycle. Show genes involved in top ranked pathway (cell cycle)

```
#display the genes from the top pathway
#ENSG00000173894 = CBX2
topGeneSet<- upregDePaths.l[["ENSG00000173894"]][1,"geneset.names"]

diffExprDf = allDeGenes.l[["ENSG00000173894"]]

rownames(ensg_to_prot_filt) <- ensg_to_prot_filt$Gene.stable.ID
entrezIdsGeneSet = hallmarkKeggReactome[[1]][[topGeneSet]]

plot.volcano.gsea(hallmarkKeggReactome, diffExprDf, topGeneSet, log2FC_diffexp = log2FC_diffexp, qval_diffexp = qval_diffexp, ensg_map = ensg_to_prot_filt, brew.color.set1 = 3, propSubset = 0.4)
```

```
ggsave(filename = here("output", paste0(topGeneSet, "_volcano.png")), width = 10, height=7, units = "in")
```

## Supplementary Table 4

Map ensg to chromosome, gene symbol, description using biomart. Generate a table of the differentially expressed genes

```
ensg_to_prot_filt2 <- ensg_to_prot_filt %>% rename(ensgId = "Gene.stable.ID")

diffExprDf2 <- diffExprDf %>% 
  filter(logFC > log2FC_diffexp, adj.P.Val < qval_diffexp) %>%
  left_join(ensg_to_prot_filt2, by="ensgId") %>% 
  mutate(inTopGeneSet = NCBI.gene.ID %in%
           hallmarkKeggReactome[[1]][[topGeneSet]]) %>% 
  filter(inTopGeneSet)

#if(file.exists(here("processed_data", "ensg_de_tbl.rds"))){
#    ensg_de_tbl <- readRDS(here("processed_data", "ensg_de_tbl.rds"))
#} else {
    ensembl <- useMart("ensembl",dataset="hsapiens_gene_ensembl")
    ensg_de_tbl <- getBM(mart=ensembl, 
                         filters=c("ensembl_gene_id"),
                         attributes=c("ensembl_gene_id", "entrezgene", 
                                      "description", "chromosome_name","band"),
                         values = diffExprDf2$ensgId)
    ensg_de_tbl <- rename(ensg_de_tbl, ensgId = "ensembl_gene_id")
    saveRDS(ensg_de_tbl, here("processed_data", "ensg_de_tbl.rds"))
#}

diffExprDf3 <- left_join(diffExprDf2, ensg_de_tbl, by="ensgId") %>%
  select(HGNC.symbol, description, chromosome_name, band, logFC, adj.P.Val) %>%
  mutate(Chromosome=paste0(chromosome_name,band)) %>%
  arrange(chromosome_name, band) %>%
  select(-chromosome_name, -band) %>% 
  mutate(logFC = round(logFC, 2)) %>% 
  mutate(description = gsub("\\[[^\\]]*\\]", "", description, perl=TRUE)) %>% 
  rename("log2(Fold Change)" = "logFC", "q value" = "adj.P.Val", "HGNC symbol" = "HGNC.symbol",  "Description" = "description")

knitr::kable(diffExprDf3)
```

| HGNC symbol | Description | log2(Fold Change) | q value | Chromosome |
| --- | --- | --- | --- | --- |
| KIF2C | kinesin family member 2C | 1.55 | 1.30e-06 | 1p34.1 |
| RAD54L | RAD54 like | 1.26 | 5.80e-06 | 1p34.1 |
| CDC20 | cell division cycle 20 | 1.63 | 9.30e-06 | 1p34.2 |
| E2F2 | E2F transcription factor 2 | 1.14 | 9.14e-05 | 1p36.12 |
| EXO1 | exonuclease 1 | 1.30 | 6.97e-05 | 1q43 |
| CENPA | centromere protein A | 1.59 | 7.00e-07 | 2p23.3 |
| BUB1 | BUB1 mitotic checkpoint serine/threonine kinase | 1.35 | 6.30e-06 | 2q13 |
| CENPE | centromere protein E | 1.09 | 6.48e-05 | 4q24 |
| CCNA2 | cyclin A2 | 1.29 | 5.55e-05 | 4q27 |
| MAD2L1 | mitotic arrest deficient 2 like 1 | 1.00 | 8.91e-05 | 4q27 |
| TTK | TTK protein kinase | 1.29 | 1.06e-05 | 6q14.1 |
| EZH2 | enhancer of zeste 2 polycomb repressive complex 2 subunit | 1.01 | 1.26e-05 | 7q36.1 |
| CDK1 | cyclin dependent kinase 1 | 1.17 | 7.80e-05 | 10q21.2 |
| TROAP | trophinin associated protein | 1.35 | 1.07e-05 | 12q13.12 |
| ESPL1 | extra spindle pole bodies like 1, separase | 1.17 | 3.66e-05 | 12q13.13 |
| PLK1 | polo like kinase 1 | 1.42 | 1.37e-05 | 16p12.2 |
| ORC6 | origin recognition complex subunit 6 | 1.08 | 2.86e-05 | 16q11.2 |
| SLC7A5 | solute carrier family 7 member 5 | 1.63 | 6.07e-05 | 16q24.2 |
| BIRC5 | baculoviral IAP repeat containing 5 | 1.65 | 1.30e-06 | 17q25.3 |
| NDC80 | NDC80, kinetochore complex component | 1.18 | 5.77e-05 | 18p11.32 |
| CDC25B | cell division cycle 25B | 1.14 | 2.86e-05 | 20p13 |
| TPX2 | TPX2, microtubule nucleation factor | 1.44 | 1.14e-05 | 20q11.21 |
| E2F1 | E2F transcription factor 1 | 1.27 | 3.27e-05 | 20q11.22 |
| MYBL2 | MYB proto-oncogene like 2 | 2.06 | 1.30e-06 | 20q13.12 |
| UBE2C | ubiquitin conjugating enzyme E2 C | 1.64 | 1.58e-05 | 20q13.12 |
| AURKA | aurora kinase A | 1.42 | 3.10e-06 | 20q13.2 |
| CDC45 | cell division cycle 45 | 1.25 | 4.00e-05 | 22q11.21 |

11. Multi-omic prediction of oncogene candidate overexpression using logistic regression. First, we need to load in the clinical, CNV, and methylation data from TCGA.

```
## identify all available methylation data for tumors with matched normal. 
## do this with the GenomicDataCommons package (create a manifest), then
## use TCGAbiolinks to download and read in the data

fieldsToReturn <- c("file_id", "file_name", "associated_entities.entity_submitter_id", "analysis.workflow_link", "analysis.workflow_type", "analysis.workflow_version")

if(!file.exists(here("processed_data", "tcga-methyl-brca.rds"))){

  ge_manifest = files() %>% 
      GenomicDataCommons::select(fieldsToReturn) %>%  
      GenomicDataCommons::filter( ~ cases.project.project_id == 'TCGA-BRCA' &
                type == 'methylation_beta_value' &
                platform ==  "Illumina Human Methylation 450" & 
                cases.samples.sample_type == "Primary Tumor" &
                cases.submitter_id == substr(colnames(et), 1,12)) %>%
      results_all() %>% data.frame

  #download the files
  gdcClientPath <- "C:/Users/Daniel/software/gdc-client_v1.2.0_Windows_x64/gdc-client.exe"
  system(paste0(gdcClientPath, " download ", paste(ge_manifest$file_id,collapse = " ")))

  ## tumors with adjacent normal tissue
  mq <- GDCquery(project = "TCGA-BRCA",
                data.category = "DNA Methylation",
                platform="Illumina Human Methylation 450", #,
                barcode = ge_manifest$associated_entities.entity_submitter_id)
  GDCdownload(mq, directory = here("raw_data"), files.per.chunk = 3)

  m <- GDCprepare(mq,directory = here("raw_data"), save=F)
  #saveRDS(object = m, file = here("processed_data", "tcga-methyl-brca.rds"))

} else {
  m <- readRDS(file = here("processed_data", "tcga-methyl-brca.rds"))
}

m <- m[substr(rownames(assay(m)),1,2) == "cg",]


if(file.exists(here("processed_data", "cnv_cbx2.rds"))){
  cnv2 <- readRDS(here("processed_data", "cnv_cbx2.rds"))
} else {
  cnv_manifest_map = files() %>% 
      GenomicDataCommons::select(fieldsToReturn) %>%  
      GenomicDataCommons::filter(~data_type == 'Copy Number Segment' & 
               cases.project.project_id == 'TCGA-BRCA' & 
               cases.submitter_id == substr(colnames(et), 1,12) & 
               type == 'copy_number_segment' & 
               cases.samples.sample_type == "Primary Tumor") %>% 
      results_all() #108 samples avail
  
  cnv_manifest_map.df <- data.frame(cnv_manifest_map)
  #download the cnv data
  fnamescnv = lapply(cnv_manifest_map$id,gdcdata,
                  destination_dir=
                    here("raw_data", "TCGA-BRCA", "harmonized", "cnv"), 
                  overwrite=TRUE,
                  progress=FALSE)
  
  cnvList = lapply(fnamescnv, read.table, header = TRUE, sep = "\t",
                stringsAsFactors=F)
  cnv <- do.call(rbind, cnvList)
  
  cnv2 <- cnv_manifest_map.df %>% 
      tbl_df() %>% 
      separate(col = file_name,into = c("Sample", "toRm"), 
               sep = ".grch38.seg.txt") %>%
      dplyr::select(-toRm) %>%
      right_join(cnv, by="Sample")
  
  cnv2 <- GRanges(seqnames=paste0("chr",cnv2$Chromosome), 
                  ranges = IRanges(start = cnv2$Start, end=cnv2$End),
                  tcga_id=cnv2$associated_entities.entity_submitter_id,
                  seg_mean=cnv2$Segment_Mean,
                  num_probes=cnv2$Num_Probes)
  #saveRDS(cnv2, file= here("processed_data", "cnv_cbx2.rds"))
}        


#cnv2 has the tcga ids and the mean segment copy number all in one
```

Now we have our CNV and methylation data. Now, we need the tumor purity data (from the biospecimen supplement) and retrospective collection data (from the clinical data).

```
#downloaded the biospecimen_supplement - contains the % tumor cells
if(file.exists(here("processed_data", "tum_cells_df.rds"))){
  tum_cells.df <- readRDS(here("processed_data", "tum_cells_df.rds"))
} else {
  biosp_manifest_map = files() %>% 
    GenomicDataCommons::filter(~ cases.project.project_id == 'TCGA-BRCA' & 
             type == 'biospecimen_supplement' &
             cases.submitter_id == substr(colnames(et), 1, 12)) %>% 
    results_all()
  
  fnamesbiosp = lapply(biosp_manifest_map$id,gdcdata,
                  destination_dir=
                    here("raw_data", "TCGA-BRCA", "harmonized", "biosp"), 
                  overwrite=TRUE,
                  progress=FALSE)
  
  biospFiles <- list.files(here("raw_data", "TCGA-BRCA", "harmonized", "biosp"))

  tum_cells <- c()
  for(i in 1:length(biospFiles)){
    xmlp = xmlParse(here("raw_data", "TCGA-BRCA", "harmonized", "biosp", biospFiles[i]))
    xmlr = xmlRoot(xmlp)
    tum_c <- xmlr[["patient"]][["samples"]][["sample"]][["portions"]][["portion"]][["slides"]][["slide"]][["percent_tumor_cells"]][["text"]]
    tum_cells <- c(tum_cells, xmlValue(tum_c))
  }
  names(tum_cells) <- substr(biospFiles, 37, 48)
  tum_cells.df <- data.frame(tum_cells, stringsAsFactors = F)
  rownames(tum_cells.df) <- names(tum_cells)
  #saveRDS(tum_cells.df, file=here("processed_data", "tum_cells_df.rds"))
}

colData(et)$percent_tumor_cells <- as.numeric(tum_cells.df[colData(et)$patient,])
summary(colData(et)$percent_tumor_cells)
```

```
##    Min. 1st Qu.  Median    Mean 3rd Qu.    Max. 
##   15.00   65.25   75.00   73.28   85.00  100.00
```

```
sd(colData(et)$percent_tumor_cells)
```

```
## [1] 18.96793
```

```
#download the clinical_supplement - contains retrospective collection information

if(all(file.exists(here("processed_data", "retro_coll_df.rds"), 
               here("processed_data", "histo_status_df.rds"),
               here("processed_data", "mets_df.rds")))){
  retro_coll.df <- readRDS(here("processed_data", "retro_coll_df.rds"))
  histo_status.df <- readRDS(here("processed_data", "histo_status_df.rds"))
  mets_df <- readRDS(here("processed_data", "mets_df.rds"))
} else {
  clin_manifest_map = files() %>% 
    GenomicDataCommons::filter(~ cases.project.project_id == 'TCGA-BRCA' & 
             type == 'clinical_supplement' &
             cases.submitter_id == substr(colnames(et), 1, 12)) %>% 
    results_all()
  
  fnamesclin = lapply(clin_manifest_map$id,gdcdata,
                  destination_dir=
                    here("raw_data", "TCGA-BRCA", "harmonized", "clin"), 
                  overwrite=TRUE,
                  progress=FALSE)
  
  clinFiles <- list.files(here("raw_data", "TCGA-BRCA", "harmonized", "clin"))
  retro_coll <- c()
  histo_status <- c()
  node_count <- c()
  mets_ihc_status <- c()
  mets_he_status <- c()
  for(i in 1:length(clinFiles)){
    xmlp = xmlParse(here("raw_data", "TCGA-BRCA", "harmonized", "clin", clinFiles[i]))
    xmlr = xmlRoot(xmlp)
    retro <- xmlr[["patient"]][["tissue_retrospective_collection_indicator"]][["text"]]
    retro_coll <- c(retro_coll, xmlValue(retro))
    histo <- xmlr[["patient"]][["histological_type"]][["text"]]
    histo_status <- c(histo_status, xmlValue(histo))
    node <- xmlr[["patient"]][["lymph_node_examined_count"]]
    node_count <- c(node_count, xmlValue(node))
    mets_ihc <- xmlr[["patient"]][["number_of_lymphnodes_positive_by_ihc"]]
    mets_ihc_status <- c(mets_ihc_status, xmlValue(mets_ihc))
    mets_he <- xmlr[["patient"]][["number_of_lymphnodes_positive_by_he"]]
    mets_he_status <- c(mets_he_status, xmlValue(mets_he))
  }

  mets_df <- tibble(node_count, mets_ihc_status, mets_he_status) %>% 
    mutate_all(as.numeric) %>% 
    mutate_at(vars(starts_with("mets_")), funs(ifelse(is.na(.),0,.))) %>%
    mutate(final = ifelse(is.na(node_count), NA, 0)) %>%
    mutate(final = ifelse(mets_ihc_status > 0 | mets_he_status > 0, 1, final)) %>%
    data.frame()
  rownames(mets_df) = substr(clinFiles, 34, 45)
  
  names(retro_coll) <- substr(clinFiles, 34, 45)
  names(histo_status) <- substr(clinFiles, 34, 45)
  retro_coll.df <- data.frame(retro_coll, stringsAsFactors = FALSE) 
  histo_status.df <- data.frame(histo_status, stringsAsFactors = FALSE)
  #saveRDS(retro_coll.df, here("processed_data", "retro_coll_df.rds"))
  #saveRDS(histo_status.df, here("processed_data", "histo_status_df.rds"))
  #saveRDS(mets_df, here("processed_data", "mets_df.rds"))
}

colData(et)$retro_coll_yes1 <- ifelse(retro_coll.df[colData(et)$patient,"retro_coll"] == 
                                        "YES", 1, 0)
table(colData(et)$retro_coll_yes1)
```

```
## 
##  0  1 
## 22 88
```

```
colData(et)$histology <- histo_status.df[colData(et)$patient,]
table(colData(et)$histology)
```

```
## 
##    Infiltrating Ductal Carcinoma   Infiltrating Lobular Carcinoma 
##                               89                                6 
##              Medullary Carcinoma Mixed Histology (please specify) 
##                                2                               10 
##               Mucinous Carcinoma                   Other, specify 
##                                1                                2
```

```
colData(et)$nodal_mets <- mets_df[colData(et)$patient,"final"]
table(colData(et)$nodal_mets)
```

```
## 
##  0  1 
## 45 59
```

Now we need to aggregate and normalize our Data for logistic regression (predict whether oncogene is overexpressed or not). All data will be fit to the interval [0-1], and all categorical variables will be represented as dummy variables. We have 3 classes of variables: DNA methylation (ie all CpGs at gene locus), CNV/AIMS/mRNA expr in normal tissue, Clinical (Age, % tumor cells, Metastasis, Stage, Race, 5 year survival), and Technical (Retrospective collection, Hosp of Procurement, Seq Plate).

```
#Munging 5-year survival data
days_last_fu <- ifelse(is.na(colData(et)$days_to_death), colData(et)$days_to_last_follow_up,colData(et)$days_to_death)
days_last_fu <- ifelse(days_last_fu > 365.25 * 5, 365.25 * 5, days_last_fu)

vital_stat <- ifelse(colData(et)$vital_status == "alive", 0, 1)
vital_stat <- ifelse(days_last_fu >= 365.25 * 5, 0, vital_stat)

colData(et)$days_last_fu_5yr <- days_last_fu
colData(et)$vital_stat_5yr <- vital_stat

clin_vars_cat <- c("aims_subtypes", "race", "tumor_stage", "vital_stat_5yr")
clin_vars_cont <- c("percent_tumor_cells", "age_at_diagnosis")
tech_vars_cat <- c("retro_coll_yes1", "seqPlate", "hosp")
expr_cnv_cat <- c("aims_subtypes") #cnv and mRNA in nml tissue - extract separately
```

Now we need the DNA methylation and CNV variables. First, we create GRanges objects for the DNA methylation and CNV matrices. Then, we identify the gene coordinates from our top5 genes. We can then intersect the top5 gene coordinates with the DNA methylation and CNV objects to identify the copy number and methylation status at these gene loci.

```
#liftover coordinates to hg38
chain <- import.chain(here("raw_data", "liftover", "hg19ToHg38.over.chain"))

hm450 <- get450k() %>% 
  liftOver(chain) %>%
  unlist() %>%
  subset(names %in% rownames(m)) %>%
  subset(!duplicated(names)) %>% #remove duplicates
  data.frame(., row.names=names(.)) %>% 
  rownames_to_column("cgid") %>% 
  left_join(data.frame(rowData(m)), ., 
            by = c("Composite.Element.REF" = "cgid"))
```

```
## Fetching coordinates for hg19...
```

```
goodLiftOvIdx = !apply(hm450, 1, anyNA)
hm450 <- hm450[goodLiftOvIdx,]
m <- m[goodLiftOvIdx,]
#lifted-over coordinates of 450k array  
hm450_gr = GRanges(seqnames = as.character(pull(hm450, seqnames)), 
               ranges = IRanges(pull(hm450, start), pull(hm450, end)), 
               cgid = pull(hm450,Composite.Element.REF))
  
all.equal(rowData(m)$Composite.Element.REF, hm450_gr$cgid)
```

```
## [1] TRUE
```

```
rowData(m) <- hm450_gr

#then, intersect with the coordinates for the 6 genes
allGeneRange<-genes(TxDb.Hsapiens.UCSC.hg38.knownGene)
#genes_gr <- allGeneRange[as.character(rowData(et[top5genes,])$NCBI.gene.ID),]
#genes_gr$gene_symb <- as.character(rowData(et[top5genes,])$HGNC.symbol) 
genes_gr <- allGeneRange[as.character(rowData(et[top5genes,])$NCBI.gene.ID),]
genes_gr$gene_symb <- as.character(rowData(et[top5genes,])$HGNC.symbol) 

genes_gr$ensg <- top5genes

#add 1500 bp to the 5' end of the transcript
genes_gr_prom <- promoters(genes_gr, upstream = 1500, downstream = 0)
genes_gr2 <- punion(genes_gr,genes_gr_prom)
genes_gr2@elementMetadata <- genes_gr@elementMetadata

allExons <- exonsBy(TxDb.Hsapiens.UCSC.hg38.knownGene, by="gene") #get the exons
methSE <- rowData(m)$X


#this is how we get the CNV Status for a gene
cnvStatusAtLoci <- function(geneGr, cnvGr){
  cnv_subst <- subsetByOverlaps(cnvGr, geneGr)
  return(cnv_subst)
}
```

- Now, we have the methylation data and CNV data all in place. Perform logistic regression.

```
expr_cnv_cat <- c("aims_subtypes") #cnv and mRNA in nml tissue - extract separately
clin_vars_cat <- c("race", "tumor_stage", "vital_stat_5yr")
tech_vars_cat <- c("retro_coll_yes1", "seqPlate", "hosp")

expr_cnv_cont <- c("expr_nml_txue") #cnv and mRNA in nml tissue 
clin_vars_cont <- c("percent_tumor_cells", "age_at_diagnosis")
cpg_vars_cont <- c("Promoter", "Exon_first","Exon_middle", "Exon_last", "Intron")
mmCatVar <- colData(et) %>% 
  model.matrix(~ aims_subtypes + race + tumor_stage + vital_stat_5yr +
                 retro_coll_yes1 + seqPlate + hosp, .) %>%
  data.frame() %>% 
  rownames_to_column("tcga_id")%>%
  as_tibble() %>%
  mutate(tcga_id = substr(tcga_id, 1, 12))

contVar <- colData(et) %>%
  data.frame() %>%
  as_tibble() %>%
  dplyr::select(percent_tumor_cells, age_at_diagnosis, patient) %>%
  mutate(percent_tumor_cells=scales::rescale(percent_tumor_cells,to=c(0, 1))) %>%
  mutate(age_at_diagnosis=scales::rescale(age_at_diagnosis,to=c(0, 1))) %>%
  rename(tcga_id = patient)
  
allVarClass <- c(cpg_vars_cont, expr_cnv_cat, expr_cnv_cont, "seg_mean", clin_vars_cat, clin_vars_cont, tech_vars_cat)

allVarLabel <-
  c(
  "TSS1500",
  "First Exon",
  "Middle Exon",
  "Last Exon",
  "Intron",
  "AIMS subtypes",
  "Expr. in Normal Tissue",
  "CNV",
  "Race",
  "Stage",
  "5-year survival",
  "% Tumor Cells",
  "Age",
  "Retrospective Collection",
  "Seq. plate",
  "Hospital"
  )

lr_res_maxBeta.l <- list()
aucResults <- c()
for(i in 1:length(genes_gr2)){
  cpg_gr <- findAndAnnotateCpGsInGene(genes_gr2[i], allExons, methSE, genes_gr_prom[i]) 
  cpg_bv <- t(assay(m[cpg_gr$cgid,]))
  colnames(cpg_bv) <- paste0(cpg_gr$location, "_", cpg_gr$cgid)
    #remove columns that are >50% NA
  cpg_betaVal <- cpg_bv %>% 
    .[,colSums(is.na(.)) < 0.5*nrow(.)] %>%
    apply(2, range01) %>%
    data.frame() %>%
    rownames_to_column("tcga_id") %>%
    mutate(tcga_id = substr(tcga_id, 1, 12))
 
  cnvStat <- cnvStatusAtLoci(genes_gr2[i], cnv2) %>% 
    data.frame() %>% 
    dplyr::select(tcga_id, seg_mean) %>%
    mutate(tcga_id=substr(tcga_id,1,12)) %>%
    group_by(tcga_id) %>%
    summarise(seg_mean = mean(seg_mean)) %>%
    #average duplicates
    mutate(seg_mean=scales::rescale(seg_mean,to=c(0, 1)))

  #get the adjacent normal expression data
  exprNml = assay(en)[genes_gr2[i]$ensg,] %>% 
    data.frame() %>% 
    rownames_to_column("tcga_id") %>% 
    mutate(tcga_id = substr(tcga_id, 1,12)) %>%
    rename("expr_nml_txue"=".") %>% as_tibble()
  
  xVars <- mmCatVar %>% 
    left_join(contVar, by="tcga_id") %>%
    left_join(cpg_betaVal, by="tcga_id") %>%
    left_join(cnvStat, by="tcga_id") %>%
    left_join(exprNml, by="tcga_id") %>% 
    dplyr::select(-tcga_id, -X.Intercept.) %>%
    as.matrix() %>%
    fillNAwithColMeans() %>%
    as_tibble() %>%
    select_if(colSums(!is.na(.)) > 0) %>%
    as.matrix()
  
  y = colData(et)[,paste0("cl_", genes_gr2[i]$ensg)]
  lr_res <- logRegElNetLooc(xVars, y, alpha = 0.5, retGlmNt = TRUE)
  
  predProbs <- sapply(1:nrow(xVars), function(x) 
    predict(lr_res, xVars[x,,drop=F], type="response"))
  
  auc_res <- predProbs %>% 
    roc(factor(y)) %>% 
    auc()
  aucResults <- c(aucResults, auc_res)
    
  lr_res_raw <- lr_res %>% 
    coef() %>%
    as.matrix() %>%
    data.frame() %>%
    rownames_to_column(var="varNm")
  
  lr_res_maxBeta <- allVarClass %>%
    sapply(function(x) grep(paste0("^",x), lr_res_raw$varNm)) %>%
    lapply(., function(x) lr_res_raw[x,"s0"][which.max(
      abs(lr_res_raw[x,"s0"]))]) %>%
    lapply(function(x) ifelse(length(x) == 0, NA, x)) %>%
    unlist() %>% 
    data.frame(betaVal=.) %>%
    rownames_to_column(var="varNm") %>%
    mutate(rnk =rank(-abs(betaVal), na.last=T)) %>%
    mutate(rnk=ifelse(is.na(betaVal)," ", ifelse(betaVal == 0, "·", rnk)),
           varNm = factor(varNm, levels=rev(allVarClass), labels=rev(allVarLabel)))
  
  lr_res_maxBeta.l <- c(lr_res_maxBeta.l, list(lr_res_maxBeta))
}
names(lr_res_maxBeta.l) <- genes_gr2$gene_symb
names(aucResults) <- genes_gr2$gene_symb
```

## Figure 3A

Now, generate a matrix and visualize the coefficients.

```
coefMatr <- lr_res_maxBeta.l %>% 
  do.call(rbind, .) %>% 
  rownames_to_column("gene") %>%
  separate("gene", c("gene", "toRm")) %>%
  dplyr::select(-toRm) %>% 
  mutate(betaValCat = cut(.[,"betaVal"], 
          breaks=c(-Inf, -2.5, -1.25, -0.25, -1*10^(-6),
                   1*10^(-6), 0.25, 1.25, 2.5, Inf))) %>%
  replace_na(list(betaValCat="(-1e-06,1e-06]")) %>%
  mutate(gene = factor(gene, levels=genes_gr2$gene_symb))

#plot the coefficient matrix
ggplot(coefMatr, aes(x=gene, y=varNm, fill=betaValCat)) + 
  geom_tile(color="grey") + coord_equal() + theme_classic() + 
  scale_fill_brewer(palette = "RdBu", type="div", 
                    drop=FALSE, direction = -1) + 
  geom_text(aes(label=rnk)) +
  theme(axis.ticks = element_blank(),
        axis.line = element_blank(),
        axis.text = element_text(color="black", size=16),
        axis.text.x = element_text(angle=90, vjust=0.5, hjust=1)) +
  xlab("") + ylab("")
```

```
#plot the AUC values
aucResults.df <- aucResults %>% 
  as.data.frame()%>% 
  rownames_to_column("gene") %>%
  rename(aucVal=".") %>%
  mutate(gene = factor(gene, levels=genes_gr2$gene_symb)) %>%
  mutate(colorsCut = cut(aucVal, c(0,0.5,0.6,0.8,0.9,0.95,0.99,0.999,1))) #c(0,0.6,0.8,0.85,0.9,0.95,1)))

ggplot(aucResults.df, aes(y = as.factor("AUC"), x = gene)) + 
  theme_classic() + 
  geom_tile(data = aucResults.df, color="black", aes(fill = colorsCut)) + 
  scale_fill_brewer(palette = "Purples", drop=F) +
  theme(aspect.ratio=0.15) + 
  geom_text(color = "black", size=12, aes(label = round(aucVal,2))) + 
  theme(axis.text.x = element_blank(), axis.ticks=element_blank(),
        axis.text.y= element_text(size=20, hjust=2), 
        axis.line = element_blank()) + 
  xlab("") + ylab("")
```

## Supplementary Figure 7 (Part 1) - CpG Methylation

Plot all the CpGs at the CBX2 locus

```
class_cbx2 = colData(et) %>% 
  data.frame %>%
  dplyr::select("cl_ENSG00000173894") %>% 
  rownames_to_column(var = "ptId") %>%
  mutate(ptId, ptId = substr(ptId, 1,12))

cbx2CpGs <- methStatusAtLoci(genes_gr2[3], methSE)

cpg_cbx2 <- genes_gr2 %>%
   subset(gene_symb == "CBX2") %>%
   methStatusAtLoci(methSE) %>%
   .$cgid %>%
   m[.,] %>% 
   assay() %>%
   t() %>% 
   data.frame() %>%
   rownames_to_column(var = "ptId") %>%
   mutate(ptId, ptId = substr(ptId, 1,12)) %>%
   left_join(class_cbx2, by="ptId") %>%
   mutate(cl_ENSG00000173894,
          cl_ENSG00000173894=factor(cl_ENSG00000173894)) %>%
   reshape2::melt() %>%
  rename(cpg=variable) %>%
  #order by position
  mutate(cpg=factor(cpg, levels=sort(cbx2CpGs)$cgid)) %>%
  na.omit()
```

```
## Using ptId, cl_ENSG00000173894 as id variables
```

```
#examine the differences in group means between the CpG values
cpg_cbx2.s <- spread(cpg_cbx2, cpg, value)
wilcox.res.pvals = c()
for(ea_col in 3:ncol(cpg_cbx2.s)){
  #do a wilcoxon test for differences in means...
  wilcox.res = wilcox.test(cpg_cbx2.s[,ea_col] ~
                             cpg_cbx2.s$cl_ENSG00000173894, cpg_cbx2.s)
  wilcox.res.pvals = c(wilcox.res.pvals, wilcox.res$p.value)
}

wilcox.res.pvals.adj <- p.adjust(wilcox.res.pvals, method="BH")
names(wilcox.res.pvals.adj) <- colnames(cpg_cbx2.s)[3:ncol(cpg_cbx2.s)]
wilcox.res.pvals.adj.star <- sig2star01(wilcox.res.pvals.adj)

ggplot(cpg_cbx2, aes(x = cpg, y = value, fill = cl_ENSG00000173894, color = cl_ENSG00000173894)) +
  theme_classic() + 
  theme(axis.text.x = element_text(angle = 90, size = 8, hjust = 1, vjust = 0.5), aspect.ratio=0.30) +
  geom_boxplot(outlier.alpha = 0.5) + 
  scale_fill_brewer(palette = "Paired", name = "CBX2 Expression", labels=c("Baseline", "Overexpressed")) +
  scale_color_brewer(palette = "Paired") + 
  geom_boxplot(color="black", outlier.shape = NA) + 
  ylab("Beta Value") + ylim(0,1.4) + 
  annotate("text", label = paste0(" ", wilcox.res.pvals.adj.star), 
           x = 1:length(wilcox.res.pvals.adj.star), y = 1.02, 
           color = "black", size = 8, angle = 90, vjust = 0.75) + 
  annotate("text", x = 10, y = 1, label = "* q<0.01\n**q<0.001\n***q<0.0001")
```

## Figure 3B

Plot the most significant CpG from CBX2 and perform post-hoc statistical testing.

```
cg_sml_pval <- names(which(wilcox.res.pvals.adj == min(wilcox.res.pvals.adj)))
cg_sml_pval
```

```
## [1] "cg25270886"
```

```
min(wilcox.res.pvals.adj)
```

```
## [1] 6.107046e-09
```

```
cpg_cbx2_1cg <- cpg_cbx2 %>% filter(cpg == cg_sml_pval) %>% mutate(cl_ENSG00000173894 = ifelse(cl_ENSG00000173894 == 0, "Baseline", "Overexpressed"))

ggplot(cpg_cbx2_1cg, aes(x = cl_ENSG00000173894, y = value, 
                         fill = cl_ENSG00000173894, color = cl_ENSG00000173894)) +
  theme_classic() + 
  geom_jitter(width=0.2, height=0, alpha=0.5, size=4, pch=21, color="black") + 
  geom_boxplot(fill=NA, outlier.shape = NA) + 
  theme(text = element_text(color="black", size=20), legend.position="none") + 
  scale_fill_brewer(palette = "Paired", name = "CBX2 Expression", labels=c("Baseline", "Overexpressed")) +
  scale_color_brewer(palette = "Paired") + 
  ylab("Beta Value") + xlab("")
```

Now, see which have peaks in the vicinity of CBX2 loci. Create a 28 x n binary matrix, indicating whether a peak is present at the CpG site or not. The file “metadata.txt” is provided; ChIP-seq experiments listed in this file can be downloaded from ENCODE GUI (no easy-to-use interface from R unfortunately).

Look at methylation status across CBX2 locus

```
path2ChipSeq = here("raw_data",
                    "2017_04_14_encode_chipSeq_mcf7",
                    "columnCut1237") 

encode_chipSeq_fn = list.files(path2ChipSeq)

#remove any file that doesn't end in .bed
encode_chipSeq_fn = encode_chipSeq_fn[
                       substr(encode_chipSeq_fn,
                       nchar(encode_chipSeq_fn[2]) - 3,
                       nchar(encode_chipSeq_fn[2])) == ".bed"
                       ]
encode_chipSeq_fn.trim = substr(encode_chipSeq_fn, 5, 15)
                                             
############
#read in the metadata file
metadata.mcf7.encode = read_delim(file = here("raw_data",
                                              "2017_04_14_encode_chipSeq_mcf7", 
                                              "metadata.txt"), delim = "\t", col_names = TRUE) %>% 
  filter(`File accession` %in% encode_chipSeq_fn.trim) %>% 
  dplyr::select("File accession","Output type","Experiment target")


encode_chipSeq.mcf7.list = GRangesList()

for(i in 1:nrow(metadata.mcf7.encode)){
  encode_chipSeq.list =  import(here("raw_data", "2017_04_14_encode_chipSeq_mcf7", "columnCut1237", paste0("cut_", metadata.mcf7.encode[i,"File accession"], ".bed")), format="BED")
  
  x = as.numeric(encode_chipSeq.list@elementMetadata$name)
  y = rescale(x,c(10,1000))
  y = as.data.frame(matrix(y, ncol=1))
  colnames(y) = "rescaledRange"
  values(encode_chipSeq.list) <- cbind(values(encode_chipSeq.list), rescaledRange = y)  
  encode_chipSeq.mcf7.list[[i]] <- encode_chipSeq.list
}
names(encode_chipSeq.mcf7.list) <- metadata.mcf7.encode$`Experiment target` #encode_chipSeq_fn.trim
#plot histograms for each of these to make sure there is no severe right skew
```

## Supplementary Figure 7 (Part 2) - ChIP-seq

```
## overlap the CBX2 CpGs with the ENCODE ChIP-seq data from MCF7

encode_chipSeq.mcf7.list.sbst <- encode_chipSeq.mcf7.list %>% 
  lapply(findOverlaps, cbx2CpGs)

encode_chipSeq.mcf7.list.sbst.chip <- 
  encode_chipSeq.mcf7.list.sbst %>% 
  .[lapply(.,length) != 0] %>% 
  lapply(data.frame) %>%
  do.call(rbind.data.frame, .) %>%
  rownames_to_column(var = "protein") %>%
  separate(protein, c("protein", "toRm"), sep="-") %>%
  dplyr::select(-toRm) %>% 
  cbind(.,as.matrix(cbx2CpGs[.$subjectHits]@elementMetadata)) %>%
  as_tibble() %>%
  mutate(cgid = factor(cgid, levels = sort(cbx2CpGs)$cgid))

#reorder the levels of the rows
tfs <- c("JUND", "SIN3A", "MAZ","CTBP1")
enh <- c("H3K27ac", "H3K4me1")
prom <- c("H3K4me2", "H3K4me3")
repr <- "H3K9ac"
elong <- "H4K20me1"
othHist <- c("H2AFZ", "H3F3A")

orderChip <- rev(c(othHist, enh, prom, repr, elong, tfs))
encode_chipSeq.mcf7.list.sbst.chip <- encode_chipSeq.mcf7.list.sbst.chip %>% mutate(protein = factor(protein, levels = orderChip))

ggplot(encode_chipSeq.mcf7.list.sbst.chip, aes(x = cgid, y = protein)) + theme_classic() + theme(axis.text.x = element_text(angle = 90, size = 8, hjust = 1, vjust = 0.5)) + coord_fixed(ratio = 1) +  geom_tile(color = "lightgrey",width=0.7, height=0.7) + xlab("") + ylab("") + scale_fill_manual(values= c("white", "black"))
```

## Figure 4 – Survival analysis

Survival analysis using CBX2 data from 110 patients as well as from entire TCGA dataset.

Start by looking at 110 patients.

```
i=3
cbx2_bound <- mmp2 %>% 
  rownames_to_column(var="geneId") %>% 
  filter(geneId == genes_gr2[i]$gene_symb) %>%
  dplyr::select(starts_with("tMu")) %>%
  as.numeric() %>% mean()

e_cbx2 <- et[genes_gr2[i]$ensg,substr(colnames(et),14,15) == "01"]

colData(e_cbx2)$cbx2Class <- ifelse(as.numeric(assay(e_cbx2)) > cbx2_bound, 1, 0)

days_last_fu <- ifelse(is.na(colData(e_cbx2)$days_to_death), colData(e_cbx2)$days_to_last_follow_up,colData(e_cbx2)$days_to_death)
days_last_fu <- ifelse(days_last_fu > 365.25 * 5, 365.25 * 5, days_last_fu)

vital_stat <- ifelse(colData(e_cbx2)$vital_status == "alive", 0, 1)
vital_stat <- ifelse(days_last_fu >= 365.25 * 5, 0, vital_stat)

colData(e_cbx2)$days_last_fu_5yr <- days_last_fu
colData(e_cbx2)$years_last_fu_5yr <- days_last_fu/365.25

colData(e_cbx2)$vital_stat_5yr <- vital_stat
e_cbx2 <- e_cbx2[,!is.na(e_cbx2$vital_stat_5yr)]
e_cbx2 <- e_cbx2[,!is.na(e_cbx2$years_last_fu_5yr)]

fit <- survfit(Surv(e_cbx2$years_last_fu_5yr, as.numeric(e_cbx2$vital_stat_5yr)) ~ as.factor(cbx2Class), data = colData(e_cbx2))

fit.stat <- survival::survdiff(Surv(e_cbx2$years_last_fu_5yr, as.numeric(e_cbx2$vital_stat_5yr)) ~ as.factor(e_cbx2$cbx2Class))

pval <- pvalFxn(fit.stat)

group1Deaths <- sum(as.numeric(e_cbx2$vital_stat_5yr[e_cbx2$cbx2Class == 0]))
group2Deaths <- sum(as.numeric(e_cbx2$vital_stat_5yr[e_cbx2$cbx2Class == 1]))

#all patients
GGally::ggsurv(fit, back.white = T)  + 
  xlab("Time (Years)") + ylab("Prob. of Survival") + 
  ggtitle(paste(genes_gr2[i]$gene_symb, ": K-M", "| Lg-Rk p:", round(pval,3),
                "\n",  "G1:", group1Deaths,"/", fit$n[1],"died |", "G2:",
                group2Deaths, "/", fit.stat$n[2], "died",sep=" ")) +
  theme(legend.title=element_blank(), 
        axis.text = element_text(size=18, color = "black"), 
        text = element_text(size=17)) + 
  scale_color_brewer(palette = "Paired", direction = 1) + 
  ylim(c(0,1))
```

```
## Loading required package: scales
```

```
## Warning: package 'scales' was built under R version 3.4.4
```

```
## 
## Attaching package: 'scales'
```

```
## The following object is masked _by_ '.GlobalEnv':
## 
##     rescale
```

```
## The following object is masked from 'package:purrr':
## 
##     discard
```

```
## The following object is masked from 'package:readr':
## 
##     col_factor
```

```
## Scale for 'colour' is already present. Adding another scale for
## 'colour', which will replace the existing scale.
```

```
#ggsave(filename="output/survival_110_cbx2.png")
```

Now look at all patients in tcga

```
e_cbx2_all <- e[genes_gr2[i]$ensg,substr(colnames(e),14,15) == "01"]
dim(e_cbx2_all)
```

```
## [1]    1 1086
```

```
#test <- e[genes_gr2[3]$ensg,substr(colnames(e),14,15) == "01"]

colData(e_cbx2_all)$cbx2Class <- ifelse(as.numeric(assay(e_cbx2_all)) > cbx2_bound, 1, 0)

days_last_fu <- ifelse(is.na(colData(e_cbx2_all)$days_to_death), colData(e_cbx2_all)$days_to_last_follow_up,colData(e_cbx2_all)$days_to_death)
days_last_fu <- ifelse(days_last_fu > 365.25 * 5, 365.25 * 5, days_last_fu)

vital_stat <- ifelse(colData(e_cbx2_all)$vital_status == "alive", 0, 1)
vital_stat <- ifelse(days_last_fu >= 365.25 * 5, 0, vital_stat)

colData(e_cbx2_all)$days_last_fu_5yr <- days_last_fu
colData(e_cbx2_all)$years_last_fu_5yr <- days_last_fu/365.25

colData(e_cbx2_all)$vital_stat_5yr <- vital_stat
e_cbx2_all <- e_cbx2_all[,!is.na(e_cbx2_all$vital_stat_5yr)] #remove pt w/o surv data
dim(e_cbx2_all)
```

```
## [1]    1 1084
```

```
e_cbx2_all <- e_cbx2_all[,!is.na(e_cbx2_all$years_last_fu_5yr)] #remove pt w/o surv data
dim(e_cbx2_all)
```

```
## [1]    1 1084
```

```
fit <- survfit(Surv(e_cbx2_all$years_last_fu_5yr, as.numeric(e_cbx2_all$vital_stat_5yr)) ~ as.factor(cbx2Class), data = colData(e_cbx2_all))

fit.stat <- survival::survdiff(Surv(e_cbx2_all$years_last_fu_5yr, as.numeric(e_cbx2_all$vital_stat_5yr)) ~ as.factor(e_cbx2_all$cbx2Class))

pval2 = pvalFxn(fit.stat)
print(pval2)
```

```
## [1] 0.01695547
```

```
group1Deaths <- sum(as.numeric(e_cbx2_all$vital_stat_5yr[e_cbx2_all$cbx2Class == 0]))
group2Deaths <- sum(as.numeric(e_cbx2_all$vital_stat_5yr[e_cbx2_all$cbx2Class == 1]))

#all patients
GGally::ggsurv(fit, back.white = T) + 
  xlab("Time (Years)") + 
  ylab("Prob. of Survival") + 
  ggtitle(paste(genes_gr2[i]$gene_symb, ": K-M", "| Lg-Rk p:", round(pval2,3), 
                "\n",  "G1:", group1Deaths,"/", fit$n[1],"died |", "G2:",
                group2Deaths, "/", fit.stat$n[2], "died",sep=" ")) + 
  theme(legend.title=element_blank(), 
        axis.text = element_text(size=18, color = "black"), 
        text = element_text(size=17)) + 
  scale_color_brewer(palette = "Paired", direction = 1) + ylim(c(0,1))
```

```
## Scale for 'colour' is already present. Adding another scale for
## 'colour', which will replace the existing scale.
```

```
#ggsave(filename="output/survival_all_cbx2.png")
#ggsave(filename="output/survival_all_cbx2.png", width = 6, height = 5, units = "in")

p.adjust(c(pval, pval2), "BH")
```

```
## [1] 0.07631043 0.03391094
```

Show histogram of distribution of CBX2 expression values across all patients with cutoff

```
expr_dat_cbx2_all <- assay(e_cbx2_all["ENSG00000173894",]) %>% 
  t() %>% 
  as.data.frame %>% 
  rownames_to_column("tcga_id") %>% 
  mutate(cbx2Class = as.factor(ifelse(ENSG00000173894 > cbx2_bound, 1,0)))

table(expr_dat_cbx2_all$cbx2Class)
```

```
## 
##   0   1 
## 635 449
```

```
ggplot(expr_dat_cbx2_all, aes(x=ENSG00000173894, fill=cbx2Class)) + 
geom_histogram(binwidth = 0.2) + scale_fill_brewer(palette = "Paired", name = "CBX2 Expression", labels=c("Baseline", "Overexpressed")) + theme_classic() + theme(text = element_text(color="black", size=20)) + ylab("Frequency") + xlab("") + geom_vline(xintercept = cbx2_bound) + xlab(paste0("CBX2 mRNA Expression in Tumors"))# (N=",nrow(e_cbx2), ")"))
```

```
#ggsave(filename = "output/cbx2_expr_all_tum_classif.png", width=7, height=5)
```

## Supplementary Figure 8

Plotting relationship between molecular subtype and cbx2 expression

```
subtypeDf <- colData(et) %>% 
  data.frame() %>% 
  dplyr::select(cl_ENSG00000173894, aims_subtypes) %>%
  mutate(aims_subtypes = factor(aims_subtypes, levels=c("Normal", "LumA", "LumB", "Her2", "Basal"))) %>%
  mutate(cl_ENSG00000173894 = factor(cl_ENSG00000173894))

#simplify
ggplot(subtypeDf, aes(x=aims_subtypes, fill=cl_ENSG00000173894, color=cl_ENSG00000173894)) + geom_histogram(stat="count") + scale_color_brewer(palette = "Paired") + scale_fill_brewer(palette = "Paired") + xlab("")  + theme_classic() + scale_y_continuous(expand = c(0,0)) + ylab("Frequency") + theme(text = element_text(size = 16, color="black"))
```

```
## Warning: Ignoring unknown parameters: binwidth, bins, pad
```

```
## calculate statistics -- fishers exact for each group
subtypes <- unique(subtypeDf$aims_subtypes)
pvalFishStore <- c()
for(st in subtypes){
  pvalFish <- subtypeDf %>% 
    mutate(subtypeBinary = ifelse(aims_subtypes == st, 1,0)) %>% 
    fisher.test(x=.$cl_ENSG00000173894, y = .$subtypeBinary) %>%
    .$p.value
  pvalFishStore <- c(pvalFishStore, pvalFish)
}
names(pvalFishStore) <- subtypes
p.adjust(pvalFishStore, method="BH")
```

```
##        Basal         LumA         LumB         Her2       Normal 
## 8.076490e-04 1.765789e-05 1.000000e+00 1.601974e-03 5.005285e-02
```

```
## percent high
tbl_perc <- table(subtypeDf$cl_ENSG00000173894, subtypeDf$aims_subtypes)
round(tbl_perc[2,] / colSums(tbl_perc), 2)
```

```
## Normal   LumA   LumB   Her2  Basal 
##   0.00   0.11   0.40   0.72   0.82
```

## Supplementary Table 1, Supplementary Figure 4

Benchmarking oncomix against mCOPA.

```
## Prepare the data for mCOPA
if(!file.exists(here("processed_data", "mCopa", "exprMatrMcopa.txt"))){
  exprMatrMcopa = cbind(assay(en), assay(et))
  exprMatrMcopa = ifelse(exprMatrMcopa< 1*10^(-6), 0 , exprMatrMcopa)
  write.table(exprMatrMcopa, here("processed_data", "mCOPA", "exprMatrMcopa.txt"), sep="\t", quote = F)
}
```

Perl script below. Run this on the command line

```
module load perl/5.22.1/gcc.4.4.7 

perl mCOPA.pl exprMatrMcopa.txt out/mCOPA_Up90.txt out/mCOPA_Down10.txt 90 10 110
cut -f1 out/mCOPA_Up90.txt > out/mCOPA_Up90_gene.txt
cat out/mCOPA_Up90.txt | rev | cut -d$'\t' -f1| rev > out/mCOPA_Up90_gene_log2fc.txt 
paste out/mCOPA_Up90_gene.txt out/mCOPA_Up90_gene_log2fc.txt > out/mCOPA_Up90_2col.txt

perl mCOPA.pl exprMatrMcopa.txt out/mCOPA_Up80.txt out/mCOPA_Down20.txt 80 20 110
cut -f1 out/mCOPA_Up80.txt > out/mCOPA_Up80_gene.txt
cat out/mCOPA_Up80.txt | rev | cut -d$'\t' -f1| rev > out/mCOPA_Up80_gene_log2fc.txt 
paste out/mCOPA_Up80_gene.txt out/mCOPA_Up80_gene_log2fc.txt > out/mCOPA_Up80_2col.txt

perl mCOPA.pl exprMatrMcopa.txt out/mCOPA_Up70.txt out/mCOPA_Down30.txt 70 30 110
cut -f1 out/mCOPA_Up70.txt > out/mCOPA_Up70_gene.txt
cat out/mCOPA_Up70.txt | rev | cut -d$'\t' -f1| rev > out/mCOPA_Up70_gene_log2fc.txt 
paste out/mCOPA_Up70_gene.txt out/mCOPA_Up70_gene_log2fc.txt > out/mCOPA_Up70_2col.txt
```

Read in the output from mCOPA.

```
mcopa_70 = read.table(here("processed_data", "mCopa", "out","mCOPA_Up70_2col.txt"), header = T, stringsAsFactors = F) %>% 
  rename_("genes" = names(.)[1], "log2FC" = names(.)[2]) %>%
  mutate(percentile = "70") %>% as_tibble()

mcopa_80 = read.table(here("processed_data", "mCopa", "out", "mCOPA_Up80_2col.txt"), header = T, stringsAsFactors = F) %>% 
  rename_("genes" = names(.)[1], "log2FC" = names(.)[2]) %>%
  mutate(percentile = "80") %>% as_tibble()

mcopa_90 = read.table(here("processed_data", "mCopa", "out", "mCOPA_Up90_2col.txt"), header = T, stringsAsFactors = F) %>% 
  rename_("genes" = names(.)[1], "log2FC" = names(.)[2]) %>%
  mutate(percentile = "90") %>% as_tibble()

mcopa <- bind_rows(mcopa_70, mcopa_80, mcopa_90) %>% 
  group_by(percentile) %>% 
  arrange(desc(log2FC), .by_group = TRUE) %>%
  mutate(id = row_number())

## how many genes total were ranked between the 3 mcopa runs
mcopa %>% ungroup() %>% select(genes) %>% distinct()
```

```
## # A tibble: 2,152 x 1
##    genes          
##    <chr>          
##  1 ENSG00000110925
##  2 ENSG00000196517
##  3 ENSG00000127527
##  4 ENSG00000049656
##  5 ENSG00000162736
##  6 ENSG00000125445
##  7 ENSG00000160948
##  8 ENSG00000123131
##  9 ENSG00000132294
## 10 ENSG00000178796
## # ... with 2,142 more rows
```

```
## where do top5 oncomix genes fall in mCOPA rankings
mcopa %>% filter(genes %in% genes_gr2$ensg)
```

```
## # A tibble: 3 x 4
## # Groups:   percentile [3]
##   genes           log2FC percentile    id
##   <chr>            <dbl> <chr>      <int>
## 1 ENSG00000089692   2.34 70          1818
## 2 ENSG00000089692   2.48 80          1155
## 3 ENSG00000089692   2.02 90          1076
```

```
## plot the distribution of the top5 mcopa genes
mcopa_top5 <- mcopa %>% top_n(5, log2FC) %>% left_join(ensg_to_prot, by=c("genes"= "Gene.stable.ID"))


gglayers1 = list(
    theme(axis.text.x = element_text(size=24), plot.title=element_text(size=26)),
  xlim(0,12.5), xlab(""), ylab("")
)

#remove curved lines
for(i in 1:nrow(mcopa_top5)){
  gglayers1[["title"]] <- ggtitle(mcopa_top5$HGNC.symbol[i])
  gene <- as.character(mcopa[i,"genes"])
  plotGeneHistNoProbCrv(mmParams = mmp, exprNml=assay(en), exprTum=assay(et), isof=gene, gglayers1)
  ggsave(here("output", "mcopa", paste0(gene,"_", mcopa_top5[i,"percentile"], "_",mcopa_top5[i,"log2FC"],  ".png")), width=4, height=4, units="in")
}
```

Benchmarking oncomix against limma. Perform a 2 sample t-test between tumor and normal samples.

```
classif <- c(rep(0, ncol(en)), rep(1, ncol(et))) 
dsgn <- model.matrix(~ classif + 
                       c(colData(en)[,"seqPlate"], colData(et)[,"seqPlate"]) +
                       c(colData(en)[,"hosp"], colData(et)[,"hosp"]))

#where do our 5 genes fall inthe limma rank among upregulated genes?

genesUpInTum <- returnDeGenesLimma(cbind(assay(en), assay(et)), 
                           dsgn, colOfInt = 2, retAll=T) %>% 
              rownames_to_column("genes") %>% 
              arrange(desc(-adj.P.Val)) %>% 
              as_tibble() %>% filter(logFC > 0) %>%
              mutate(rank = row_number())
```

```
## Coefficients not estimable: c(colData(en)[, "hosp"], colData(et)[, "hosp"])GI
```

```
dim(genesUpInTum)
```

```
## [1] 7388    8
```

```
#now we show where our genes fall in the limma ranks
limma_oncmx_olp <- genesUpInTum %>% 
              filter(genes %in% genes_gr2$ensg)
limma_oncmx_olp
```

```
## # A tibble: 5 x 8
##   genes           logFC AveExpr     t      P.Value   adj.P.Val     B  rank
##   <chr>           <dbl>   <dbl> <dbl>        <dbl>       <dbl> <dbl> <int>
## 1 ENSG00000155886 1.67    0.907 14.6      4.40e⁻³³    1.13e⁻³¹ 64.5    149
## 2 ENSG00000083782 2.41    1.33  12.4      1.67e⁻²⁶    2.41e⁻²⁵ 49.4    279
## 3 ENSG00000173894 1.58    2.77   9.36     1.86e⁻¹⁷    1.21e⁻¹⁶ 28.7    756
## 4 ENSG00000184613 1.54    2.94   5.75     3.32e⁻ ⁸    9.34e⁻ ⁸  7.75  2264
## 5 ENSG00000089692 0.683   1.94   4.59     7.92e⁻ ⁶    1.72e⁻ ⁵  2.44  3077
```

```
## visualize the distribution of the top 5 genes
genesUpInTumTop5 <- genesUpInTum %>% 
  top_n(5, -adj.P.Val) %>% 
  left_join(ensg_to_prot, by=c("genes"= "Gene.stable.ID"))

gglayers1 = list(
    theme(axis.text.x = element_text(size=24), plot.title=element_text(size=26)),
  xlim(0,12.5), xlab(""), ylab("")
)

for(i in 1:nrow(genesUpInTumTop5)){
  gene = as.character(genesUpInTumTop5[i,"genes"])
  gglayers1[["title"]] <- ggtitle(genesUpInTumTop5$HGNC.symbol[i])
  plotGeneHistNoProbCrv(mmParams = mmp, exprNml=assay(en), exprTum=assay(et), isof=gene, gglayers1)
  ggsave(here("output", "limma", paste0(gene,"_", genesUpInTumTop5[i,"rank"], "_",mcopa[i,"log2FC"],  ".png")), width=4, height=4, units="in")
}
```

## Supplementary Figure 1

Look at the overlap between known oncogenes and our oncomix scores.

Read in a list of established oncogenes and tumor suppressors from COSMIC, v83,, downloaded Jan. 20, 2018.

```
cosDat <- read_csv(file=here("raw_data", "2018_01_20_cosmic", "2018_01_20_Census_all.csv"))
```

```
## Parsed with column specification:
## cols(
##   .default = col_character(),
##   `Entrez GeneId` = col_integer(),
##   Tier = col_integer()
## )
```

```
## See spec(...) for full column specifications.
```

```
names(cosDat) <- gsub(" ", "_", names(cosDat))

cosDatOncg <- cosDat %>% 
    filter(Tier == 1) %>%
    filter(str_detect(Role_in_Cancer, "oncogene")) %>%
    filter(!str_detect(Role_in_Cancer, "TSG")) %>%
    rename("entrezgene" = Entrez_GeneId)

if(file.exists(here("raw_data", "2018_01_20_ensg_entrz","entrez_ensg.rds"))){
  entrez_ensg <- readRDS(here("raw_data", "2018_01_20_ensg_entrz","entrez_ensg.rds"))
} else {
    ensembl = useMart("ensembl",dataset="hsapiens_gene_ensembl")
    attributes = listAttributes(ensembl)
    entrez_ensg <- getBM(mart=ensembl, 
                         attributes=c("ensembl_gene_id", "entrezgene"),
                         values = "entrezgene")
    saveRDS(entrez_ensg, file=here("raw_data", "2018_01_20_ensg_entrz","entrez_ensg.rds"))
} 
#map the gene symbols to ENSG
cosDatOncgE <- left_join(cosDatOncg, entrez_ensg, by="entrezgene")

#look at distribution of oncomix scores in oncogenes
mmpOnc <- mmp %>% 
  rownames_to_column("ensembl_gene_id") %>%
  mutate(rank = row_number()) %>%
    as_tibble() %>%
  filter(ensembl_gene_id %in% cosDatOncgE$ensembl_gene_id) %>%
  left_join(cosDatOncgE, by="ensembl_gene_id") 

mmpOncFilt <- mmpOnc %>% filter(SI > .99, score > 0)

mmpLab <- mmp
idx2replace <- sapply(mmpOncFilt$ensembl_gene_id, grep, rownames(mmpLab))
rownames(mmpLab)[idx2replace] <- mmpOncFilt$Gene_Symbol

#show the scores for the top oncogene candidates identified by oncomix
mmpLab %>% rownames_to_column("gene_id") %>%
  filter(gene_id %in% mmpOncFilt$Gene_Symbol)
```

```
##    gene_id      nMu1      nMu2       nVar       nPi1      tMu1      tMu2
## 1     LMO1 0.3516258 0.4028593 0.20313801 0.53775722 0.3235132  2.999381
## 2   HOXA13 0.4534165 1.0264406 0.04644180 0.87489764 0.3027152  3.047879
## 3     TAL2 0.4432727 0.5360506 0.24245524 0.58175281 0.5873557  2.750804
## 4     SOX2 0.2122669 1.5307940 0.05508013 0.87160405 0.3203616  3.469381
## 5   HOXD13 0.1895123 1.0034185 0.04627831 0.86168925 0.2894383  2.534061
## 6  WHSC1L1 3.9747188 5.1202297 0.10121847 0.09644618 4.8630534  7.758587
## 7    PSIP1 4.8319195 5.6588232 0.08969016 0.07619842 5.0354927  7.591011
## 8    KAT6A 4.0452877 5.4666472 0.11583664 0.12132554 5.0932465  7.419123
## 9     STIL 1.6322391 2.1144874 0.26739375 0.57626590 2.7396512  4.071955
## 10   ERBB2 4.1760021 6.7871609 0.47168226 0.16744898 6.8321347 10.960164
## 11   SALL4 0.3495496 0.8467502 0.03093821 0.87897864 0.9983323  1.974797
## 12   CCNE1 1.6219497 2.6644384 0.11354100 0.94473181 2.6809223  4.683597
##          tVar      tPi1 deltaMu2    deltaMu1        SI      score
## 1  0.15028154 0.9266807 2.596522 -0.02811254 0.9909091 2.25056731
## 2  0.11748177 0.9361485 2.021438 -0.15070126 1.0000000 2.00821565
## 3  0.23865529 0.8704420 2.214753  0.14408304 0.9909091 1.57510925
## 4  0.26919040 0.8095887 1.938587  0.10809474 0.9909091 1.49252920
## 5  0.09145895 0.9727264 1.530643  0.09992598 0.9909091 1.28122495
## 6  0.43856787 0.9413129 2.638357  0.88833466 1.0000000 1.21023628
## 7  0.49484368 0.9659080 1.932188  0.20357320 0.9909091 1.13367977
## 8  0.40490233 0.9673643 1.952476  1.04795879 1.0000000 0.38377830
## 9  0.32710061 0.5451692 1.957468  1.10741207 0.9909091 0.25323820
## 10 0.83584378 0.9017369 4.173003  2.65613257 0.9909091 0.20744110
## 11 0.25451795 0.5066894 1.128047  0.64878279 1.0000000 0.19380793
## 12 0.75376019 0.8304956 2.019159  1.05897267 1.0000000 0.09288494
```

```
#plot the distributions of these oncogenes

gglayers2 = list(
    theme(axis.text.x = element_text(size=24), plot.title=element_text(size=26)),
  xlim(0,12.5), xlab(""), ylab("")
)

for(i in 1:length(mmpOncFilt$Gene_Symbol)){
  gglayers2[["title"]] <- ggtitle(paste0(mmpOncFilt$Gene_Symbol[i], " Score: ", round(mmpOncFilt$score[i],2)))
  #print(range(assay(en)[mmpOncFilt$ensembl_gene_id[i],], assay(et)[mmpOncFilt$ensembl_gene_id[i],]))
  #print(mmpOncFilt$Gene_Symbol[i])
  plotGeneHist(mmp, assay(en), assay(et), mmpOncFilt$ensembl_gene_id[i], gglayers2)
  ggsave(filename = here("output", paste0("known_oncog_",mmpOncFilt$Gene_Symbol[i], "_histog.png")), width = 5, height=5, units = "in")
}
```

Create a plot comparing the selectivity index of known oncogene candidates vs all genes

```
## is there enrichment of oncomix score among oncogenes??
ggplot(mmpOnc, aes(x=SI)) +
  geom_histogram(binwidth = 0.02, color="grey", alpha=alph, aes(fill=SI>0.99)) + 
  theme_classic() + scale_fill_manual(values=reds[c(3,7)]) + scale_y_continuous(expand = c(0, 0))  + xlab("Selectivity Index") + ylab("Frequency") + theme(text=element_text(size=20, color="black"), legend.position="none")
```

```
ggplot(mmpOnc, aes(x=score, y=..density.., fill=SI > 0.99)) +
  geom_histogram(data=subset(mmpOnc, SI<0.99), aes(x=score, y=..density.., fill=SI > 0.99), binwidth = 0.1, color="grey", alpha=alph) + 
  geom_histogram(data=subset(mmpOnc, SI>0.99), aes(x=score, y=..density.., fill=SI > 0.99), binwidth = 0.1, color="grey", alpha=alph) +
  theme_classic() + scale_fill_manual(values=reds[c(3,7)]) + scale_y_continuous(expand = c(0, 0)) + theme(text=element_text(size=20, color="black"), legend.position="none") + ylab("Density") + xlab("Oncomix Score")
```

## Supplementary Figure 3

Expression of oncogenes from the Cancer Gene Census in normal adult tissue from Gtex.

```
gtexDat <- read.gct(here("raw_data", "2016_10_05_gTex_tissue", "GTEx_Analysis_2016-01-15_v7_RNASeQCv1.1.8_gene_median_tpm.gct"))

gtexDat2 <- gtexDat %>% 
  as.data.frame() %>% 
  rownames_to_column("ensg") %>% 
  as_tibble() %>% 
  clean_names() %>% 
  mutate(ensg = substr(ensg, 1,15)) 

gtexDat2.m <- gtexDat2 %>% 
  filter(ensg %in% mmpOncFilt$ensembl_gene_id) %>%
  gather(key="tissue", value="exprlvl", 2:ncol(.)) %>%
  mutate(exprlvlbin = ifelse(exprlvl>1, 1, 0)) %>%
  left_join(select(mmpOncFilt, ensembl_gene_id, Gene_Symbol), by=c("ensg" = "ensembl_gene_id")) %>%
  mutate(tissue = factor(tissue, levels=sort(unique(tissue), decreasing=TRUE)))

#cluster these samples
gtexDat2.s <- gtexDat2.m %>% select(Gene_Symbol, exprlvlbin, tissue) %>% spread(key = tissue, value=exprlvlbin) %>% as.data.frame() 
rownames(gtexDat2.s) <- gtexDat2.s[,"Gene_Symbol"]
gtexDat2.s.m <- as.matrix(gtexDat2.s %>% select(-Gene_Symbol))

d <- dist(gtexDat2.s.m, method = "euclidean")
gtex_clust <- hclust(d, method = "complete" )
plot(gtex_clust, cex = 0.6, hang = -1, lwd = 2)
```

```
gtexDat2.m2 <- gtexDat2.m %>% 
  mutate(Gene_Symbol = factor(Gene_Symbol, levels=gtex_clust$labels[gtex_clust$order]),
  exprlvlbin = factor(exprlvlbin, levels=c(0,1), labels=c("< 1", "\u2265 1")))

ggplot(gtexDat2.m2, aes(x = Gene_Symbol, y = tissue, fill = exprlvlbin)) + 
  geom_tile(color = "white", size = 0.4) +
  scale_fill_brewer(direction = 1, name="TPM") + 
  theme_classic() + 
    theme(axis.ticks = element_blank(), 
          axis.line = element_blank(),
          axis.text.x = element_text(angle = 90, hjust = 1, vjust=0.5)) + 
  coord_fixed(ratio = 1) + xlab("") + ylab("")
```

Supplementary Figure 3A

Percentage of GTEx tissues expressing the CGC gene

```
gtexDat3 <- gtexDat2 %>%  
    filter(ensg %in% mmpOnc$ensembl_gene_id) %>%
  gather(key="tissue", value="exprlvl", 2:ncol(.)) %>%
  mutate(exprlvlbin = ifelse(exprlvl>1, 1, 0))

gtexDat4 <- gtexDat3 %>% 
  group_by(ensg, exprlvlbin) %>%
  summarise (n = n()) %>%
  mutate(freq = n / sum(n))

gtexDat5 <- gtexDat4 %>% 
  ungroup() %>%
  ## make implicit missing value explicit
  complete(ensg, exprlvlbin, fill = list(n = 0, freq = 0)) 

genes_to_label <- c("CCNE1", "SALL4", "TAL2", "HOXA13", "HOXD13", "SOX2", "ERBB2")

gtexDat6 <- gtexDat5 %>% 
  left_join(select(mmpOncFilt, ensembl_gene_id, Gene_Symbol), by=c("ensg" = "ensembl_gene_id")) %>%
  mutate(idByOm = ifelse(is.na(Gene_Symbol), "No", "Yes"),
         Gene_Symbol_label = ifelse(Gene_Symbol %in% genes_to_label,
                                    Gene_Symbol, "")) %>%
  filter(exprlvlbin == 1)
  

ggplot(gtexDat6, aes(x=idByOm, y=freq)) + 
  geom_violin() +
  geom_jitter(width = 0.2, height=0, pch=21, alpha = 0.45, aes(fill=idByOm)) + 
  theme_classic() + 
  theme(text = element_text(size=20, color="black")) +
  scale_fill_manual(values=c("white", "purple"), guide=FALSE) +
  geom_text_repel(size=4, aes(label=Gene_Symbol_label)) +
  xlab("Identified by oncomix") +
  ylab("% of 53 GTEX tissue expressing the gene")
```

Supplementary Figure 3A

see whether the genes identified by oncomix are expressed in fewer tissues than the ones not identified by oncomix

```
gr1 <- gtexDat6 %>% filter(idByOm == "No") %>% pull(freq)
gr2 <- gtexDat6 %>% filter(idByOm == "Yes") %>% pull(freq)

t.test(gr1, gr2, alternative="two.sided")
```

```
## 
##  Welch Two Sample t-test
## 
## data:  gr1 and gr2
## t = 2.68, df = 11.601, p-value = 0.02055
## alternative hypothesis: true difference in means is not equal to 0
## 95 percent confidence interval:
##  0.05922785 0.58494771
## sample estimates:
## mean of x mean of y 
## 0.8598236 0.5377358
```

## Supplementary Figure 10 - Mutations and CBX2 overexpression

Now, load the breast cancer somatic mutation data from TCGA.

```
if(file.exists(here("raw_data", "2018_01_20_somMut", "tcga-mut-mutect-brca.rds"))){
  mut <- readRDS(here("raw_data", "2018_01_20_somMut", "tcga-mut-mutect-brca.rds"))
} else {
  mutq <- GDCquery_Maf("BRCA", pipelines = "mutect")
  #saveRDS(mutq, file=here("raw_data", "2018_01_20_somMut", "tcga-mut-mutect-brca.rds"))
}
mut.hiImpct <- mut %>%
  filter(IMPACT %in% "HIGH" & Entrez_Gene_Id %in% cosDat$Entrez_GeneId)
top50mutGenes <- sort(names(head(sort(table(mut.hiImpct$Hugo_Symbol),decreasing = TRUE), 25)))
mut.hiImpct.top50 <- mut.hiImpct[mut.hiImpct$Hugo_Symbol %in% top50mutGenes,]
mut.hiImpct.top50.ptid <- names(table(mut.hiImpct.top50$Tumor_Sample_Barcode))

mutMatr <- matrix(0,ncol=length(mut.hiImpct.top50.ptid), nrow=length(mut.hiImpct.top50))

#which patients are associated with which gene mutations
#this is a list of patient TCGA ids
ptIdGeneMut <- sapply(top50mutGenes, function(x) unname(mut.hiImpct.top50[mut.hiImpct.top50$Hugo_Symbol %in% x,"Tumor_Sample_Barcode"]))

ptIdGeneMut.l <- lapply(ptIdGeneMut, function(x) mut.hiImpct.top50.ptid %in% x )
ptIdGeneMut.l.df <- do.call(rbind, ptIdGeneMut.l)
colnames(ptIdGeneMut.l.df) <- mut.hiImpct.top50.ptid
ptIdGeneMut.l.df <- ifelse(ptIdGeneMut.l.df, 1, 0)

mutDat <- SummarizedExperiment(assays=list(mutation = as.matrix(ptIdGeneMut.l.df)))
oncomix_gene_class <- dplyr::select(data.frame(colData(et)), starts_with("cl_"))
ptInCommon <- intersect(substr(rownames(oncomix_gene_class), 1,12), substr(colnames(assay(mutDat)), 1,12))

#create a histogram of mutation frequencies
mutFreq <- data.frame(freq=sort(rowSums(assay(mutDat[,sapply(ptInCommon, function(x) grep(x, colnames(mutDat)))])),decreasing = T))
mutFreq$gene <- rownames(mutFreq) 
mutFreq$gene <- factor(mutFreq$gene, levels=mutFreq$gene)

ggplot(mutFreq, aes(x=gene, y=freq/length(ptInCommon))) + geom_bar(stat = "identity") + gg__bar + ylab(paste0("Observed Frequency (N=", length(ptInCommon), ")"))
```

```
#expression status
exprStatus.c <- clusterAndReorder(t(oncomix_gene_class)[,substr(rownames(oncomix_gene_class), 1,12) %in% ptInCommon], dimen = "both")
colnames(exprStatus.c) <- substr(colnames(exprStatus.c), 1,12)
exprStatus.c.m <- reshape2::melt(exprStatus.c)

#mutation status
mutDat.c <- clusterAndReorder(assay(mutDat)[,substr(colnames(assay(mutDat)), 1,12) %in% ptInCommon], dimen="both")
#remove rows that are all the same
mutDat.c <- mutDat.c[rowSums(mutDat.c) >= 2,]
colnames(mutDat.c) <- substr(colnames(mutDat.c), 1,12)
mutDat.c.m <- reshape2::melt(mutDat.c)

#bind hormone expression and mutation data together
mutAndExpr <- rbind(mutDat.c, exprStatus.c[,colnames(mutDat.c)])
mutAndExpr.c <- clusterAndReorder(mutAndExpr, dimen = "both")
mutAndExpr.c.m <- reshape2::melt(mutAndExpr.c)

mutAndExpr.c.m$Var2 <- factor(mutAndExpr.c.m$Var2, levels=colnames(mutAndExpr.c))
mutAndExpr.c.m$Var1 <- factor(mutAndExpr.c.m$Var1, levels=rownames(mutAndExpr.c))
mutAndExpr.c.m$Var1 = plyr::mapvalues(mutAndExpr.c.m$Var1, from = paste0("cl_", genes_gr2$ensg), to = genes_gr2$gene_symb)

mutAndExpr.c.m$mut0Expr1 <- ifelse(as.character(mutAndExpr.c.m$Var1) %in% genes_gr2$gene_symb, 1,0)
mutAndExpr.c.m$valMutExpr <- paste0(mutAndExpr.c.m$value, mutAndExpr.c.m$mut0Expr1)
```

Are there associations between oncogene/tumor suppressor mutations and overexpression of oncogenes? Use fisher’s exact test to look at relationship between each NHR and each mutation (50x6 matrix). Our 2 matrices are mutDat.c and exprStatus.c.sub.

```
exprStatus.c.sub <- exprStatus.c[,colnames(mutDat.c)]
rownames(exprStatus.c.sub) <- genes_gr2$gene_symb[sapply(substr(rownames(exprStatus.c.sub), 4,18), grep,genes_gr2$ensg)]

orDat <- array(data = 0, dim = c(nrow(mutDat.c), nrow(exprStatus.c.sub), 2), dimnames = list(muts=rownames(mutDat.c), expr=rownames(exprStatus.c.sub), oddsRat=c("pValue", "OR")))

for(i in 1:nrow(mutDat.c)){
  for(j in 1:nrow(exprStatus.c.sub)){

    d1 <- c(mutDat.c[i,])
    d2 <- c(exprStatus.c.sub[j,])
    if(length(unique(d1)) == 1 | length(unique(d2)) == 1){
      next
    }
    res <- fisher.test(x = d1, d2)
    orDat[i,j,1] <- res$p.value
    orDat[i,j,2] <- res$estimate
  }
}
    

orDat.adj <- matrix(p.adjust(as.vector(as.matrix(orDat[,,1])), method='BH'),ncol=ncol(orDat[,,1]))
orDat <- abind::abind(orDat, adjPval=orDat.adj, along = 3)
orDat.m <- reshape2::melt(orDat)
colnames(orDat.m) <- c("mut", 'nhr', "pval_or", "value")
orDat.m$sigStar <- sig2star(orDat.m$value)
orDat.m$nhr <- factor(orDat.m$nhr, levels=genes_gr$gene_symb)
orDat.m$mut <- factor(orDat.m$mut, levels=rev(mutFreq$gene))

#these are the top 3
subset(orDat.m,pval_or=="adjPval") %>% arrange(value) %>% head(3)
```

```
##      mut   nhr pval_or      value sigStar
## 1   TP53  CBX2 adjPval 0.05284218        
## 2 MAP3K1 NELL2 adjPval 0.54935504        
## 3 MAP3K1  EPYC adjPval 0.54935504
```

```
ggplot(subset(orDat.m,pval_or=="OR"), aes(y=mut, x=nhr)) + 
  geom_tile(aes(fill=log(value,10))) + 
  theme_classic() + 
  theme(text = element_text(size=16), axis.ticks=element_blank(), 
        axis.line = element_blank(), 
        axis.text.x = element_text(angle=90, hjust = 1, vjust=0.5)) + 
  scale_fill_gradient2(name="log10(Odds Ratio)") + 
  geom_text(data=subset(orDat.m,pval_or=="adjPval"), aes(label=sigStar)) + 
  coord_fixed(ratio = 1) + ylab("High-impact mutation") + xlab("")
```

## Figure 5 - CBX2 Growth curve expt.

Objective: Read in, plot, and perform statistical analysis for CBX2 growth curve data

```
paired <- brewer.pal(6, "Paired")

cbx2 <- read_excel(path = here::here("raw_data", "2017_11_26_CBX2_growth_curve", "CBX2.xlsx"), sheet = "Sheet2")

cbx2$day <- substr(cbx2$day, start = 4, stop = 5)

cbx2.cons <- subset(cbx2, expt_numb %in% 4:6)
cbx2.cons.m <- reshape2::melt(cbx2.cons, id.vars=c("replicate_numb", "day", "expt_numb", "floating"))

cbx2.cons.m$day <- as.numeric(cbx2.cons.m$day)
ggplot(subset(cbx2.cons.m, subset = floating == 0 ), aes(x=day, y=log(value,10), color=variable)) + geom_smooth(size=2) +  geom_jitter(position=position_dodge(width=0.4), alpha=0.5, size=4, pch=21, aes(group=variable, fill=variable), color="black", stroke = 2) + theme_classic() + scale_fill_manual(values=paired[c(1,3)]) + scale_color_manual(values=paired[c(1,3)], name="      siRNA") + xlab("Day")+ ylab(bquote('log'[10]* '(cell count)')) + theme(axis.text=element_text(size=16), axis.title=element_text(size=16), plot.title = element_text(size=16)) + ggtitle("siRNA KD: Adherent MCF7, day 3-7") + scale_x_continuous(breaks=c(0,3:7))
```

```
## `geom_smooth()` using method = 'loess' and formula 'y ~ x'
```

```
ggplot(subset(cbx2.cons.m, subset = floating == 1 & day > 0), aes(x=day, y=log(value, 10), color=variable)) + geom_smooth(size=2) +  geom_jitter(position=position_dodge(width=0.4), alpha=0.5, size=4, pch=21, aes(group=variable, fill=variable), color="black", stroke = 2) + theme_classic() + scale_fill_manual(values=paired[c(1,3)]) + scale_color_manual(values=paired[c(1,3)], name="      siRNA") + xlab("Day")+ ylab(bquote('log'[10]* '(cell count)')) +  theme(axis.text=element_text(size=16), axis.title =element_text(size=16), plot.title = element_text(size=16)) + ggtitle("siRNA KD: Floating MCF7, day 3-7")  + scale_x_continuous(breaks=c(0,3:7), limits = c(0,7.1))
```

```
## `geom_smooth()` using method = 'loess' and formula 'y ~ x'
```

now, perform a 3-way ANOVA to check for differences in growth rate as a function of siRNA (either control or CBX2 knockdown)

```
cbx2.cons.m.log2 <- subset(cbx2.cons.m, subset = floating == 0 & day > 0)
cbx2.cons.m.log2.float <- subset(cbx2.cons.m, subset = floating == 1 & day > 0)

cbx2.cons.m.log2.expt5_7 <- cbx2.cons.m.log2 %>% 
  filter(expt_numb %in% 4:6) %>%
  mutate(variable = factor(variable)) %>%
  mutate(expt_numb = factor(expt_numb)) %>%
  mutate(day = factor(day, ordered = TRUE)) %>%
  dplyr::rename(siRNA=variable, cell_counts = value)%>%
  mutate(cell_counts_log = log(cell_counts, 10))

aov_res_log10 <- aov(cell_counts_log ~ siRNA + expt_numb + day, data = cbx2.cons.m.log2.expt5_7)
summary(aov_res_log10)
```

```
##             Df Sum Sq Mean Sq F value   Pr(>F)    
## siRNA        1 0.1387  0.1387  31.855 6.98e-07 ***
## expt_numb    2 0.0804  0.0402   9.235  0.00037 ***
## day          4 1.6211  0.4053  93.105  < 2e-16 ***
## Residuals   52 0.2263  0.0044                     
## ---
## Signif. codes:  0 '***' 0.001 '**' 0.01 '*' 0.05 '.' 0.1 ' ' 1
```

```
#total d.f. is # of samples - 1

## now check the floating cells
cbx2.cons.m.log2.float.expt5_7 <- cbx2.cons.m.log2.float %>% 
  filter(expt_numb %in% 4:6) %>%
  mutate(variable = factor(variable)) %>%
  mutate(expt_numb = factor(expt_numb)) %>%
  mutate(day = factor(day, ordered = TRUE)) %>%
  dplyr::rename(siRNA=variable, cell_counts = value)%>%
  mutate(cell_counts_log = log(cell_counts, 10))

aov_res_log10.f <- aov(cell_counts_log ~ siRNA + expt_numb + day, data = cbx2.cons.m.log2.float.expt5_7)
summary(aov_res_log10.f)
```

```
##             Df Sum Sq Mean Sq F value   Pr(>F)    
## siRNA        1 0.1232  0.1232   3.159   0.0813 .  
## expt_numb    2 0.3797  0.1898   4.870   0.0115 *  
## day          4 2.4220  0.6055  15.531 2.02e-08 ***
## Residuals   52 2.0272  0.0390                     
## ---
## Signif. codes:  0 '***' 0.001 '**' 0.01 '*' 0.05 '.' 0.1 ' ' 1
```

```
#calculate the average fold change between each day/condition

#adherent
fold.change.adh <- cbx2.cons.m.log2.expt5_7 %>% 
  group_by(day, siRNA) %>% 
  select(day, siRNA, cell_counts) %>% 
  summarize(mean_cell_count = mean(cell_counts)) %>%
  spread(siRNA, mean_cell_count) %>%
  mutate(fold_change_ctrl_cbx2 = (si_control -si_CBX2) /si_CBX2)
fold.change.adh
```

```
## # A tibble: 5 x 4
## # Groups:   day [5]
##   day   si_control si_CBX2 fold_change_ctrl_cbx2
##   <ord>      <dbl>   <dbl>                 <dbl>
## 1 3         105583   91833                 0.150
## 2 4         203000  156250                 0.299
## 3 5         257583  206333                 0.248
## 4 6         280417  232833                 0.204
## 5 7         354167  250500                 0.414
```

```
fold.change.adh.overall <- fold.change.adh %>% ungroup() %>% 
  summarise(mean_overall_fc = mean(fold_change_ctrl_cbx2))
fold.change.adh.overall
```

```
## # A tibble: 1 x 1
##   mean_overall_fc
##             <dbl>
## 1           0.263
```

```
#  si_control/si_CBX2)
  #group_by(day, siRNA) %>%

#floating
fold.change.float <- cbx2.cons.m.log2.float.expt5_7 %>% 
  group_by(day, siRNA) %>% 
  select(day, siRNA, cell_counts) %>% 
  summarize(mean_cell_count = mean(cell_counts)) %>%
  spread(siRNA, mean_cell_count) %>%
  mutate(fold_change_ctrl_cbx2 = (si_control -si_CBX2) /si_CBX2)
fold.change.float
```

```
## # A tibble: 5 x 4
## # Groups:   day [5]
##   day   si_control si_CBX2 fold_change_ctrl_cbx2
##   <ord>      <dbl>   <dbl>                 <dbl>
## 1 3           5167    5667               -0.0882
## 2 4          12167   14167               -0.141 
## 3 5          17667   15333                0.152 
## 4 6          23167   16167                0.433 
## 5 7          30650   13897                1.21
```

```
fold.change.float.overall <- fold.change.float %>% ungroup() %>% 
  summarise(mean_overall_fc = mean(fold_change_ctrl_cbx2))
fold.change.float.overall
```

```
## # A tibble: 1 x 1
##   mean_overall_fc
##             <dbl>
## 1           0.312
```

2018-10: Make changes suggested by reviewers

## Overlap between OCs and existing BrCa predictive tools

is there overlap between the genes we identified and mammaprint or oncomix genes?

```
mammaprint <- c("AA555029_RC", "ALDH4A1", "AP2B1", "AYTL2", "BBC3", "C16orf61", "C20orf46", "C9orf30", "CCNE2", "CDC42BPA", "CDCA7", "CENPA", "COL4A2", "DCK", "DIAPH3", "DTL", "EBF4", "ECT2", "EGLN1", "ESM1", "EXT1", "FGF18", "FLT1", "GMPS", "GNAZ", "GPR126", "GPR180", "GSTM3", "HRASLS", "IGFBP5", "JHDM1D", "KNTC2", "LGP2", "LIN9", "LOC100131053", "LOC100288906", "LOC730018", "MCM6", "MELK", "MMP9", "MS4A7", "MTDH", "NMU", "NUSAP1", "ORC6L", "OXCT1", "PALM2", "PECI", "PITRM1", "PRC1", "QSCN6L1", "RAB6B", "RASSF7", "RECQL5", "RFC4", "RTN4RL1", "RUNDC1", "SCUBE2", "SERF1A", "SLC2A3", "STK32B", "TGFB3", "TSPYL5", "UCHL5", "WISP1", "ZNF533")

oncotypedx <- c("MKI67", "AURKA", "BIRC5", "CCNB1", "MYBL2", "ERBB2", "GRB7", "ESR1", "PGR", "BCL2", "SCUBE2", "MMP11", "CTSL2", "GSTM1", "CD68", "BAG1", "ACTB", "GAPDH", "RPLPO", "GUS", "TFRC")

aims <- c("ABAT","ACADSB","ACTG2","ANXA3","APH1B","AR","ASH2L","ASPM","ATP2C2","BCL11A","BCL2","BIRC5","C1orf106","C5orf30","C6orf211","CA12","CAV1","CCNB2","CDC20","CDCA8","CDH3","CDKN1C","CDKN3","CDO1","CELSR1","CENPA","CENPF","CEP55","CIRBP","CKS2","CNIH4","CNN1","COL17A1","CRYAB","CSRP2","CSTB","CX3CL1","DNAJC12","DNALI1","DTL","ECSIT","EPHX2","EPN3","ERBB2","ESR1","FA2H","FAM134B","FAM49B","FBP1","FGFR4","FLNB","FMO5","FOXA1","FOXC1","GABRP","GAMT","GARS","GATA3","GFRA1","GRB7","GSN","GSTP1","HOXA5","HPN","HSPA14","ID4","IGBP1","IGF1","IGFBP6","IRS1","ITM2A","KIAA0020","KIAA0196","KIF13B","KIF2C","KIT","KRT14","KRT17","KRT18","KRT5","KYNU","LAMA3","LAPTM4B","LBR","LDHB","LumA","LumB","LYN","MAD2L1","MAP2K4","MAPT","MCM2","MELK","MEOX1","MFAP4","MKL2","MLF1IP","MLPH","MMP7","MNAT1","MYO10","NAT1","NDC80","NDRG2","NEK2","NEK4","NFIB","NormL","NPY1R","NQO1","PARP1","PBK","PCNA","PPAP2B","PRC1","PRKX","PSMD12","PTN","PTTG1","RACGAP1","RARRES1","RBBP8","RFC4","RRM2","S100A8","SAE1","SCUBE2","SDC1","SERPINA3","SFRP1","SHC2","SLC39A6","SOBP","SPDEF","STARD3","STC2","SYTL2","TCEAL1","TFCP2L1","TFF3","TIMELESS","TK1","TNFRSF21","TOP2A","TRIM29","TSHZ2","TSPAN1","TSPAN7","TTC12","TTK","TYMS","UBE2C","UBE2E3","VWFABAT","VWF")

aims_genes <- aims_subtypes$EntrezID.used

#map to gene symbols
ensembl = useMart("ensembl",dataset="hsapiens_gene_ensembl")
attributes = listAttributes(ensembl)
bm_aims <- getBM(attributes=c('external_gene_name', 'entrezgene'), 
      filters = 'entrezgene', 
      values = aims_genes, 
      mart = ensembl)


intersect(geneIdsTop5, mammaprint)
```

```
## character(0)
```

```
intersect(geneIdsTop5, oncotypedx)
```

```
## character(0)
```

```
intersect(geneIdsTop5, bm_aims$external_gene_name)
```

```
## character(0)
```

```
#aims_subtypes$EntrezID.used
```

## CBX2 association with survival within molecular subtypes

Is the CBX2 association with clinical outcome significant within the individual intrinsic subtypes? How do the patients with luminal A and B cancers with CBX2 over expression fare? Do they have a worse clinical outcome to other luminal A/B cancers with normal CBX2 expression?

To address this question, we performed kaplan meier survival analysis for each of the 5 known subtypes with at least 3 tumor samples per group. We did this for both the original 110 as well as for the entire dataset.

We start by preparing the entire dataset

```
rowData(e) = dplyr::left_join(data.frame(rowData(e)), ensg_to_prot_filt, by = c("ensembl_gene_id" = "Gene.stable.ID"))
aims_subtypes_all <- applyAIMS(assay(e),rowData(e)$NCBI.gene.ID)
```

```
## Current k = 20
```

```
e_cbx2_all$aims_subtypes <- aims_subtypes_all$cl[,1][colnames(e_cbx2_all)] #add aims_subtypes to this
#colData(e)$aims_subtypes <- aims_subtypes_all$cl[,1]

#e_cbx2_all replaces et_all
```

- first try it with the `et` dataset (110 samples)

```
et$years_last_fu_5yr <- et$days_last_fu_5yr/365.25 

brca_subtypes <- unique(et$aims_subtypes)
subtype_signif <- rep(NA, length(brca_subtypes))

for(st in 1:length(brca_subtypes)){
  subt <- brca_subtypes[st]
  et.st <- et[,et$aims_subtypes == subt]
  if(table(et.st$cl_ENSG00000173894) %>% length() == 1){
    subtype_signif[st] <- NA
    next
  }
  fit <- survfit(Surv(et.st$years_last_fu_5yr, as.numeric(et.st$vital_stat_5yr)) ~ as.factor(et.st$cl_ENSG00000173894), data = colData(et.st))
  fit.stat <- survival::survdiff(Surv(et.st$years_last_fu_5yr, as.numeric(et.st$vital_stat_5yr)) ~ as.factor(et.st$cl_ENSG00000173894))
  pval2 <- pvalFxn(fit.stat)
  subtype_signif[st] <- pval2
}
names(subtype_signif) <- brca_subtypes

subtype_signif
```

```
##     Basal      LumA      LumB      Her2    Normal 
## 0.9837661 0.3215067 0.1020868 0.5228596        NA
```

```
##     Basal      LumA      LumB      Her2    Normal 
## 0.9837661 0.3215067 0.1020868 0.5228596        NA

p.adjust(subtype_signif, method="BH")
```

```
##     Basal      LumA      LumB      Her2    Normal 
## 0.9837661 0.6430135 0.4083473 0.6971461        NA
```

```
##     Basal      LumA      LumB      Her2    Normal 
## 0.9837661 0.6430135 0.4083473 0.6971461        NA
```

- now, try it with the `e_cbx2_all` dataset (1084 samples with survival data)

```
### Nov 3, 2018
brca_subtypes_all <- unique(e_cbx2_all$aims_subtypes)
subtype_signif_all <- rep(NA, length(brca_subtypes))
for(st in 1:length(brca_subtypes_all)){
  subt <- brca_subtypes[st]
  et.st <- e_cbx2_all[,e_cbx2_all$aims_subtypes == subt]
  if(table(et.st$cbx2Class) %>% length() == 1){
    subtype_signif[st] <- NA
    next
  }
  fit <- survfit(Surv(et.st$years_last_fu_5yr, as.numeric(et.st$vital_stat_5yr)) ~ as.factor(et.st$cbx2Class), data = colData(et.st))
  fit.stat <- survival::survdiff(Surv(et.st$years_last_fu_5yr, as.numeric(et.st$vital_stat_5yr)) ~ as.factor(et.st$cbx2Class))
  pval2 <- pvalFxn(fit.stat)
  subtype_signif_all[st] <- pval2
  print(subt)
  print(fit.stat)
}
```

```
## [1] "Basal"
## Call:
## survival::survdiff(formula = Surv(et.st$years_last_fu_5yr, as.numeric(et.st$vital_stat_5yr)) ~ 
##     as.factor(et.st$cbx2Class))
## 
##                                N Observed Expected (O-E)^2/E (O-E)^2/V
## as.factor(et.st$cbx2Class)=0  25        3     2.71   0.03009     0.034
## as.factor(et.st$cbx2Class)=1 174       21    21.29   0.00384     0.034
## 
##  Chisq= 0  on 1 degrees of freedom, p= 0.854 
## [1] "LumA"
## Call:
## survival::survdiff(formula = Surv(et.st$years_last_fu_5yr, as.numeric(et.st$vital_stat_5yr)) ~ 
##     as.factor(et.st$cbx2Class))
## 
##                                N Observed Expected (O-E)^2/E (O-E)^2/V
## as.factor(et.st$cbx2Class)=0 305       17    15.82    0.0879     0.727
## as.factor(et.st$cbx2Class)=1  38        1     2.18    0.6382     0.727
## 
##  Chisq= 0.7  on 1 degrees of freedom, p= 0.394 
## [1] "LumB"
## Call:
## survival::survdiff(formula = Surv(et.st$years_last_fu_5yr, as.numeric(et.st$vital_stat_5yr)) ~ 
##     as.factor(et.st$cbx2Class))
## 
##                                N Observed Expected (O-E)^2/E (O-E)^2/V
## as.factor(et.st$cbx2Class)=0 194       12    16.98      1.46      4.21
## as.factor(et.st$cbx2Class)=1  98       14     9.02      2.75      4.21
## 
##  Chisq= 4.2  on 1 degrees of freedom, p= 0.0402 
## [1] "Her2"
## Call:
## survival::survdiff(formula = Surv(et.st$years_last_fu_5yr, as.numeric(et.st$vital_stat_5yr)) ~ 
##     as.factor(et.st$cbx2Class))
## 
##                                N Observed Expected (O-E)^2/E (O-E)^2/V
## as.factor(et.st$cbx2Class)=0  50       10     7.86     0.582     0.823
## as.factor(et.st$cbx2Class)=1 133       17    19.14     0.239     0.823
## 
##  Chisq= 0.8  on 1 degrees of freedom, p= 0.364 
## [1] "Normal"
## Call:
## survival::survdiff(formula = Surv(et.st$years_last_fu_5yr, as.numeric(et.st$vital_stat_5yr)) ~ 
##     as.factor(et.st$cbx2Class))
## 
##                               N Observed Expected (O-E)^2/E (O-E)^2/V
## as.factor(et.st$cbx2Class)=0 61        6    5.897   0.00181     0.108
## as.factor(et.st$cbx2Class)=1  6        0    0.103   0.10345     0.108
## 
##  Chisq= 0.1  on 1 degrees of freedom, p= 0.743
```

```
names(subtype_signif_all) <- brca_subtypes_all

subtype_signif_all
```

```
##       LumA       Her2      Basal       LumB     Normal 
## 0.85368790 0.39387430 0.04019842 0.36434383 0.74288516
```

```
##       LumA       Her2      Basal       LumB     Normal 
## 0.42784046 0.36434383 0.85368790 0.03889948 0.74288516 

p.adjust(subtype_signif_all, method="BH")
```

```
##      LumA      Her2     Basal      LumB    Normal 
## 0.8536879 0.6564572 0.2009921 0.6564572 0.8536879
```

```
##      LumA      Her2     Basal      LumB    Normal 
## 0.7130674 0.7130674 0.8536879 0.1944974 0.8536879
```

## Expression of MYC, CCND1, CCNE1, FGFR1/2 in adj nml tissue

```
mmp_tbl <- mmp %>% rownames_to_column("ensgID") %>% as_tibble() #filter()
ensg_oexpr <- c("ENSG00000136997" = "MYC", "ENSG00000110092" = "CCND1", "ENSG00000077782" = "FGFR1", "ENSG00000066468" = "FGFR2") #"ENSG00000105173" = "CCNE1"
mmp_tbl %>% filter(ensgID %in% names(ensg_oexpr))
```

```
## # A tibble: 4 x 13
##   ensgID         nMu1  nMu2  nVar   nPi1  tMu1  tMu2  tVar   tPi1 deltaMu2
##   <chr>         <dbl> <dbl> <dbl>  <dbl> <dbl> <dbl> <dbl>  <dbl>    <dbl>
## 1 ENSG00000110…  7.48  7.77 0.589 0.377   5.02  8.35  1.06 0.0433    0.577
## 2 ENSG00000077…  6.37  6.48 0.180 0.402   4.88  5.31  1.13 0.561    -1.17 
## 3 ENSG00000136…  6.08  8.21 0.466 0.0578  6.13  7.03  1.16 0.399    -1.18 
## 4 ENSG00000066…  2.58  5.33 0.552 0.118   3.99  4.73  1.62 0.499    -0.598
## # ... with 3 more variables: deltaMu1 <dbl>, SI <dbl>, score <dbl>
```

```
mmp_tbl %>% filter(ensgID %in% names(ensg_oexpr)) %>% select(-ensgID) %>% colMeans
```

```
##        nMu1        nMu2        nVar        nPi1        tMu1        tMu2 
##  5.62781582  6.94848218  0.44669542  0.23875939  5.00285769  6.35768709 
##        tVar        tPi1    deltaMu2    deltaMu1          SI       score 
##  1.24372793  0.37562482 -0.59079509 -0.62495813  0.08636364 -0.24993485
```

```
##        nMu1        nMu2        nVar        nPi1        tMu1        tMu2 
##  4.15867522  6.08978928  0.33359595  0.31023367  4.52041263  6.01810348 
##        tVar        tPi1    deltaMu2    deltaMu1          SI       score 
##  1.15981495  0.46534080 -0.07168581  0.36173741  0.27142857 -0.23342206 


mmp2 %>% dplyr::filter(geneNames %in% geneIdsTop5) %>% select(-geneNames) %>% colMeans
```

```
##      nMu1      nMu2      nVar      nPi1      tMu1      tMu2      tVar 
## 1.1387505 1.3839667 0.1979722 0.6066698 1.6940005 4.5215614 0.8482769 
##      tPi1  deltaMu2  deltaMu1        SI     score 
## 0.6265991 3.1375947 0.5552500 0.9963636 1.5307278
```

```
##       nMu1      nMu2      nVar      nPi1      tMu1      tMu2      
##  0.9038492 1.1136966 0.1742516 0.5999572 1.4754776 4.1858719  
##       tVar      tPi1  deltaMu2  deltaMu1        SI     score 
##  0.7720942 0.6224035 3.0721753 0.5716283 0.9964286 1.5488232 

#calculate the mean of the normal expression values from each group

en_tbl <- assay(en) %>% data.frame %>% rownames_to_column("ensg") %>% as_tibble()

en_tbl %>% filter(ensg %in% names(geneIdsTop5)) %>% select(-ensg) %>% rowMeans() %>% mean() #expression of oncomix genes in adj nml tissue
```

```
## [1] 1.227439
```

```
en_tbl %>% filter(ensg %in% names(ensg_oexpr)) %>% select(-ensg) %>% rowMeans() %>% mean() # expression of genes mentioned by reviewers in adj nml tissue
```

```
## [1] 6.797504
```

## Supplementary Figues 11-14: Oncomix applied to lung, endometrial, & prostate cancer datasets

- Oncomix can identify oncogene candidates in multiple cancer datasets containing variable numbers of samples

Download and filter data from lung cancer

```
## eq <- GDCquery(project = "TCGA-LUAD",
##                workflow.type = "HTSeq - FPKM",
##                data.category = c("Transcriptome Profiling"),
##                data.type = c("Gene Expression Quantification"))
## GDCdownload(eq)
## e <- GDCprepare(eq, save=FALSE)
## saveRDS(e, file=here("processed_data", "tcga-exp-luad.rds"))

e_luad <- readRDS(here("processed_data", "tcga-exp-luad.rds"))
```

Download and filter data from endometrial cancer

```
## eq <- GDCquery(project = "TCGA-UCEC",
##                workflow.type = "HTSeq - FPKM",
##                data.category = c("Transcriptome Profiling"),
##                data.type = c("Gene Expression Quantification"))
## GDCdownload(eq)
## e <- GDCprepare(eq, save=FALSE)
## saveRDS(e, file=here("processed_data", "tcga-exp-ucec-2.rds"))

e_ucec <- readRDS(here("processed_data", "tcga-exp-ucec-2.rds")) #most recent version, downloaded Nov 3, 2018
```

Download and filter data from prostate cancer

```
## eq <- GDCquery(project = "TCGA-PRAD",
##                workflow.type = "HTSeq - FPKM",
##                data.category = c("Transcriptome Profiling"),
##                data.type = c("Gene Expression Quantification"))
## GDCdownload(eq)
## e <- GDCprepare(eq, save=FALSE)
## saveRDS(e, file=here("processed_data", "tcga-exp-prad.rds"))

e_prad <- readRDS(here("processed_data", "tcga-exp-prad.rds")) #most recent version, downloaded Nov 3, 2018
```

Process for cleaning dataset

```
#remove FFPE samples
e_luad_temp <- clean_dataset(e_luad, ensg_to_prot)
```

```
## [1] 19090   513
## [1] 19090    59
## [1] 16336   512
## [1] 16333    59
```

```
e_ucec_temp <- clean_dataset(e_ucec, ensg_to_prot)
```

```
## [1] 19090   543
## [1] 19090    35
## [1] 15883   543
## [1] 16208    35
```

```
e_prad_temp <- clean_dataset(e_prad, ensg_to_prot)
```

```
## [1] 19090   495
## [1] 19090    52
## [1] 16407   495
## [1] 16599    52
```

```
e_luad_n <- e_luad_temp$en
e_luad_t <- e_luad_temp$et
e_ucec_n <- e_ucec_temp$en
e_ucec_t <- e_ucec_temp$et
e_prad_n <- e_prad_temp$en
e_prad_t <- e_prad_temp$et

rm(e_luad_temp, e_ucec_temp, e_prad_temp)
```

Now, apply oncomix to these datasets

```
if(file.exists(here("processed_data", "mmp_luad.rds"))){
  mmp_luad <- readRDS(file = here("processed_data", "mmp_luad.rds"))
} else {
  mmp_luad <- mixModelParams(exprNml = assay(e_luad_n), exprTum = assay(e_luad_t))
  #saveRDS(mmp_luad, file = here("processed_data", "mmp_luad.rds"))
}

if(file.exists(here("processed_data", "mmp_ucec.rds"))){
  mmp_ucec <- readRDS(file = here("processed_data", "mmp_ucec.rds"))
} else {
  mmp_ucec <- mixModelParams(exprNml = assay(e_ucec_n), exprTum = assay(e_ucec_t))
  #saveRDS(mmp_ucec, file = here("processed_data", "mmp_ucec.rds"))
}

if(file.exists(here("processed_data", "mmp_prad.rds"))){
  mmp_prad <- readRDS(file = here("processed_data", "mmp_prad.rds"))
} else {
  mmp_prad <- mixModelParams(exprNml = assay(e_prad_n), exprTum = assay(e_prad_t))
  #saveRDS(mmp_prad, file = here("processed_data", "mmp_prad.rds"))
}

#can also try this with the same genes as we found in the breast cancer dataset
```

make plots (top5 from each cancer type, also show CBX2 distribution). Make a table of the characteristics of these genes. Conclusion is that we can identify viable oncogene candidates in other cancer types using oncomix.

```
mmp_luad_filt <- filt_mmp_pi(mmp_luad, pi_min=0.2)
mmp_ucec_filt <- filt_mmp_pi(mmp_ucec, pi_min=0.2)
mmp_prad_filt <- filt_mmp_pi(mmp_prad, pi_min=0.2)


luad_top5 <- filt_genes_pi_si(mmp_luad, pi_min=0.2, si=0.99) %>% head(5)
ucec_top5 <- filt_genes_pi_si(mmp_ucec, pi_min=0.2, si=0.99) %>% head(5)
prad_top5 <- filt_genes_pi_si(mmp_prad, pi_min=0.2, si=0.99) %>% head(5)

#top5genes #breast cancer  #geneIdsTop5
```

7. Next, we’re going to plot the distributions of the top 5 selected isoforms with an SI > `selIdx_thresh`. We can also show the genes both before and after the selectivity index (ie low SI vs high SI).

```
gglayers = list(
    theme(axis.text.x = element_text(size=30),
          plot.title=element_text(size=30)),
  xlim(-0.2,10), xlab(""), ylab("")
)

dict_top5_mmp <- list(luad = list(luad_top5, mmp_luad, mmp_luad_filt, e_luad_n, e_luad_t), ucec = list(ucec_top5, mmp_ucec, mmp_ucec_filt, e_ucec_n, e_ucec_t), prad = list(prad_top5, mmp_prad, mmp_prad_filt, e_prad_n, e_prad_t), brca = list(top5genes, mmp, mmp2, en, et))

for(item in 1:length(dict_top5_mmp)){
  dict_item <- dict_top5_mmp[[item]]
  top5genes_temp <- dict_item[[1]]
  mmp_temp <- dict_item[[2]]
  mmp_filt_temp <- dict_item[[3]]
  tumor_type <- names(dict_top5_mmp)[item]
  exp_nml <- dict_item[[4]]
  exp_tum <- dict_item[[5]]

  #show the distribution of oncomix scores across genes
  plotOncomixScores(mmp_dat=mmp_filt_temp, si=0.99, alph=0.8)

  ggsave(filename = here("output", paste0(Sys.Date(),"_", tumor_type,"_oncomix_scores.png")),
           width = 7, height=5, units = "in")
  
  #plot CBX2 for each gene
  gglayers[["title"]] <- ggtitle("CBX2")
  p1 <- plotGeneHistUD(mmp_temp, assay(exp_nml), assay(exp_tum), "ENSG00000173894", linesz=1.5, gglayers)
  ggsave(filename = here("output", paste0(Sys.Date(),"_", tumor_type,"_cbx2.png")),
           plot = p1, width = 7, height=5, units = "in")
 

  for(i in 1:length(top5genes_temp)){
    hgnc_gene_symb <- ensg_to_prot %>% 
        dplyr::filter(Gene.stable.ID == top5genes_temp[i]) %>% 
        dplyr::select(HGNC.symbol) %>% 
        as.character()
    gglayers[["title"]] <- ggtitle(hgnc_gene_symb)
    plotGeneHistUD(mmp_temp, assay(exp_nml), assay(exp_tum), top5genes_temp[i], linesz=1.5, gglayers)
    ggsave(filename = here("output", paste0(Sys.Date(),"_",top5genes_temp[i], "_",tumor_type,"_histog.png")),
           width = 7, height=5, units = "in")
  }
}
```

```
#mmp_luad[luad_top5,]
hgnc_gene_symb_luad <- ensg_to_prot %>% 
    dplyr::filter(Gene.stable.ID %in% luad_top5)
hgnc_gene_symb_ucec <- ensg_to_prot %>% 
    dplyr::filter(Gene.stable.ID %in% ucec_top5)
hgnc_gene_symb_prad <- ensg_to_prot %>% 
    dplyr::filter(Gene.stable.ID %in% prad_top5)

mmp_luad_filt[luad_top5,]
```

```
##                      nMu1      nMu2       nVar      nPi1      tMu1
## ENSG00000187134 3.6581369 3.9090573 0.23802598 0.5604862 2.5369871
## ENSG00000104760 0.3413745 0.3993426 0.21713352 0.5239677 1.4728785
## ENSG00000143512 0.1979368 0.2325604 0.03464972 0.5659663 0.9522066
## ENSG00000074211 0.1662333 0.1949561 0.06807578 0.5279768 0.5582768
## ENSG00000203685 0.6825409 1.2098345 0.04345076 0.6649862 0.5615161
##                     tMu2      tVar      tPi1 deltaMu2   deltaMu1 SI
## ENSG00000187134 8.290974 1.9164708 0.7568101 4.381917 -1.1211497  1
## ENSG00000104760 6.931749 2.1473224 0.7424024 6.532406  1.1315039  1
## ENSG00000143512 4.804650 0.8475958 0.7194521 4.572089  0.7542697  1
## ENSG00000074211 3.842905 0.5562381 0.7190085 3.647949  0.3920435  1
## ENSG00000203685 4.048130 0.4560578 0.7993966 2.838296 -0.1210248  1
##                    score
## ENSG00000187134 3.348570
## ENSG00000104760 3.036447
## ENSG00000143512 2.935574
## ENSG00000074211 2.631592
## ENSG00000203685 2.459812
```

```
mmp_ucec_filt[ucec_top5,]
```

```
##                      nMu1     nMu2        nVar      nPi1      tMu1
## ENSG00000144407 0.3349394 1.302873 0.080686771 0.7143399 0.3596356
## ENSG00000105464 0.5494817 1.219765 0.024937659 0.7977214 1.6878711
## ENSG00000171956 0.2262360 1.472682 0.048900742 0.7739908 0.4142403
## ENSG00000159263 0.8911819 1.294689 0.026862794 0.4367342 1.1912037
## ENSG00000124140 0.3211505 0.617614 0.009385667 0.7367469 0.6706597
##                     tMu2      tVar      tPi1 deltaMu2   deltaMu1 SI
## ENSG00000144407 3.945496 0.3797388 0.7581404 2.642623 0.02469619  1
## ENSG00000105464 4.819607 0.7371933 0.4965516 3.599842 1.13838940  1
## ENSG00000171956 3.894673 0.5092749 0.7589731 2.421991 0.18800426  1
## ENSG00000159263 3.606586 0.5671638 0.4983658 2.311897 0.30002186  1
## ENSG00000124140 2.316188 0.2271221 0.7722145 1.698574 0.34950917  1
##                    score
## ENSG00000144407 2.157501
## ENSG00000105464 1.699321
## ENSG00000171956 1.675811
## ENSG00000159263 1.417848
## ENSG00000124140 1.112557
```

```
mmp_prad_filt[prad_top5,]
```

```
##                      nMu1     nMu2       nVar      nPi1      tMu1     tMu2
## ENSG00000103111 5.0004520 5.947607 0.13591512 0.5119697 5.7448699 8.314026
## ENSG00000127588 0.3401660 1.583877 0.09411245 0.7751049 1.2229182 3.648904
## ENSG00000197558 0.3785834 1.058948 0.03467521 0.7919055 0.9103318 2.115098
## ENSG00000141696 4.5620935 4.940011 0.05277128 0.3735454 5.1985208 6.011682
## ENSG00000142544 2.1021616 2.387614 0.15905386 0.4006381 2.9541089 3.729702
##                      tVar      tPi1 deltaMu2  deltaMu1 SI     score
## ENSG00000103111 0.6586718 0.7671649 2.366419 0.7444179  1 0.8274142
## ENSG00000127588 0.5589943 0.7685654 2.065027 0.8827522  1 0.5291679
## ENSG00000197558 0.1851788 0.7901408 1.056150 0.5317484  1 0.3045476
## ENSG00000141696 0.2186459 0.7998280 1.071671 0.6364273  1 0.1638264
## ENSG00000142544 0.1719460 0.6542453 1.342088 0.8519473  1 0.1591408
```

```
# How many genes in each group? (SI > and < 0.99)
dim(subset(mmp_luad_filt, SI<.99))
```

```
## [1] 3868   12
```

```
dim(subset(mmp_luad_filt, SI>.99))
```

```
## [1] 581  12
```

```
dim(subset(mmp_prad_filt, SI<.99))
```

```
## [1] 3241   12
```

```
dim(subset(mmp_prad_filt, SI>.99))
```

```
## [1] 20 12
```

```
dim(subset(mmp_ucec_filt, SI<.99))
```

```
## [1] 4233   12
```

```
dim(subset(mmp_ucec_filt, SI>.99))
```

```
## [1] 224  12
```

```
dim(subset(mmp2, SI<.99))
```

```
## [1] 3669   13
```

```
dim(subset(mmp2, SI>.99))
```

```
## [1] 154  13
```

What’s the CBX2 rank across the 3 cancer types?

```
#mmp_luad %>% rownames_to_column("ensg") %>% mutate(rank=1:n()) %>% filter(ensg == "ENSG00000173894") 
mmp_luad_filt %>% rownames_to_column("ensg") %>% filter(SI > 0.99) %>% mutate(rank=1:n()) %>% filter(ensg == "ENSG00000173894")
```

```
##              ensg     nMu1     nMu2      nVar      nPi1     tMu1     tMu2
## 1 ENSG00000173894 1.255722 2.235828 0.1322124 0.6746648 2.558439 4.754407
##        tVar      tPi1 deltaMu2 deltaMu1 SI     score rank
## 1 0.8431715 0.7306803 2.518579 1.302717  1 0.2404778  189
```

```
mmp_luad_filt %>% rownames_to_column("ensg") %>% filter(SI > 0.99) %>% mutate(rank=1:n()) %>% dim() #nrows
```

```
## [1] 581  14
```

```
#mmp_ucec %>% rownames_to_column("ensg") %>% mutate(rank=1:n()) %>% filter(ensg == "ENSG00000173894") 
mmp_ucec_filt %>% rownames_to_column("ensg") %>% filter(SI > 0.99) %>% mutate(rank=1:n()) %>% filter(ensg == "ENSG00000173894") #189
```

```
##              ensg     nMu1     nMu2      nVar     nPi1     tMu1     tMu2
## 1 ENSG00000173894 2.152284 2.966645 0.1418946 0.643624 2.673769 4.888474
##        tVar      tPi1 deltaMu2  deltaMu1 SI     score rank
## 1 0.7687237 0.2148887 1.921829 0.5214852  1 0.4897253   28
```

```
mmp_ucec_filt %>% rownames_to_column("ensg") %>% filter(SI > 0.99) %>% mutate(rank=1:n()) %>% dim() #3868
```

```
## [1] 224  14
```

```
#mmp_prad %>% rownames_to_column("ensg") %>% mutate(rank=1:n()) %>% filter(ensg == "ENSG00000173894") 
mmp_prad_filt %>% rownames_to_column("ensg") %>% filter(SI > 0.99) %>% mutate(rank=1:n()) %>% filter(ensg == "ENSG00000173894")
```

```
##  [1] ensg     nMu1     nMu2     nVar     nPi1     tMu1     tMu2    
##  [8] tVar     tPi1     deltaMu2 deltaMu1 SI       score    rank    
## <0 rows> (or 0-length row.names)
```

```
mmp_prad_filt %>% rownames_to_column("ensg") %>% filter(SI > 0.99) %>% mutate(rank=1:n()) %>% dim() #20
```

```
## [1] 20 14
```

```
#mmp %>% rownames_to_column("ensg") %>% mutate(rank=1:n()) %>% filter(ensg == "ENSG00000173894") #404 / 15638; 
mmp2 %>% rownames_to_column("ensg") %>% filter(SI > 0.99) %>% mutate(rank=1:n()) %>% filter(ensg == "CBX2") #3 / 139
```

```
##   ensg     nMu1     nMu2     nVar      nPi1     tMu1    tMu2      tVar
## 1 CBX2 1.882826 2.346459 0.165679 0.6763193 2.387308 5.05406 0.5561141
##        tPi1 deltaMu2  deltaMu1 SI    score geneNames rank
## 1 0.5785395 2.707601 0.5044819  1 1.481326      CBX2    3
```

```
mmp2 %>% rownames_to_column("ensg") %>% filter(SI > 0.99) %>% mutate(rank=1:n()) %>% dim() #3 / 139
```

```
## [1] 154  15
```

## Supplementary Figure 2 - Power analysis

```
mmp3 <- mmp %>% filter(SI > .99, tPi1 < 0.8, mmp$tPi1 > 0.2, 
                       mmp$nPi1 < 0.8, mmp$nPi1 > 0.2) #rownames_to_column()
top5_mmp <- head(mmp3, 5) %>% as_tibble()
not5_mmp <- mmp3[6:nrow(mmp3),] %>% as_tibble()
top5_mmp
```

```
## # A tibble: 5 x 12
##     nMu1  nMu2   nVar  nPi1  tMu1  tMu2  tVar  tPi1 deltaMu2 deltaMu1
## *  <dbl> <dbl>  <dbl> <dbl> <dbl> <dbl> <dbl> <dbl>    <dbl>    <dbl>
## 1 0.170  0.201 0.0755 0.546 1.39   4.43 1.09  0.647     4.23    1.22 
## 2 2.02   2.42  0.460  0.614 2.20   6.35 1.64  0.635     3.93    0.176
## 3 1.88   2.35  0.166  0.676 2.39   5.05 0.556 0.579     2.71    0.504
## 4 0.0944 0.125 0.0295 0.569 0.811  2.59 0.315 0.497     2.47    0.716
## 5 1.53   1.83  0.259  0.629 1.68   4.18 0.639 0.776     2.35    0.158
## # ... with 2 more variables: SI <dbl>, score <dbl>
```

```
not5_mmp
```

```
## # A tibble: 149 x 12
##     nMu1  nMu2   nVar  nPi1  tMu1  tMu2  tVar  tPi1 deltaMu2 deltaMu1
##  * <dbl> <dbl>  <dbl> <dbl> <dbl> <dbl> <dbl> <dbl>    <dbl>    <dbl>
##  1 0.387 0.470 0.148  0.600 0.657  2.47 0.297 0.752     2.00   0.270 
##  2 0.277 0.346 0.135  0.583 0.794  2.42 0.304 0.537     2.07   0.518 
##  3 1.25  2.07  0.291  0.392 1.60   4.75 0.954 0.752     2.67   0.343 
##  4 0.895 1.02  0.168  0.375 1.16   2.81 0.277 0.778     1.79   0.269 
##  5 0.797 1.23  0.0763 0.628 1.39   3.31 0.367 0.688     2.08   0.597 
##  6 1.04  1.14  0.116  0.547 1.33   3.10 0.606 0.750     1.96   0.291 
##  7 2.48  3.08  0.130  0.229 2.74   5.12 0.719 0.675     2.05   0.260 
##  8 0.834 0.959 0.234  0.603 0.851  2.45 0.334 0.752     1.49   0.0176
##  9 4.49  4.54  0.119  0.450 5.06   6.25 0.187 0.758     1.70   0.576 
## 10 0.946 1.52  0.0814 0.581 1.44   3.22 0.329 0.767     1.70   0.491 
## # ... with 139 more rows, and 2 more variables: SI <dbl>, score <dbl>
```

3. To calculate power, we first define a null and alternative hypothesis.

The **null hypothesis** is defined as there being no significant difference in the oncomix score of the top 5 ranked oncogene candidates (`top5_mmp`) relative to the rest of the 134 genes (`not5_mmp`) that passed the initial filters (0.2 > \(π\_T\) & \(π\_N\) > 0.8, selectivity index > 0.99).

The **alternative hypothesis** is that the oncomix scores of the top 5 ranked oncogene candidates are significantly higher than those genes not ranking in the top 5.

We first test the likelihood of the null hypothesis being true in our observed data:

```
obs_score_diff <- t.test(top5_mmp$score, not5_mmp$score, alternative = "greater")
obs_score_diff
```

```
## 
##  Welch Two Sample t-test
## 
## data:  top5_mmp$score and not5_mmp$score
## t = 15.479, df = 6.4548, p-value = 1.204e-06
## alternative hypothesis: true difference in means is greater than 0
## 95 percent confidence interval:
##  1.487059      Inf
## sample estimates:
##  mean of x  mean of y 
##  1.5307278 -0.1667759
```

We see that the oncomix scores from the top 5 genes are significantly greater than the oncomix scores of the bottom 134 genes (p = 1.210^{-6}).

Now, to calculate the power of rejecting the null hypothesis, we sample from the (joint) parameter spaces of these two groups that we know are different (`not5_mmp` and `top5_mmp`). We then recalculate the oncomix scores based on the sampled parameters, and repeat the same statistical test as above on 1000 simulated datasets. Our power is calculated as the % of times that we correctly reject the null hypothesis.

We perform the power calculation by sampling from a multivariate Gaussian (except SI, which is done using bootstrap due to narrow range (0-1) and non-Gaussianity).

```
n_sims <- 1000
sim_res_multiv <- sapply(1:n_sims, function(x) calc_rand_pval_effect_size_multiv(top5_mmp, not5_mmp))
perc_power_multiv <- 100*sum(sim_res_multiv["pval",] < 0.05)/n_sims
perc_power_multiv #at a p-value of 0.05
```

```
## [1] 100
```

```
perc_power_multiv_obs_p <- 100*sum(sim_res_multiv["pval",] < obs_score_diff$p.value)/n_sims 
perc_power_multiv_obs_p #at p-value of obs_score_diff$p.value
```

```
## [1] 72.1
```

Lastly, we show the power as a function of the p-value

```
#show power versus p-value
p_val_thresh <- c(seq(1e-6, 0.05, by = 0.000001), 0.05)
power <- sapply(p_val_thresh, function(x) sum(sim_res_multiv["pval",] < x)/n_sims )
df <- tibble(p_val_thresh, power)
ggplot(df, aes(x=p_val_thresh, y=power)) + 
  geom_point() + 
  scale_x_log10() + 
  geom_vline(xintercept=c(obs_score_diff$p.value, 0.05), 
             color=c("red", "blue")) + 
  xlab("p-value") + ylab("Power") + theme_bw() + 
  ggtitle("Power as a function of the p-value \n Red line is observed p-value threshold \n Blue line is p=0.05") + 
  theme(plot.title = element_text(hjust = 0.5)) + 
  scale_x_log10(breaks = trans_breaks("log10", function(x) 10^x),
                labels = trans_format("log10", math_format(10^.x))) + 
                annotation_logticks(sides="b")
```

```
## Scale for 'x' is already present. Adding another scale for 'x', which
## will replace the existing scale.
```

```
#red line indicates the observed p-value threshold in the study
#blue line indicates p=0.05 ()
```

## Summary of data sources:

Below, we document the sources and dates of retrieval when each data file was downloaded from:

1. TCGA mRNA expression data with clinical annotations were downloaded in November 2018, GDC Version 0.13.0, using `TCGAbiolinks` R package. Genes aligned using standard GDC pipelines and annotated to human genome GRCh38.p12. Additional clinical annotations (e.g. % tumor cells, histology, and retrospective collection) were downloaded using the `GenomicDataCommons` R package.
2. TCGA tumor CpG 450k methylation data was downloaded in June 2018, GDC Version 0.12.0, using `GenomicDataCommons` and `TCGAbiolinks` R packages. CpG sites were lifted over from hg19 to hg38 using the `liftover` R package.
3. GTEx data was downloaded from https://www.gtexportal.org/home/datasets as “GTEx\_Analysis\_2016-01-15\_v7\_RNASeQCv1.1.8\_gene\_median\_tpm.gct.gz” in March 2018 and then gunzipped using the following command:

gunzip GTEx\_Analysis\_2016-01-15\_v7\_RNASeQCv1.1.8\_gene\_median\_tpm.gct.gz

4. ChIP-seq data was downloaded from the ENCODE version 3 database in April 2017 using the `rutils` tool. ENCFF accession ID’s are listed below for the downloaded files:

ENCFF024TEH.bed; ENCFF180XXZ.bed; ENCFF329QYZ.bed; ENCFF541DRZ.bed; ENCFF694ZRC.bed; ENCFF921OMT.bed; ENCFF042AWM.bed; ENCFF184DUF.bed; ENCFF343QQE.bed; ENCFF541HRT.bed; ENCFF708ACK.bed; ENCFF928YTD.bed; ENCFF046BRP.bed; ENCFF190ZXX.bed; ENCFF401IAI.bed; ENCFF569ZCY.bed; ENCFF730UAD.bed; ENCFF932XEU.bed; ENCFF083AZM.bed; ENCFF209WRW.bed; ENCFF408TWV.bed; ENCFF577EMC.bed; ENCFF737BNW.bed; ENCFF942TCG.bed; ENCFF105PFS.bed; ENCFF220RUS.bed; ENCFF432GSK.bed; ENCFF578NMN.bed; ENCFF762CDY.bed; ENCFF947ABI.bed; ENCFF105VHD.bed; ENCFF225VFR.bed; ENCFF434DYI.bed; ENCFF583NFB.bed; ENCFF762MGC.bed; ENCFF993FPN.bed; ENCFF140SFK.bed; ENCFF233RBO.bed; ENCFF441UHA.bed; ENCFF618NVV.bed; ENCFF780WLS.bed; ENCFF150PTQ.bed; ENCFF247VVK.bed; ENCFF456MGR.bed; ENCFF621ZSK.bed; ENCFF784QFH.bed; ENCFF160RLI.bed; ENCFF269RME.bed; ENCFF464QAL.bed; ENCFF625IUE.bed; ENCFF800CDQ.bed; ENCFF161SFU.bed; ENCFF275WAD.bed; ENCFF495PCJ.bed; ENCFF666YGQ.bed; ENCFF838LXI.bed; ENCFF170POB.bed; ENCFF300OKR.bed; ENCFF496RVC.bed; ENCFF679UGD.bed; ENCFF899MQW.bed; ENCFF179TFZ.bed; ENCFF306PBX.bed; ENCFF514BKI.bed; ENCFF687REM.bed; ENCFF907UNK.bed

5. CNV data were downloaded in January 2018, GDC Version 0.10.0, using the `GenomicDataCommons` R package.
6. Mutation data were downloaded in January 2018, GDC Version 0.10.0, using the `GDCquery_Maf`function from the `TCGAbiolinks` R package.

## R package versions

```
sessionInfo()
```

```
## R version 3.4.3 (2017-11-30)
## Platform: x86_64-apple-darwin15.6.0 (64-bit)
## Running under: OS X El Capitan 10.11.6
## 
## Matrix products: default
## BLAS: /Library/Frameworks/R.framework/Versions/3.4/Resources/lib/libRblas.0.dylib
## LAPACK: /Library/Frameworks/R.framework/Versions/3.4/Resources/lib/libRlapack.dylib
## 
## locale:
## [1] en_US.UTF-8/en_US.UTF-8/en_US.UTF-8/C/en_US.UTF-8/en_US.UTF-8
## 
## attached base packages:
## [1] stats4    parallel  methods   stats     graphics  grDevices utils    
## [8] datasets  base     
## 
## other attached packages:
##  [1] scales_1.0.0                           
##  [2] bindrcpp_0.2.2                         
##  [3] janitor_0.4.0.9000                     
##  [4] forcats_0.2.0                          
##  [5] stringr_1.3.1                          
##  [6] dplyr_0.7.7                            
##  [7] purrr_0.2.5                            
##  [8] readr_1.1.1                            
##  [9] tidyr_0.8.1                            
## [10] tibble_1.4.2                           
## [11] tidyverse_1.2.1                        
## [12] readxl_1.0.0                           
## [13] ggdendro_0.1-20                        
## [14] RColorBrewer_1.1-2                     
## [15] ggrepel_0.8.0.9000                     
## [16] ggplot2_3.1.0                          
## [17] SummarizedExperiment_1.8.1             
## [18] DelayedArray_0.4.1                     
## [19] matrixStats_0.52.2                     
## [20] XML_3.98-1.16                          
## [21] here_0.1                               
## [22] biomaRt_2.34.2                         
## [23] GenomicDataCommons_1.2.0               
## [24] magrittr_1.5                           
## [25] rtracklayer_1.38.3                     
## [26] FDb.InfiniumMethylation.hg19_2.2.0     
## [27] org.Hs.eg.db_3.5.0                     
## [28] TxDb.Hsapiens.UCSC.hg19.knownGene_3.2.2
## [29] TxDb.Hsapiens.UCSC.hg38.knownGene_3.4.0
## [30] GenomicFeatures_1.30.3                 
## [31] AnnotationDbi_1.40.0                   
## [32] GenomicRanges_1.30.3                   
## [33] GenomeInfoDb_1.14.0                    
## [34] IRanges_2.12.0                         
## [35] S4Vectors_0.16.0                       
## [36] GSA_1.03                               
## [37] AIMS_1.10.0                            
## [38] Biobase_2.38.0                         
## [39] BiocGenerics_0.24.0                    
## [40] e1071_1.6-8                            
## [41] TCGAbiolinks_2.9.5                     
## [42] AUC_0.3.0                              
## [43] survival_2.41-3                        
## [44] limma_3.34.9                           
## [45] glmnet_2.0-13                          
## [46] foreach_1.4.4                          
## [47] Matrix_1.2-12                          
## [48] oncomix_0.99.3                         
## [49] mclust_5.4                             
## [50] rmarkdown_1.9.17                       
## 
## loaded via a namespace (and not attached):
##   [1] backports_1.1.2             circlize_0.4.4             
##   [3] aroma.light_3.8.0           plyr_1.8.4                 
##   [5] selectr_0.4-1               ConsensusClusterPlus_1.42.0
##   [7] lazyeval_0.2.1              splines_3.4.3              
##   [9] BiocParallel_1.12.0         sva_3.26.0                 
##  [11] digest_0.6.18               htmltools_0.3.6            
##  [13] memoise_1.1.0               cluster_2.0.6              
##  [15] doParallel_1.0.14           ComplexHeatmap_1.17.1      
##  [17] Biostrings_2.46.0           annotate_1.56.1            
##  [19] modelr_0.1.1                R.utils_2.7.0              
##  [21] prettyunits_1.0.2           colorspace_1.3-2           
##  [23] blob_1.1.1                  rvest_0.3.2                
##  [25] haven_1.1.1                 crayon_1.3.4               
##  [27] RCurl_1.95-4.11             jsonlite_1.5               
##  [29] genefilter_1.60.0           bindr_0.1.1                
##  [31] zoo_1.8-4                   iterators_1.0.10           
##  [33] glue_1.3.0                  survminer_0.4.3            
##  [35] gtable_0.2.0                zlibbioc_1.24.0            
##  [37] XVector_0.18.0              GetoptLong_0.1.6           
##  [39] shape_1.4.4                 abind_1.4-5                
##  [41] DESeq_1.30.0                GGally_1.3.2               
##  [43] DBI_1.0.0                   edgeR_3.20.9               
##  [45] ggthemes_4.0.1              Rcpp_0.12.19               
##  [47] xtable_1.8-3                progress_1.2.0             
##  [49] cmprsk_2.2-7                foreign_0.8-69             
##  [51] bit_1.1-12                  matlab_1.0.2               
##  [53] km.ci_0.5-2                 httr_1.3.1                 
##  [55] reshape_0.8.7               pkgconfig_2.0.2            
##  [57] R.methodsS3_1.7.1           utf8_1.1.3                 
##  [59] locfit_1.5-9.1              labeling_0.3               
##  [61] tidyselect_0.2.5            rlang_0.3.0.1              
##  [63] reshape2_1.4.3              cellranger_1.1.0           
##  [65] munsell_0.5.0               tools_3.4.3                
##  [67] cli_1.0.0                   downloader_0.4             
##  [69] RSQLite_2.1.1               broom_0.4.5                
##  [71] evaluate_0.10.1             yaml_2.1.19                
##  [73] knitr_1.20                  bit64_0.9-7                
##  [75] survMisc_0.5.5              EDASeq_2.12.0              
##  [77] nlme_3.1-131                R.oo_1.22.0                
##  [79] xml2_1.2.0                  rstudioapi_0.7             
##  [81] compiler_3.4.3              curl_3.1                   
##  [83] geneplotter_1.56.0          stringi_1.2.4              
##  [85] highr_0.6                   lattice_0.20-35            
##  [87] psych_1.8.4                 KMsurv_0.1-5               
##  [89] pillar_1.1.0                GlobalOptions_0.1.0        
##  [91] data.table_1.11.8           bitops_1.0-6               
##  [93] R6_2.3.0                    latticeExtra_0.6-28        
##  [95] hwriter_1.3.2               RMySQL_0.10.15             
##  [97] ShortRead_1.36.0            gridExtra_2.3              
##  [99] codetools_0.2-15            MASS_7.3-48                
## [101] assertthat_0.2.0            rprojroot_1.3-2            
## [103] rjson_0.2.15                withr_2.1.2                
## [105] GenomicAlignments_1.14.2    Rsamtools_1.30.0           
## [107] mnormt_1.5-5                GenomeInfoDbData_1.0.0     
## [109] mgcv_1.8-23                 hms_0.4.2                  
## [111] grid_3.4.3                  class_7.3-14               
## [113] snakecase_0.8.3.1           ggpubr_0.1.8               
## [115] lubridate_1.7.4
```

## Supplementary scripts

The functions found in `2017_12_suppl_scripts.R` are listed below

```
scatterMixPlot2 <- function (mmParams, selIndThresh = 1, geneLabels = NULL, sizesX, sizesY, ...) {
  mmParams <- as.data.frame(mmParams)
  oneOverAlpha <- diff(range(mmParams$deltaMu2))
  alpha1 <- 1/oneOverAlpha
  quants <- c(0.05, 0.5, 0.95)
  colorsRed <- RColorBrewer::brewer.pal(n = length(quants), 
                                        name = "Reds")
  deltaMu2Quant <- stats::quantile(mmParams[, "deltaMu2"], 
                                   quants)
  deltaMu1Quant <- stats::quantile(1/(abs(mmParams[, "deltaMu1"]) + 
                                        alpha1), quants)
  deltaMu2 <- deltaMu1 <- NULL
  x <- ggplot(data = as.data.frame(mmParams), aes(x = deltaMu2, 
                                                  y = 1/(abs(deltaMu1) + alpha1))) + theme_classic() + 
    geom_hline(yintercept = deltaMu1Quant, col = colorsRed, 
               size = sizesY) + geom_vline(xintercept = deltaMu2Quant, 
                                           col = colorsRed, size = sizesX) + geom_point(alpha = 0.5) + 
    xlab(expression(paste(Delta, mu[2]))) + ylab(expression(paste(frac(1, 
                                                                       paste(Delta, mu[1], " + ", alpha)))))# + ...
  if (selIndThresh < 1) {
    mmParamsSi <- mmParams[mmParams$SI > selIndThresh, ]
    x <- x + geom_point(data = as.data.frame(mmParamsSi), 
                        aes(x = deltaMu2, y = 1/(abs(deltaMu1) + alpha1)), 
                        size = 10, alpha = 0.1, col = colorsRed[length(colorsRed)], 
                        fill = colorsRed[length(colorsRed)]) + ggtitle(bquote(Distribution ~ 
                                                                                of ~ Mixture ~ Model ~ Parameters * "," ~ alpha ~ 
                                                                                "=" ~ .(round(alpha1, 2)) * ", SI >" ~ .(selIndThresh))) + ...
  }
  else if (!is.null(geneLabels)) {
    mmParamsSi <- mmParams[geneLabels, ]
    mmParamsSi$geneLabels <- geneLabels
    x <- x + geom_point(data = as.data.frame(mmParamsSi), 
                        aes(x = deltaMu2, y = 1/(abs(deltaMu1) + alpha1)), 
                        size = 10, alpha = 0.1, col = colorsRed[length(colorsRed)], 
                        fill = colorsRed[length(colorsRed)]) + ggrepel::geom_text_repel(data = mmParamsSi, 
                                                                                        aes(x = deltaMu2, y = 1/(abs(deltaMu1) + alpha1)), 
                                                                                        label = rownames(mmParamsSi)) + ggtitle(bquote(Distribution ~ 
                                                                                                                                         of ~ Mixture ~ Model ~ Parameters * "," ~ alpha ~ 
                                                                                                                                         "=" ~ .(round(alpha1, 2)))) + ...
  }
  else {
    x <- x + ggtitle(bquote(Distribution ~ of ~ Mixture ~ 
                              Model ~ Parameters * "," ~ alpha ~ "=" ~ .(round(alpha1, 
                                                                               2)))) + ...
  }
  return(x)
}


#part 9.

classifyTumors <- function(mmp, genes, exprMat){
  #get the midpoint between the 2 tumor curves
  mmp_diff <- apply(mmp[genes, c("tMu2", "tMu1")], 1, mean)
  boolMtrx = t(assay(exprMat)[genes, ] > mmp_diff)
  boolMtrx <- ifelse(boolMtrx, 1, 0)
  colnames(boolMtrx) <- paste0("cl_", colnames(boolMtrx))
  return(boolMtrx)
}


returnDeGenesLimma <- function(expr, design, colOfInt, pval=1, logFC=0, retAll=F){
  fit_t <- lmFit(object = expr, design = design)
  ebfit_t <- eBayes(fit_t)
  if(retAll == T){
    topTableRes_t <- topTable(ebfit_t, coef = colOfInt, number = Inf) 
    return(topTableRes_t)
  }
  topTableRes_t <- topTable(ebfit_t, coef = colOfInt, number = nrow(assay(expr)), adjust.method = "BH", p.value = pval, lfc = logFC) 
  return(topTableRes_t)
}

genesetEnrichmentFisher <- function(B, Targ, GS.GS, GS.names){ 
  #calculates the fisher exact t test for enrichment in a particular set of
  #target gene entrez IDs (T) among the number of background genes (B). GS.GS
  #takes the form "hGMT$genesets", where hGMT is a variable that stores the .gmt
  #file object downloaded from the msigdb website that was read in using the
  #GMT() function. GS.names takes the form "hGMT$geneset.names"
  
  #1. Initialize an empty dataframe
  t <- unlist(fisher.test(matrix(data = c(1,4,6,2), nrow = 2), conf.int = T))
  colNam <- c(names(t), "geneset.names", "MatchesBtwTargAndPathway", "NumbGenesInMsigdbPathway", "TargLen", "BackgroundLen")
  fisherDf <- data.frame(matrix(NA, nrow=1, ncol=13))
  names(fisherDf) <- colNam
  
  TargLen <- length(Targ)
  #2. Loop through each geneset and calculate pvalues
  for(i in 1:length(GS.GS)){
    pathwayEntrezIDs <- GS.GS[[i]] #these are characters
    M <- length(intersect(Targ, pathwayEntrezIDs)) #number of overlapping genes
    P <- length(pathwayEntrezIDs) #numb of genes in pathway
    conting.matrix <- matrix(c(M, TargLen-M, P-M, B-TargLen-P+M), nrow = 2, byrow = TRUE) #create contigency matrix
    fisherTest <- fisher.test(conting.matrix, conf.int = T)
    fisherDf <- rbind(fisherDf, c(unlist(fisherTest), GS.names[[i]], M, P, TargLen, B))
  }
  fisherDf = fisherDf[-1,]   #remove the row of NA's (in row 1)
  fisherDf$bhPval <- p.adjust(fisherDf$p.value, method = "BH")
  fisherDf$bonferroni <- p.adjust(fisherDf$p.value, method = "bonferroni")
  fisherDf$neg.log10.bhPval <- -log10(as.numeric(fisherDf$bhPval))
  fisherDf$dataset <-
    unlist(lapply(strsplit(fisherDf$geneset.names, "_"), function(x) x[1]))
  fisherDf <- fisherDf[order(fisherDf$bhPval),]
  #rank by bhPval pbal
  return(fisherDf)
}


gseWrapper <- function(B, Targ, genesets, geneset.names, qval, OR){
  #dir can equal "up" or "down" for up or down regulated, respectivtly
  
  #Targ = upregDeGenesEntrez; genesets=hallmarkKeggReactome$genesets;
  #geneset.names = hallmarkKeggReactome$geneset.names; qval=qval_pathway; OR=OR_pathway
  
  res_gse <- genesetEnrichmentFisher(B = B, Targ = Targ, GS.GS = genesets, GS.names=geneset.names) 
  res_gse_filt <- res_gse[as.numeric(res_gse$bhPval) < qval & 
                            as.numeric(res_gse$conf.int1) > OR & 
                            as.numeric(res_gse$conf.int1) > OR,] #`estimate.odds ratio`
  return(res_gse_filt) 
}


GSA.read.gmt.silent <- function (filename) {
  a = scan(filename, what = list("", ""), sep = "\t", quote = NULL, 
           fill = T, flush = T, multi.line = F)
  geneset.names = a[1][[1]]
  geneset.descriptions = a[2][[1]]
  dd = scan(filename, what = "", sep = "\t", quote = NULL)
  nn = length(geneset.names)
  n = length(dd)
  ox = rep(NA, nn)
  ii = 1
  for (i in 1:nn) {
    #cat(i)
    while ((dd[ii] != geneset.names[i]) | (dd[ii + 1] != 
                                           geneset.descriptions[i])) {
      ii = ii + 1
    }
    ox[i] = ii
    ii = ii + 1
  }
  genesets = vector("list", nn)
  for (i in 1:(nn - 1)) {
    #cat(i, fill = T)
    i1 = ox[i] + 2
    i2 = ox[i + 1] - 1
    geneset.descriptions[i] = dd[ox[i] + 1]
    genesets[[i]] = dd[i1:i2]
  }
  geneset.descriptions[nn] = dd[ox[nn] + 1]
  genesets[[nn]] = dd[(ox[nn] + 2):n]
  out = list(genesets = genesets, geneset.names = geneset.names, 
             geneset.descriptions = geneset.descriptions)
  class(out) = "GSA.genesets"
  return(out)
}


readHmKgRctmepathways <- function(gsea_path){
  hallmark <- GSA.read.gmt.silent(paste0(gsea_path,"/h.all.v5.1.entrez.gmt")) #50 genesets
  kegg <- GSA.read.gmt.silent(paste0(gsea_path,"/c2.cp.kegg.v5.1.entrez.gmt")) #185 genesets
  reactome <- GSA.read.gmt.silent(paste0(gsea_path,"/c2.cp.reactome.v5.1.entrez.gmt")) #673 genesets
  hallmarkKeggReactome <- list(genesets = c(hallmark$genesets, kegg$genesets, reactome$genesets), geneset.names =
                                 c(hallmark$geneset.names, kegg$geneset.names, reactome$geneset.names), geneset.descriptions =
                                 c(hallmark$geneset.descriptions, kegg$geneset.descriptions, reactome$geneset.descriptions))
  names(hallmarkKeggReactome[[1]]) <- hallmarkKeggReactome$geneset.names
  return(hallmarkKeggReactome)
}

forestplot <- function(d, title, xlab=expression(log[10] *"(Odds Ratio)"), ylab="Pathway"){
  p <- ggplot(d, aes(x=x, y=y, ymin=ylo, ymax=yhi)) +
    geom_pointrange(size = 1.25) +
    coord_flip() +
    #geom_hline(aes(x=0, yintercept = 1)) +
    ylab(xlab) +
    xlab(ylab) + #switch because of the coord_flip() above
    ggtitle(title) +
    scale_y_log10() +
    theme_classic() +
    geom_text(aes(label=numbMatches)) +
    guides(alpha=FALSE) + 
    theme(axis.text.x=element_text(size=12), axis.text.y = element_text(size = 7), legend.text = element_text(size = 12))
  return(p)
}

createForestDF <- function(data, title){
  d <- data.frame(x=data$geneset.names, y=as.numeric(data$`estimate.odds ratio`), ylo = as.numeric(data$conf.int1), yhi = as.numeric(data$conf.int2), pval=as.numeric(data$p.value), numbGeneInPway = data$NumbGenesInMsigdbPathway,
                  numbMatches=data$MatchesBtwTargAndPathway)
  d$x <-factor(d$x, levels=d[order(-d$y), "x"])
  return(forestplot(d, title, xlab=expression("Odds Ratio("*log[10] *" scale)")))
}

gseWrapper <- function(B, Targ, genesets, geneset.names, qval, OR){
  #dir can equal "up" or "down" for up or down regulated, respectivtly
  res_gse <- genesetEnrichmentFisher(B = B, Targ = Targ, GS.GS = genesets, GS.names=geneset.names)
  res_gse_filt <- res_gse[as.numeric(res_gse$bhPval) < qval & as.numeric(res_gse$`estimate.odds ratio`) > OR & as.numeric(res_gse$conf.int1) > OR,]
  return(res_gse_filt)
}

plot.volcano.gsea = function(hallmarkKeggReactome, diffExprDf, geneset.name, log2FC_diffexp, qval_diffexp, ensg_map, brew.color.set1, propSubset = 1){
  entrezIdsGeneSet = hallmarkKeggReactome[[1]][[geneset.name]]
  diffExprDf$logFC_thresh = diffExprDf$logFC > log2FC_diffexp & diffExprDf$adj.P.Val < qval_diffexp
  diffExprDf2 = dplyr::left_join(diffExprDf, ensg_map, by= c("ensgId" = "Gene.stable.ID"))
  diffExprDf2$inGeneSet = diffExprDf2$NCBI.gene.ID %in% entrezIdsGeneSet
  
  #if(randSubset){ #randomly downsample the # of genes for visualization purposed
  diffExprDf2$propSubset = TRUE
  if(propSubset < 1){
    diffExprDf2$propSubset <- sample(c(TRUE, FALSE), prob=c(propSubset, 1-propSubset), size=nrow(diffExprDf2), replace=TRUE)
  }
  diffExprDf2$NCBI.gene.ID %in% sample(entrezIdsGeneSet, ceiling(propSubset*length(entrezIdsGeneSet)), replace=FALSE)
  #}
  #should make this a threshold for the labels, not the color of dots
  
  colors_set1 = RColorBrewer::brewer.pal("Set1", n = 9) #green is 3, blue is 2
  ggplot(diffExprDf2, aes(x = logFC, y = -log(adj.P.Val, 10), label = HGNC.symbol, color = logFC_thresh & inGeneSet)) +
    theme_bw() + geom_vline(xintercept = c(-1,1), linetype="dashed", color = "grey") + 
    geom_hline(yintercept = -log(qval_diffexp,10), linetype="dashed", color = "grey") +
    geom_point(aes(alpha = ifelse(logFC_thresh, 0.3, 0.25))) + 
    ggrepel::geom_text_repel(aes(label=ifelse(inGeneSet & logFC_thresh & propSubset, 
                                              as.character(HGNC.symbol),''))) +
    scale_color_manual(values = colors_set1[c(9,brew.color.set1)]) + 
    theme(axis.text  = element_text(size=14)) + 
    labs(size = 14, x = expression(log[2](Fold~Change)), y = (expression(-log[10](q~value)))) + 
    ggtitle(paste0("Volcano Plot Highlights Upregulated Genes Within the\n", stringr::str_to_title(geneset.name, locale = "en"), " Geneset"))
}


dupMean <- function(segMean, id){
  result <- split(segMean, id, drop = F)
  result2 <- plyr::laply(result, mean)
  names(result2) <- names(result)
  return(result2)  
}

returnCNVvalues <- function(cnvGr, gene_Gr){
  overLaps = subsetByOverlaps(cnvGr, gene_Gr) #1 gene across all pts
  #take the average of any two that have the same tcga_id
  vectNoDupl <- dupMean(segMean = overLaps$segment_mean, overLaps$tcga_id)
  return(vectNoDupl)
}

fillNAwithColMeans <- function(data){
  for(i in 1:ncol(data)){
    data[is.na(data[,i]), i] <- mean(data[,i], na.rm = TRUE)
  }
  return(data)
}

logRegElNetLooc <- function(x, y, alpha, retGlmNt=FALSE){
  #creates a matrix of coefficients using elastic net regression.
  CV <- cv.glmnet(x, y, family="binomial", type.measure = "class", alpha = alpha, nlambda = 100, nfolds = length(y)) #this is Leave one out Cross validation
  fit <- glmnet(x, y,family="binomial", alpha = alpha, lambda = CV$lambda.1se)
  coeffic <- coef(fit) #get the coefficients from the fit model 
  if(retGlmNt){
    return(fit)
  }
  return(coeffic)
}

sig2star <- function(s, breaks=c(-Inf, 0.001,0.01,0.05,1), 
                     labels=c("***","**","*","")) {
  r <- s
  r[] <- as.character(cut(s, breaks=breaks, labels)) 
  r[is.na(r)] <- ""
  r
}

sig2star01 <- function(s, breaks=c(-Inf, 0.0001,0.001,0.01,1), 
                       labels=c("***","**","*","")) {
  r <- s
  r[] <- as.character(cut(s, breaks=breaks, labels)) 
  r[is.na(r)] <- ""
  r
}

getExonsInGene <- function(allExons, gene_gr){
  exonsInGene <- allExons %>% 
    subsetByOverlaps(gene_gr) %>% unlist() %>%
    IRanges::reduce() %>% sort()
  
  if(unique(strand(exonsInGene)) == "+"){
    exonsInGene$Exon_order = paste0("Exon_middle", 0:{length(exonsInGene)-1})
    exonsInGene$Exon_order[length(exonsInGene)] = "Exon_last"
    exonsInGene$Exon_order[1] = "Exon_first"
  } else if(unique(strand(exonsInGene)) == "-"){
    exonsInGene$Exon_order = "Exon_middle"
    exonsInGene$Exon_order[1] = "Exon_last"
    exonsInGene$Exon_order[length(exonsInGene)] = "Exon_first"
  }
  return(exonsInGene)
}

methStatusAtLoci <- function(geneGr, methSE){
  #methSE = Methylation summarized experiment object with GRanges 
  #object as first column
  meth_subst = subsetByOverlaps(methSE, geneGr)
  return(meth_subst)
}

findAndAnnotateCpGsInGene <- function(gene_gr, allExons, methSE, genes_gr_prom){
  #gene_gr has the coordinates of the gene within which CpGs are to be searched for
  #allExons is a txDB object that contains all the exons
  #methSE is a genomicranges object that contains all the CpGs
  cpgsInGene <- subsetByOverlaps(methSE, gene_gr)
  
  exonsInGene <- getExonsInGene(allExons, gene_gr)
  exonIndices <- findOverlaps(cpgsInGene, exonsInGene)
  cpgInExon <- cpgsInGene[queryHits(exonIndices)]
  cpgInExon$location <- exonsInGene[subjectHits(exonIndices)]$Exon_order
  
  cpgInProm <- subsetByOverlaps(cpgsInGene, genes_gr_prom) 
  if(length(cpgInProm) > 0) {cpgInProm$location <- "Promoter"}
  
  cpgInIntron <- cpgsInGene[!cpgsInGene$cgid %in% c(cpgInProm$cgid, cpgInExon$cgid)]
  if(length(cpgInIntron) > 0) {cpgInIntron$location <- "Intron"}
  
  cpgTot <- sort(c(cpgInExon, cpgInIntron, cpgInProm))
  return(cpgTot)  
}

range01 <- function(x){(x-min(x, na.rm = TRUE))/(max(x, na.rm = TRUE)-min(x, na.rm=TRUE))}


scatterMixPlot3 <- function (mmParams, selIndThresh = 1, geneLabels = NULL, sizesX, sizesY, ...) {
  mmParams <- as.data.frame(mmParams)
  oneOverAlpha <- diff(range(mmParams$deltaMu2))
  alpha1 <- 1/oneOverAlpha
  quants <- c(0.05, 0.5, 0.95)
  colorsRed <- RColorBrewer::brewer.pal(n = length(quants), 
                                        name = "Reds")
  deltaMu2Quant <- stats::quantile(mmParams[, "deltaMu2"], 
                                   quants)
  deltaMu1Quant <- stats::quantile(1/(abs(mmParams[, "deltaMu1"]) + 
                                        alpha1), quants)
  deltaMu2 <- deltaMu1 <- NULL
  x <- ggplot(data = as.data.frame(mmParams), aes(x = deltaMu2, 
                                                  y = 1/(abs(deltaMu1) + alpha1))) + theme_classic() + 
    geom_hline(yintercept = deltaMu1Quant, col = colorsRed, 
               size = sizesY) + geom_vline(xintercept = deltaMu2Quant, 
                                           col = colorsRed, size = sizesX) + geom_point(alpha = 0.5) + 
    xlab(expression(paste(Delta, mu[2]))) + ylab(expression(paste(frac(1, 
                                                                       paste(Delta, mu[1], " + ", alpha)))))# + ...
  if (selIndThresh < 1) {
    mmParamsSi <- mmParams[mmParams$SI > selIndThresh, ]
    x <- x + geom_point(data = as.data.frame(mmParamsSi), 
                        aes(x = deltaMu2, y = 1/(abs(deltaMu1) + alpha1)), 
                        size = 10, alpha = 0.25, col = colorsRed[length(colorsRed)], 
                        fill = colorsRed[length(colorsRed)]) + ggtitle(bquote(Distribution ~ 
                                                                                of ~ Mixture ~ Model ~ Parameters * "," ~ alpha ~ 
                                                                                "=" ~ .(round(alpha1, 2)) * ", SI >" ~ .(selIndThresh))) + ...
  }

  
  else if (!is.null(geneLabels)) {
    mmParamsSi <- mmParams[geneLabels, ]
    mmParamsSi$geneLabels <- geneLabels
    x <- x + geom_point(data = as.data.frame(mmParamsSi), 
                        aes(x = deltaMu2, y = 1/(abs(deltaMu1) + alpha1)), 
                        size = 10, alpha = 0.25, col = colorsRed[length(colorsRed)], 
                        fill = colorsRed[length(colorsRed)]) + ggrepel::geom_label_repel(data = mmParamsSi, 
                                                                                         aes(x = deltaMu2, y = 1/(abs(deltaMu1) + alpha1)), 
                                                                                         label = rownames(mmParamsSi), alpha=0.75, color="black") + ggtitle(bquote(Distribution ~ 
                                                                                                                                                                     of ~ Mixture ~ Model ~ Parameters * "," ~ alpha ~ 
                                                                                                                                                                     "=" ~ .(round(alpha1, 2)))) + ...
  }
  else {
    x <- x + ggtitle(bquote(Distribution ~ of ~ Mixture ~ 
                              Model ~ Parameters * "," ~ alpha ~ "=" ~ .(round(alpha1, 
                                                                               2)))) + ...
  }
  return(x)
}

gg__bar = list(
  theme_classic(),
  theme(legend.position="top",
        axis.ticks = element_blank(),
        axis.text = element_text(size=16, face="italic"),
        axis.text.x = element_text(angle = 90, hjust=1,vjust=0.5)),
  #axis.text.y = element_text(vjust=0.5, hjust = 1)),
  scale_y_continuous(expand = c(0,0))) 

gg__tile = list(
  theme_classic(),
  theme(legend.position="top",
        axis.ticks = element_blank(),
        axis.text = element_text(size=16, face="italic"),
        axis.text.x = element_text(angle = 90, hjust=1,vjust=0.5),
        axis.line = element_blank()),
  coord_fixed()) #,
  #axis.text.y = element_text(vjust=0.5, hjust = 1)),
  #, scale_y_continuous(expand = c(0,0))) 

gg__point = list(
  theme_classic(),
  theme(legend.position="top",
        axis.ticks.x = element_blank(),
        axis.text = element_text(size=16, color="black"),
        #axis.line = element_blank()
        )) 


#' Cluster and Reorder Rows and Columns
#'
#' Clusters the rows and columns of a numeric matrix using hclust
#'
#' @param matr matrix to be clustered
#' @param dimen Indicates the dimension that the clustering is performed in. One of either "row", "column", or "both". 
#'
#' @return returns a matrix of clustered values by either row, column, or both, 

clusterAndReorder <- function(matr, dimen="both"){
  #dimen can be one of row, column or both
  if(dimen == "row"){
    hcl <- hclust(dist(matr))
    matr.new <- matr[hcl$order,]
    return(matr.new)
  } else if(dimen == "column"){
    hcl <- hclust(dist(t(matr)))
    matr.new <- matr[,hcl$order]
    return(matr.new)
  } else if(dimen == "both"){
    hcl.r <- hclust(dist(matr))
    hcl.c <- hclust(dist(t(matr)))
    matr.new <- matr[hcl.r$order, hcl.c$order]
    return(matr.new)
  }
}

clusterDendro <- function(matr, dimen="row"){
  #dimen can be one of row, column or both
  if(dimen == "row"){
    hcl <- hclust(dist(matr))
    return(hcl)
  } else if(dimen == "column"){
    hcl <- hclust(dist(t(matr)))
    return(hcl)
  }
}


plotGeneHistNoProbCrv <- function (mmParams, exprNml, exprTum, isof, ...) {
  exprNml <- toMatrix(exprNml)
  exprTum <- toMatrix(exprTum)
  tidyDf <- as.data.frame(cbind(as.numeric(c(exprTum[isof, 
                                                     ], exprNml[isof, ])), as.factor(c(rep("tumor", ncol(exprTum)), 
                                                                                       rep("normal", ncol(exprNml))))), stringsAsFactors = FALSE)
  colnames(tidyDf) <- c("expr", "type")
  expr <- type <- ..density.. <- NULL
  p1 <- ggplot(tidyDf, aes(x = expr, color = as.factor(type), 
                           fill = as.factor(type), group = as.factor(type))) + theme_classic() + 
    geom_histogram(data = subset(tidyDf, type == 1), fill = "#F8766D", 
                   alpha = 0.2, aes(y = ..density..), binwidth = 0.2) + 
    geom_histogram(data = subset(tidyDf, type == 2), fill = "#00BFC4", 
                   alpha = 0.2, aes(y = ..density..), binwidth = 0.2) + 
    geom_rug(alpha = 0.3, show.legend = FALSE) + theme(axis.title.y = element_blank(), 
                                                       axis.text.y = element_blank(), axis.ticks.y = element_blank(), 
                                                       legend.position = "none", plot.title = element_text(size = 12), 
                                                       axis.text = element_text(size = 8), axis.title = element_text(size = 8)) + 
    ggtitle(paste0(isof, " : SI = ", round(mmParams[isof, 
                                                    "SI"], 4))) + xlab(expression(Log[2] * "(TPM Reads)")) + ...
  print(p1)
}


rescale <- function (x, newrange) {
  if (missing(x) | missing(newrange)) {
    usage.string <- paste("Usage: rescale(x,newrange)\n", 
                          "\twhere x is a numeric object and newrange is the new min and max\n", 
                          sep = "", collapse = "")
    stop(usage.string)
  }
  if (is.numeric(x) && is.numeric(newrange)) {
    xna <- is.na(x)
    if (all(xna)) 
      return(x)
    if (any(xna)) 
      xrange <- range(x[!xna])
    else xrange <- range(x)
    if (xrange[1] == xrange[2]) 
      return(x)
    mfac <- (newrange[2] - newrange[1])/(xrange[2] - xrange[1])
    return(newrange[1] + (x - xrange[1]) * mfac)
  }
  else {
    warning("Only numeric objects can be rescaled")
    return(x)
  }
}


plotGeneHistUD <- function(mmParams, exprNml, exprTum, isof, linesz=1.5, ...){

    exprNml <- toMatrix(exprNml)
    exprTum <- toMatrix(exprTum)

    tidyDf <- as.data.frame(cbind(as.numeric(c(exprTum[isof,], exprNml[isof,])),
        as.factor(c(rep("tumor",ncol(exprTum)), rep("normal",ncol(exprNml))))),
        stringsAsFactors=FALSE)
    colnames(tidyDf) <- c("expr", "type")
    expr <- type <- ..density.. <- NULL # Setting the variables to NULL first
    p1 <- ggplot(tidyDf, aes(x=expr, color=as.factor(type),
        fill=as.factor(type),
        group=as.factor(type))) +
    theme_classic() +
    geom_histogram(data=subset(tidyDf,type == 1),fill="#F8766D",
        alpha=0.2, aes(y=..density..), binwidth = .2) +
    geom_histogram(data=subset(tidyDf,type == 2),fill="#00BFC4",
        alpha=0.2, aes(y=..density..), binwidth = .2) +
    geom_rug(alpha=0.3, show.legend=FALSE) +
    theme(axis.title.y=element_blank(),
        axis.text.y=element_blank(),
        axis.ticks.y=element_blank(),
        legend.position="none",
        plot.title=element_text(size=12),
        axis.text=element_text(size=8),
        axis.title=element_text(size=8),
        axis.line.y=element_blank()) +
    ggtitle(paste0(isof, " : SI = ",
        round(mmParams[isof,"SI"],4))) +
    xlab(expression(Log[2] *"(TPM Reads)")) +
    stat_function(fun="dnorm", colour="#F8766D",
        args=list(mean=mmParams[isof,"nMu2"],
        sd=sqrt(mmParams[isof,"nVar"])), size=linesz) +
    stat_function(fun="dnorm", colour="#F8766D",
        args=list(mean=mmParams[isof,"nMu1"],
        sd=sqrt(mmParams[isof,"nVar"])), size=linesz) +
    stat_function(fun="dnorm", colour="#00BFC4",
        args=list(mean=mmParams[isof,"tMu2"],
        sd=sqrt(mmParams[isof,"tVar"])), size=linesz) +
    stat_function(fun="dnorm", colour="#00BFC4",
        args=list(mean=mmParams[isof,"tMu1"],
        sd=sqrt(mmParams[isof,"tVar"])), size=linesz) + ...
    print(p1)
    return(p1)
}

plotGeneHist2 <- function (mmParams, exprNml, exprTum, isof, txtSize = 10){
    exprNml <- toMatrix(exprNml)
    exprTum <- toMatrix(exprTum)
    tidyDf <- as.data.frame(cbind(as.numeric(c(exprTum[isof, 
        ], exprNml[isof, ])), as.factor(c(rep("tumor", ncol(exprTum)), 
        rep("normal", ncol(exprNml))))), stringsAsFactors = FALSE)
    colnames(tidyDf) <- c("expr", "type")
    expr <- type <- ..density.. <- NULL
    p1 <- ggplot(tidyDf, aes(x = expr, color = as.factor(type), 
        fill = as.factor(type), group = as.factor(type))) + theme_classic() + 
        geom_histogram(data = subset(tidyDf, type == 1), fill = "#F8766D", 
            alpha = 0.2, aes(y = ..density..), binwidth = 0.2) + 
        geom_histogram(data = subset(tidyDf, type == 2), fill = "#00BFC4", 
            alpha = 0.2, aes(y = ..density..), binwidth = 0.2) + 
        geom_rug(alpha = 0.3, show.legend = FALSE) + theme(axis.title.y = element_blank(), 
        axis.text.y = element_blank(), axis.ticks.y = element_blank(), 
        legend.position = "none", plot.title = element_text(size = 12), 
        axis.text = element_text(size = txtSize), axis.title = element_text(size = txtSize)) + 
        ggtitle(paste0(isof, " : SI = ", round(mmParams[isof, 
            "SI"], 4))) + xlab(expression(Log[2] * "(TPM Reads)")) + 
        stat_function(fun = "dnorm", colour = "#F8766D", args = list(mean = mmParams[isof, 
            "nMu2"], sd = sqrt(mmParams[isof, "nVar"]))) + stat_function(fun = "dnorm", 
        colour = "#F8766D", args = list(mean = mmParams[isof, 
            "nMu1"], sd = sqrt(mmParams[isof, "nVar"]))) + stat_function(fun = "dnorm", 
        colour = "#00BFC4", args = list(mean = mmParams[isof, 
            "tMu2"], sd = sqrt(mmParams[isof, "tVar"]))) + stat_function(fun = "dnorm", 
        colour = "#00BFC4", args = list(mean = mmParams[isof, 
            "tMu1"], sd = sqrt(mmParams[isof, "tVar"])))
    print(p1)
}


clean_dataset <- function(e, ensg_to_prot){

  #returns adj normal and tumor dataframes
  
  #remove ffpe samples
  e <- e[,!e$is_ffpe]
  
  #add technical variables
  colData(e)$hosp <- substr(rownames(colData(e)), 6,7)
  colData(e)$seqPlate <- substr(rownames(colData(e)), 22,25)
  
  ###remove all metaData columns that are NA
  colData(e) <- colData(e)[,colSums(is.na(colData(e)))<nrow(colData(e))]

  ##TPM normalization, then log2(tpm+1) transform
  eu <- assay(e)
  eu <- scale(eu, center=FALSE, scale=colSums(eu)) * 1000000
  eu <- log(eu+1, 2)
  assay(e) <- eu

  #separate tumor and adjacent normal samples
  et <- e[,substr(colnames(e),14,15) == "01"] 
  en <- e[,substr(colnames(e),14,15) == "11"]
  #print("ok")

  #remove duplicates
  et <- et[,!duplicated(substr(colnames(et),1,12))] 
  en <- en[,!duplicated(substr(colnames(en),1,12))] 
  #print("ok2")

  #drop transcripts that don't map to protein-coding ensembl gene Ids
  et <- et[rownames(et) %in% ensg_to_prot$Gene.stable.ID ,]
  en <- en[rownames(en) %in% ensg_to_prot$Gene.stable.ID ,]
  print(dim(et)); print(dim(en))

  #remove patients with NA values
  et <- et[,!apply(assay(et), 2, anyNA)] 
  en <- en[,!apply(assay(en), 2, anyNA)] 
  
  #Filter out genes where <20% of patients have a non zero expression value
  et <- et[rowSums(assay(et)==0)<=ncol(et)*.20,] 
  en <- en[rowSums(assay(en)==0)<=ncol(en)*.20,] 
  print(dim(et)); print(dim(en))

 
  #get the genes in common between the two
  genes_in_common <- intersect(rownames(et), rownames(en))
  et <- et[genes_in_common,]
  en <- en[genes_in_common,]
  #print("ok4")

  return(list(et=et, en=en))
}


filt_genes_pi_si <- function(m, pi_min, si){ 
## m is the mmp object
## pi is the minimum proportion of samples per group
## si is the selectivity index

  gene_filt_pi_si <- rownames(m)[m$tPi1 < (1-pi_min) & m$tPi1 > pi_min & 
                   m$nPi1 < 0.8 & m$nPi1 > pi_min & m$SI > si]
  return(gene_filt_pi_si)
}

filt_mmp_pi <- function(m, pi_min){ 
## m is the mmp object
## pi is the minimum proportion of samples per group

  m_filt <- m[m$tPi1 < (1-pi_min) & m$tPi1 > pi_min & 
                   m$nPi1 < 0.8 & m$nPi1 > pi_min,]
  return(m_filt)
}

 
plotOncomixScores <- function(mmp_dat, si=0.99, alph=0.8){
 reds <- brewer.pal(n = 9, name = "Reds")
 p <- ggplot(mmp_dat, aes(x=score, y=..density.., fill=SI > si)) +
   geom_histogram(data=subset(mmp_dat, SI<si), 
                  aes(x=score, y=..density.., fill=SI > si), 
                  binwidth = 0.1, color="grey", alpha=alph) + 
   geom_histogram(data=subset(mmp_dat, SI>si), 
                  aes(x=score, y=..density.., fill=SI > si), 
                  binwidth = 0.1, color="grey", alpha=alph) +
   theme_classic() + 
   scale_fill_manual(values=reds[c(3,7)]) + 
   scale_y_continuous(expand = c(0, 0)) + 
   theme(text=element_text(size=20, color="black"), legend.position="none") + 
   ylab("Density") + xlab("Oncomix Score")
 print(p)
}

pvalFxn <- function(survDifObj){
  p.val <- 1 - pchisq(survDifObj$chisq, length(survDifObj$n) - 1)
  return(p.val)
}

#### below are the simulation scripts ####

ret_mean_sd <- function(dat){
  dat2 <- mclust::Mclust(data = dat, G = 1, modelNames = "E", verbose = FALSE)
  mean <- dat2$parameters$mean
  std_dev <- sqrt(dat2$parameters$variance$sigmasq)
  return(list("mean"=mean, "std_dev"=std_dev))
}

ret_mean_sd_multiv_Gauss <- function(dat){
  dat2 <- mclust::Mclust(data = dat, G = 1, verbose = FALSE)
  mean <- dat2$parameters$mean
  std_dev <- sqrt(dat2$parameters$variance$sigmasq)
  return(list("mean"=mean, "std_dev"=std_dev))
}


#given the parameters, calc the score
score_from_params <- function(SI, deltaMu2, deltaMu1, nVar, tVar){
  score <- SI * {(deltaMu2 - deltaMu1) - (nVar + tVar)}
  return(score)
}    

score_from_params2 <- function(params){
  score <- params$SI * {(params$deltaMu2 - params$deltaMu1) - (params$nVar + params$tVar)}
  return(score)
}    

mean_sd_score_params <- function(dat){
  five_vars <- c("deltaMu2", "deltaMu1", "SI", "nVar", "tVar")
  res <- lapply(five_vars, function(x) ret_mean_sd(dat[[x]]))
  names(res) <- five_vars
  return(res)
}


mean_sd_score_params_multiv <- function(dat){
  four_vars <- c("deltaMu2", "deltaMu1", "nVar", "tVar") #"SI", 
  params <- dat %>% dplyr::select(four_vars) %>% mclust::Mclust(G=1, verbose = FALSE)
  m <- as.numeric(params$parameters$mean)
  v <- params$parameters$variance$sigma[,,1]
  si_vals <- dat$SI
  return(list(mean=m, cov=v, si_vals=si_vals)) #mean, covariance, si values for resampling
}

rand_draw_gauss <- function(top_params, n_draws){
  res <- lapply(top_params, function(x) rnorm(n = n_draws, mean = x$mean, sd = x$std_dev))
  return(res)
}

rand_draw_gauss_multiv <- function(params, n_draws){
  res <- MASS::mvrnorm(n = n_draws, mu = as.numeric(params$mean), Sigma = params$cov) %>% 
    as.data.frame()
  res$SI <- sample(x = params$si_vals, size = n_draws, replace = TRUE) #resampling for SI, this is non-Gaussian (range 0-1). Also encountered difficulties fitting parameters for Beta distribution
  return(res)
}

draw_rand_scores <- function(mmp, n_draws){
  mmp %>% 
    mean_sd_score_params() %>% 
    rand_draw_gauss(n_draws = n_draws) %>%
    score_from_params2()
}

draw_rand_scores_multiv <- function(mmp, n_draws){
  sc <- mmp %>% 
    mean_sd_score_params_multiv() %>% 
    rand_draw_gauss_multiv(n_draws = n_draws) %>%
    score_from_params2()
}


pval_effect_size <- function(top, not){
  res <- t.test(top, not, alternative = "greater")
  effect_size <- mean(top) - mean(not)
  pval <- res$p.value
  return(c("pval" = pval, "effect_size" = effect_size))
}

calc_rand_pval_effect_size <- function(top, not){
  top_rand_scores <- draw_rand_scores(top, n_draws=nrow(top))
  not_rand_scores <- draw_rand_scores(not, n_draws=nrow(not))
  pval_effect_size(top_rand_scores, not_rand_scores)
}

calc_rand_pval_effect_size_multiv <- function(top, not){
  top_rand_scores <- draw_rand_scores_multiv(top, n_draws=nrow(top))
  not_rand_scores <- draw_rand_scores_multiv(not, n_draws=nrow(not))
  pval_effect_size(top_rand_scores, not_rand_scores)
}

#try sampling from the joint distribution


read.gct <-
  function (file) {
    expr = read.table(file, skip = 2, header = TRUE, sep = "\t", quote = "")
    rownames(expr) = expr[,1]
    
    checkName = table(expr[,1])
    if(max(checkName) > 1) {
      stop(paste("Genes in gct file should be unique: ", names(which.max(checkName)), sep = " "))
    }
    expr = expr[,-c(1,2)]
    expr = as.matrix(expr)
    
    return(expr)
  }
```
